# Youth-led co-creation of a non-communicable disease risk reduction intervention in urban informal settlements of Karachi, Pakistan: a participatory action research

**DOI:** 10.1136/bmjph-2026-005286

**Published:** 2026-11-05

**Authors:** Sabahat Naz, Abiyah Narmeen, Ghazala Shaikh, Rashida Rasheed, Maira Mubashir, Romaina Iqbal

**Affiliations:** 1 Community Health Sciences, The Aga Khan University, Karachi, Pakistan

**Keywords:** Adolescent, Community-Based Participatory Research, Public Health, Qualitative Research

## Abstract

**Introduction:**

Risk factors for non-communicable diseases (NCDs) are often established during childhood and adolescence, underscoring the importance of early-life health behaviour interventions. Our study co-created a youth-led NCD risk-reduction intervention with school-going adolescents in the urban informal settlement of Karachi, Pakistan.

**Methods:**

This paper presents the co-creation phase of a broader pretest and post-test intervention study. The main study involved 40 adolescents aged 14 to 16 (grades 9 and 10) recruited from five public and five private schools. During the co-creation phase, focus group discussions (FGDs) were conducted to explore perceptions and priorities related to NCD risk reduction, in which 26 out of 40 youth (across three FGDs) and 18 parents (across two FGDs) participated. In-depth interviews (IDIs) were conducted with 21 key school stakeholders, including headteachers (n=5), class teachers (n=13) and canteen staff (n=3). To develop the intervention, we conducted a 2-day participatory workshop with youth and teachers. Insights from FGDs, IDIs and participatory workshops directly informed the intervention’s content, format and delivery methods.

**Results:**

In collaboration with youth and teachers, we co-created three core intervention components. An animated video titled ‘Youth Health Heroes—Rooshan Kal ki Umeed’ (Youth Health Heroes—Hope for a Bright Tomorrow) emphasised how adopting healthy habits early in life can prevent NCDs in adulthood. A structured physical activity session was designed to promote the integration of regular exercise into daily routines. Finally, a role-play titled ‘Jahan Sath Wahan Sehat’ (Where there is support, there is health) illustrated how peer support can positively influence health-related behaviours. Visual charts supported these components and reinforced key messages. The intervention was structured to be delivered in two 45-minute sessions within a week. A comprehensive training manual was developed to support standardised intervention delivery across schools.

**Conclusions:**

Our study co-created a contextually relevant intervention addressing key NCD risk factors among children and adolescents. It highlights the importance of youth engagement in intervention design and lays the groundwork for future implementation and evaluation, with the potential for adoption in other similar settings.

**Trial registration number:**

NCT06465771.

**WHAT IS ALREADY KNOWN ON THIS TOPIC**

**WHAT THIS STUDY ADDS**

- Using participatory action research, we co-created a youth-led NCD risk-reduction intervention, in collaboration with youth and other potential stakeholders, comprising an animated video, a physical activity session and a role-play.

**HOW THIS STUDY MIGHT AFFECT RESEARCH, PRACTICE OR POLICY**

- Youth-led, co-created interventions may provide useful insights for national school-based NCD prevention guidelines and adolescent health strategies in LMICs, promoting early prevention as a cornerstone of long-term NCD control.

## Introduction

 Many non-communicable diseases (NCDs) that develop in adulthood are linked to one or more modifiable risk factors, such as unhealthy diet, physical inactivity, tobacco use and overweight/obesity, which often develop during childhood and adolescence.^1,2^ For instance, a study conducted in Karachi, Pakistan, revealed that 58% of children had at least one modifiable risk factor of NCDs, with 29% being physically inactive, 31% exhibiting unhealthy eating habits, 21% being overweight and 6% being smokers.^3^ In addition, a survey conducted among students in grades 6 to 10 (n=501) in the Sindh province of Pakistan revealed that 80% of students chewed areca nuts and 61% used smokeless tobacco in the form of paan and gutka.^4^ These findings highlight the importance of targeted interventions for children and adolescents and offer an opportunity to promote healthy behaviours and reduce the risk of developing NCDs later in life.

Given this, there is growing recognition of the value of actively involving youth in health behaviour research.^5^ Engaging young people in identifying health challenges and co-creating relevant solutions can help ensure interventions are better tailored to their needs and more relevant and acceptable to the intended audience.^6^ In addition, youth involvement increases their visibility and voice in the broader community, drawing attention from key stakeholders and policymakers who shape health agendas.^2,7,8^ Participatory approaches, including Youth Participatory Action Research (YPAR),^9^ emphasise youth voice, shared decision-making and active involvement throughout the research and intervention-development process. Studies in different settings, for example, have involved youth and other key stakeholders at various stages of intervention development, from conceptualisation and refinement of intervention materials to implementation and delivery.^10–13^ These approaches have been shown to enhance youth ownership and engagement and can help contribute to the development of interventions that are relevant and acceptable to the target audience.^10–13^

Since NCDs share common modifiable risk factors, the existing literature highlights the potential value of co-creating intervention programmes that address multiple NCD-related health behaviours among adolescents.^7–10^ However, there is limited evidence on how young people and key stakeholders can collaboratively develop an integrated intervention that simultaneously addresses shared NCD risk factors in low-resource urban settings.^10–13^ This paper addresses this gap by presenting the co-creation phase of a broader preintervention and postintervention study.^14^ Specifically, we describe how we engaged grade 9 and 10 students and key stakeholders from public and private schools in the informal urban settlement of Karachi, Pakistan, to co-create a youth-led intervention addressing shared NCD risk factors. The resulting intervention was subsequently delivered by the participating youth to their younger peers in grades 5 and 6. Findings from the implementation and evaluation phases are beyond the scope of the present paper and will be reported separately.

## Materials and methods

### Study design and setting

This paper presents a detailed description of the intervention co-creation phase of a broader preintervention and post-intervention study,^14^ which was prospectively registered in the clinical trials registry (NCT06465771). We co-created the intervention between September 2024 and January 2025 in 10 public and private schools (five schools each).^14^ The schools are located in Azam Basti and Mehmoodabad, informal urban settlements in Karachi, serving low- and middle-income communities. 40 grade 9 and 10 students, aged 14 to 16, participated in the study. In addition, we purposively involved students’ parents, class teachers, head teachers and canteen staff in the co-creation process. The study’s protocol has been published previously.^14^

### Intervention co-creation process

The intervention co-creation involved iterative planning, reflection and collaboration to develop an intervention that was contextually relevant to the needs and circumstances of the participating schools and young people. The approach was based on the premise that involving young people and relevant stakeholders in intervention development can incorporate their contextual knowledge and experiences into the design process. We therefore integrated the steps of intervention mapping (IM)^15^ and YPAR.^9^ Incorporating the IM framework provided a structured, systematic approach for translating stakeholders’ perspectives into intervention components (figure 1). YPAR principles guided the active involvement of youth throughout the intervention development process. During the qualitative interviews with youth and key stakeholders, we identified knowledge gaps among youth and younger children, prioritised needs and discussed ideas about the intervention materials. In addition, youth contributed at multiple stages of the intervention development process by reviewing, selecting, refining and confirming the intervention content and materials through participatory workshops and engagement through a WhatsApp group.

**Figure 1:**
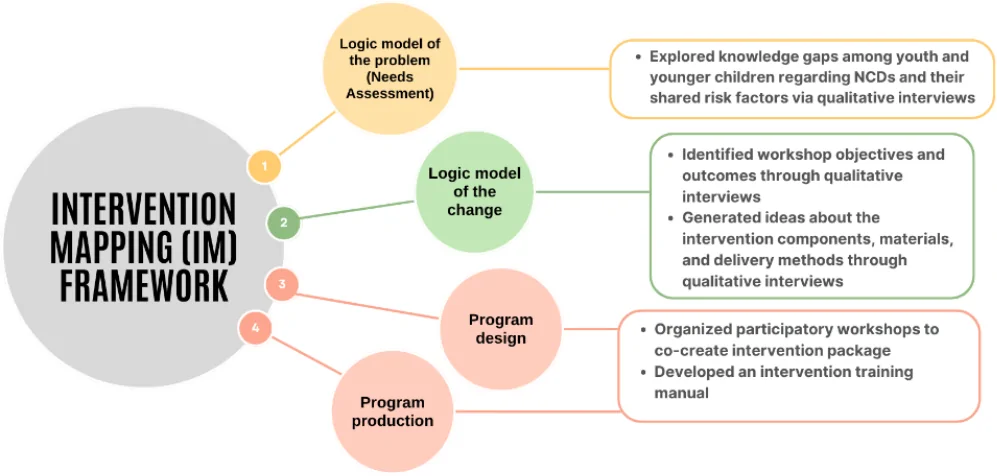
Intervention mapping (IM) framework to co-create a youth-led NCD risk reduction intervention. NCD, non-communicable disease.

### Qualitative interviews

#### Focus group discussions and in-depth interviews

Of the 40 youths enrolled in the main study, we conducted three focus group discussions (FGDs) with youths (n=26) and two FGDs with their parents (n=18) until data saturation was achieved. Before the interviews, we explained the study goals and objectives to all participants and gave them an opportunity to ask questions and seek clarification. During FGDs with youth, we used “Guess the Disease” as an icebreaker and incorporated games such as “Word Search (related to NCDs and their risk factors)” to create a friendly environment and foster open discussion. FGDs were held on school premises with prior permission from school authorities and parents. Each FGD lasted approximately 1 hour and 10 min.

We conducted in-depth interviews (IDIs) with the key stakeholders (n=21), including headteachers (n=5), schoolteachers (n=13) and canteen staff (n=3). Although we aimed to include more canteen staff in the IDIs, some schools lacked canteen facilities, and others declined to participate. AN (female; BS in Nutrition) and RR (female; BCom) were both Senior Research Assistants (SRAs) on the study team. AN conducted the FGDs and IDIs, while RR took detailed field notes during the interviews. Both researchers had prior experience and a good understanding of qualitative interviewing.

#### The topic guide for FGDs and IDIs

Following IM steps 1 and 2, the logic model of the problem and change (figure 1), the topic guide for FGDs and IDIs generally covered questions about the preferred intervention material, including themes, structure, components, sequence, content and delivery. We asked parents and other stakeholders about knowledge gaps among students regarding NCDs and their risk factors and about the feasibility of delivering the intervention in schools (see online supplemental file 1). In addition, we explored youth understanding of NCDs and their risk factors during the FGDs, which informed the planning of the workshop’s objectives, agenda, materials and outcomes (see online supplemental file 2).

#### Qualitative data analysis

We audio-recorded all interviews and transcribed them verbatim into Roman Urdu (Urdu written in the Latin alphabet) to retain participants’ original wording, expressions and conversational context for analysis, while removing identifiable information. We used a manual Deductive Thematic Analysis Framework approach, as described by Braun and Clarke.^16^ First, the researchers familiarised themselves with the data by repeatedly reading the transcripts. Two researchers generated the initial codes deductively using the study objectives and topic guides. Related codes were then organised into potential macrothemes and microthemes using a thematic framework developed in Excel, and these themes were reviewed against the coded data and the full dataset to assess coherence and relevance. The study team refined the themes through discussion. Once finalised, the themes were organised and presented along with relevant participant quotations. The transcripts were analysed in their original Roman Urdu form to retain participants’ original expressions and conversational meaning, and selected quotations were subsequently translated into English for presentation in the manuscript.

### Formation of WhatsApp group

Before the participatory workshops, we formed a WhatsApp group to facilitate communication with youth, their parents and schoolteachers. Because participating youth did not have their own mobile phones, we used parents’ phone numbers to add them to the group, and communication occurred under parental supervision. The group was used for logistical coordination, including sharing information flyers, workshop agendas, timely reminders and photographs of ongoing activities. It was also used to share draft intervention materials with youth for review and feedback as part of the iterative co-creation process. Communication through a WhatsApp group ensured active participation and improved youth attendance throughout the co-creation process.

### Participatory workshops

Once FGDs and IDIs were completed and their themes were finalised, we conducted a 2-day workshop with youth and schoolteachers to co-create an intervention programme. The first workshop aimed to build an understanding of NCDs and their risk factors among youth and to present our qualitative interview findings to finalise the intervention materials. The second workshop aimed to review the developed intervention with youth and obtain confirmation. The workshops were scheduled between December 2024 and January 2025, with a 17-day gap between the two workshops. We organised these co-creation workshops at two schools included in our study. Each workshop lasted approximately 3 hours.

31 out of 40 youths participated in the first workshop, along with six teachers, while six study team members facilitated the event, including three nutritionists, one medical doctor and two SRAs. During the second workshop, 23 youths and four teachers participated, and four study team members, including two nutritionists and two SRAs, facilitated the event. Facilitators worked collaboratively across sessions, including presentations on NCD-related topics, presenting and discussing qualitative findings, group activities, voting and prioritisation activities and developing and reviewing intervention materials. All facilitators were internal members of the study team.

During the first workshop, we discussed NCDs and their risk factors, explained the study’s phases and timeline, and provided examples of existing youth-led studies from different regions. One of our workshop facilitators led a physical activity session to help youth stay engaged. We also conducted a short multiple-choice pictorial quiz with the youth to assess their knowledge and understanding of the topic. During the workshop, we provided badges to ensure active participation and engagement throughout. To select the intervention material, we shared the opinions and ideas collected during the FGDs and IDIs using a PowerPoint presentation. We used a voting system that allowed youth to express their preferences on intervention components and structure (format, number of sessions, duration, frequency, etc) using ‘green’ (yes) and ‘red’ (no) flags. Youth could vote for multiple options, and we selected those with the most votes for inclusion in the final intervention. After voting, we divided the youth into smaller groups to brainstorm ideas for the intervention content. Youth were intentionally assigned to mixed groups comprising participants from both public and private schools to facilitate the exchange of ideas across school contexts and support the joint development of the intervention. Teachers and workshop facilitators supervised each group to ensure smooth group activities. Afterward, each group presented its ideas to the whole group.

Before conducting the second workshop, we used the interim period (17 days) to organise and develop the intervention content selected during the first workshop. During this period, we reviewed the youth’s ideas from the first workshop and began drafting the intervention materials with them.

During the second workshop, we presented the intervention materials prepared during the gap between the two workshops and discussed them with the youth to refine them and get confirmation. We also offered options for naming the intervention material and used the same voting system to finalise the selection. Table 1 presents the detailed workshop agenda.

**Table 1:** Detailed agenda for workshops

Goals	Description of the session	Material
First workshop: selecting the intervention material		
Introductory session	Welcome and introduction of the study team, youth and teachers over a healthy breakfast Distribution of identification cards and folders	Egg sandwich and flavoured milk Identification cards Folders with a notepad, pencil and workshop agenda
Understanding NCDs and risk factors	Discussion on NCD trends and burden in Pakistan Explanation of NCD risk factors and their relevance to youth and younger peers Emphasis on the role of youth in addressing NCDs Physical activity break	PowerPoint presentation using infographics
Sharing qualitative findings	Overview of existing youth-led interventions Quiz Presentation of qualitative findings Voting on intervention components	PowerPoint presentation using infographics Video Quiz Green/red voting flags
Group activities and presentations	Brainstorming session with healthy snacks Group presentations and feedback Planning for intervention content development Scheduling the second workshop Workshop wrap-up and vote of thanks	Chart papers, markers and coloured pencils A printed guide Banana
Second workshop: finalising the intervention		
Reviewing the developed intervention	Recap of day 1 activities Discussion on progress made between workshops with healthy snacks Presentation and feedback on intervention materials Voting on intervention content names Planning next steps Vote of thanks	PowerPoint presentation Videos Green/red voting flags A pack of different fruits

NCDs, non-communicable diseases.

### Patient and public involvement

Grade 9 and 10 students, their parents, school headteachers, teachers and canteen staff helped generate ideas for the intervention materials and components through FGDs and IDIs. Grade 9 and 10 students and their teachers were involved in the co-creation of the intervention programme.

## Results

### Participant demographics

Online supplemental tables 1, 2 present the demographic profiles of the 26 youths and 18 parents, respectively, who participated in the FGDs. Of the 26 youths, the majority were female (65%, n=17), with a mean age of 15.1±0.8 years, and were almost evenly represented from public (54%, n=14) and private (46%, n=12) schools. For the parental FGDs (n=18), participants were predominantly mothers (77.8%, n=14) and housewives (79%, 11 of 14 mothers), and were mostly from public schools (67%, n=12). Most parents had completed matriculation or intermediate education (83%, 15 of 18). We conducted 21 IDIs with school stakeholders, including headteachers, teachers and canteen staff. As detailed in online supplemental table 2, these stakeholders spanned a broad age range (19 to 70 years) and were predominantly female (71.4%, n=15), with a higher representation from public schools (14 IDIs).

### Findings based on FGDs, IDIs and participatory workshops

Figure 2 presents the main themes and their corresponding subthemes.

**Figure 2:**
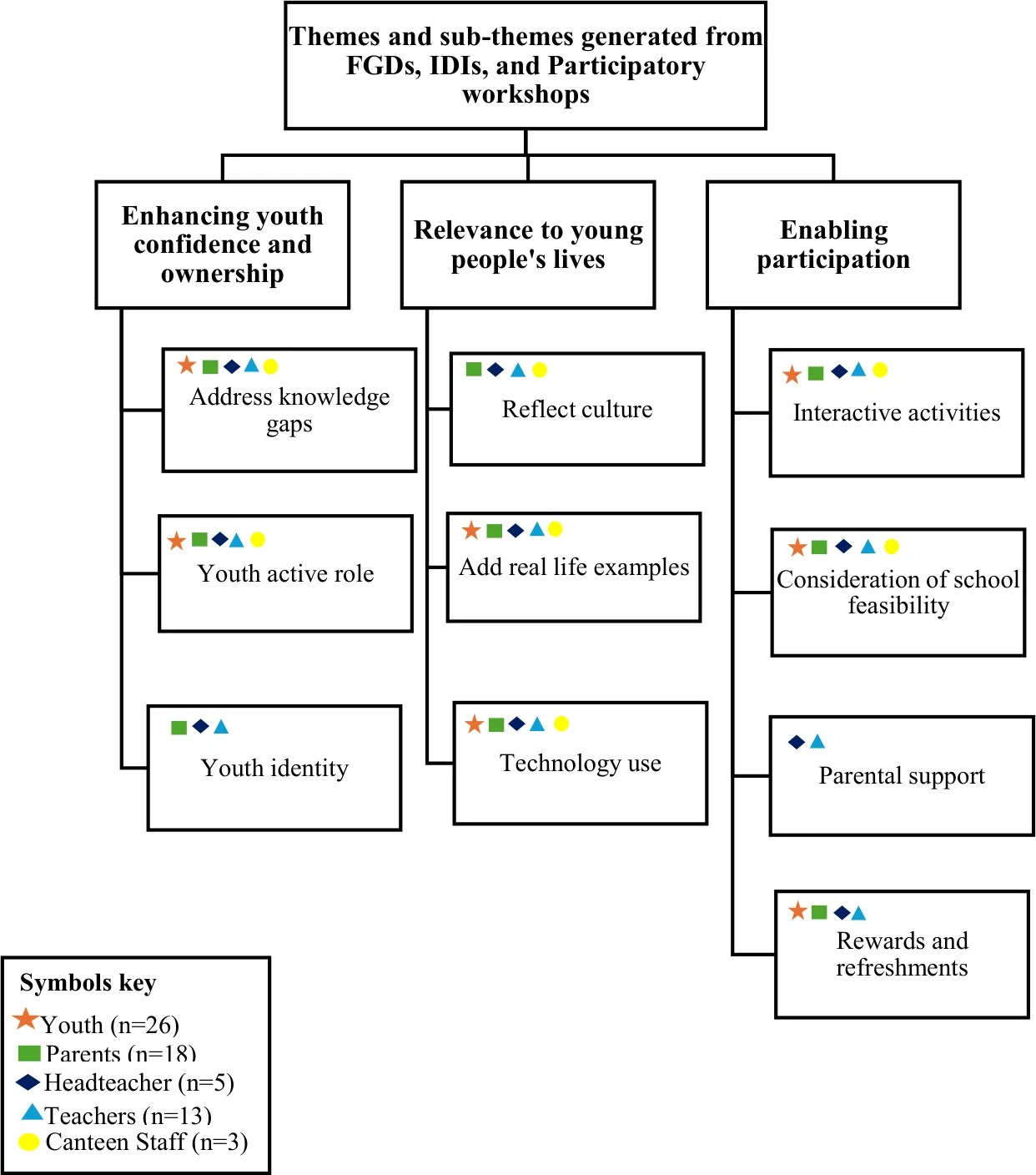
Themes and subthemes for qualitative interviews. FGDs, focus group discussions; IDIs, in-depth interviews.

#### Theme 1: enhancing youth confidence and ownership

Qualitative narratives from all stakeholder groups revealed that students across all grades had limited knowledge of NCDs and their risk factors. While formal science curricula introduce these terms, youth struggle to translate them into early prevention concepts and often consider that NCDs are adult diseases. In addition, qualitative analysis highlighted conceptual confusion where several youth mixed up NCDs with communicable infections, listing tuberculosis, chickenpox and hepatitis under the NCD umbrella.

There could be a misconception that such health issues are only for older people, so children might feel that they won’t face similar problems. (Teacher-female, IDI-11014, Public school, Age 25, 2 yrs of experience)

When asked about NCD risk factors, youth from private and public schools identified key lifestyle drivers, such as unhealthy diet, air pollution, tobacco use, stress and physical inactivity. An adolescent explained.

Right now, what comes to my mind is cancer. Cancer spreads through such things—like tobacco use and similar substances—which are very harmful to health. (Youth-male, FGD-30026, Private school, Age 16, Grade 10)

To bridge this gap, all stakeholders, including parents, teachers and canteen staff, strongly endorsed youth-led, peer-to-peer education. Canteen staff, parents and youth noted that younger children respond better to peer-led activities. In addition, youth and canteen staff highlighted that this approach would encourage youth to adopt healthy behaviours before promoting them to their peers.

If they (youth) don’t improve themselves first, they won’t understand later. They must change their behavior first; only then will they guide others. Once they set an example, children will automatically follow them. (Canteen staff-male, IDI-31020, Public school, Age 57, 35 yrs of experience)

In addition to supporting peer-to-peer education, headteachers, teachers and parents emphasised the importance of giving youth a strong identity to encourage ownership and responsibility for promoting healthy behaviours among younger peers and the wider community. One headteacher described a similar initiative in which students were given an identity and badge, which helped reinforce their sense of leadership and responsibility. She shared her students’ experiences and said:

As long as that badge remained close to their (students’) heart, they always carried it with them. It made them feel like leaders and reminded them of their responsibilities. They thought, They are ‘Kiran Sitara’, so it’s their job to track the wrong and work to eliminate it. (Headteacher-female, IDI–31019, Public school, Age 50, 34 yrs of experience)

We operationalised this conceptual link between advocacy and self-regulation during the participatory workshops, where we formally designated youth as ‘Youth Health Heroes (YHHs)’ and presented them with badges.

#### Theme 2: relevance to young people’s lives

All stakeholders, except the youth, suggested incorporating cultural relevance by showcasing religious events, festivals and everyday experiences to promote healthy behaviours and make the intervention more relatable to younger peers. They further recommended incorporating real-life stories to increase the intervention’s impact. In addition, they emphasised that engaging youth and younger peers through technology and up-to-date methods helps maintain their interest and enhance their learning.

Children will be very interested if, instead of just formal things, cultural elements are included and shown to them—they will enjoy it a lot…whether it’s related to Ramzan (a religious month) or any other occasion. (Headteacher-female, IDI-11016, Public school, Age 50, 24 yrs of experience)

#### Theme 3: enabling participation

Participants across all groups emphasised that conventional classroom-based health education may not sufficiently engage young people. They therefore favoured interactive, participatory approaches, including role play, storytelling, physical activity and animated videos, which they found more engaging and easier for younger students to understand. They also emphasised the importance of visual aids (charts, infographics) to maintain attention and enhance comprehension.

Have the students perform a tableau (role play) for grade 5. It shows a complete family where the children are eating unhealthy food. Suddenly, one of them falls ill. The family then takes the child to a doctor, who diagnoses the disease and explains its effects on the body. (Teacher-female, IDI-11014, Public school, Age 25, 2 yrs of experience)

Kids watch cartoons these days; if such education is included in cartoons, they will learn it effectively. (Father, FGD-41018, Public school, Age 42, Matriculation, Private job)

We should conduct activities that make them happy and increase their interest in learning these things. For example, physical activity should be included to help them understand it (the topic) better. (Youth-female, FGD-30025, Private school, Age 16, Grade 10)

Participants also emphasised considering school feasibility in terms of the number of intervention sessions, the duration and frequency of sessions, and the language of the intervention. Most participants recommended 2 to 3 sessions, each lasting 30–45 min, to avoid disrupting the school’s academic schedule and ensure students understand the topic well. They suggested delivering the intervention within a week to maintain younger peers’ interest. The responses regarding the intervention language were consistent among stakeholders, recommending a bilingual format with Urdu as the primary language, accompanied by simple English words to increase familiarity with English.

Approximately three sessions; in the first one, the child will feel unfamiliar and unable to digest immediately. However, in the second and third sessions, some familiarity develops, and they easily adapt to it. (Teacher-male, FGD-21008, Public school, Age 36, 10 yrs of experience)

Teachers and headteachers suggested informing parents about the intervention processes so that they can motivate their children (youth) and ensure active participation.

If we share a picture (program details) in the official group, the parents may take it seriously. Alternatively, we can discuss it in the PTM (Parent-Teacher Meeting) that we have engaged the children in this activity, so please listen to their thoughts and support them. (Teacher-female, IDI–11014, Public school, Age 25, 2 yrs of experience)

Teachers, headteachers and youth highlighted the importance of rewards, such as badges, certificates or small gifts like pens, to enhance their motivation and confidence and ensure active participation. Parents also suggested providing food during meetings and workshops to help youth feel refreshed and boost their energy levels.

If you give children medals or similar rewards, they become pleased. (Mother, FGD-21102, Public School, Age 33, Intermediate, Housewife)

During the first workshop, YHHs selected three activities from those suggested during FGDs and IDIs, giving the highest votes to the physical activity session (31/31), animated video (30/31) and role-play (29/31). In addition, they selected delivering these activities in two sessions (24/31), each 45 min long (24/31), within 1 week (24/31). All YHHs endorsed a bilingual delivery mode. Online supplemental table 3 presents the YHHs’ voting on different aspects of the intervention. The YHHs also provided detailed ideas on the chart papers for the intervention content through group activities, which we later used in designing the intervention. For instance, one group based on three youths (males), two from a public school and one from a private school (IDs-21014, 21034, 30005) suggested using charts to explain the benefits of physical activity during the session and highlighted that:

We need to exercise to maintain overall physical and mental health, including benefits like cardiovascular health, strengthened bones, weight management, reduced stress, better sleep quality, increased brain function, and decreased risk of NCDs.

Another group of four female students, two from private schools and two from public schools (IDs−20023, 20024, 11032, 31015), provided the central idea for the role-play, highlighting how peer support can help adopt healthy behaviours. They shared that:

In quitting a bad habit, not only does a person’s role matter, but there is also the support of some people behind them, through which they develop a good habit.

Other groups provided ideas for the animated video. For example, a group of three males from a public school (IDs 51020, 51039, 51040) shared that:

We want to tell the children that the mistakes these people make, like smoking, you should not make them; otherwise, they will become the cause of heart attacks (NCDs).

The co-creation process enabled youth to move from providing initial ideas during FGDs to actively shaping and producing intervention components during participatory workshops, and by the end of the first workshop, we had developed a basic intervention structure.

During the interim period, tasks were divided among study team members to facilitate intervention development while maintaining active youth involvement. Researcher RR compiled a list of classroom-friendly physical activities based on ideas the YHHs generated during the first workshop. Researcher AN led the development of the animated video, while Researcher SN developed the animated video and role-play scripts based on ideas the YHHs generated during the group activities. The draft scripts and animated video were shared with the YHHs through a WhatsApp group for iterative feedback and refinement, allowing them to assess whether the materials accurately reflected their perspectives and vision. During the second workshop, the YHHs watched the animated video, reviewed the role-play script and provided final feedback to refine it. YHHs tried all classroom-friendly physical activities we compiled during the interim period and made a final selection. For the animated video, YHHs divided into groups and recorded the voiceovers. They also developed posters highlighting the benefits of incorporating physical activities into daily life routines. Finally, they selected the names of the animated video and role-play using the same voting system. During the first workshop, YHHs scored an average of 6 out of 8 on the quiz, suggesting good comprehension of the key concepts presented. In addition, 18 YHHs received badges and 3 won prizes for obtaining the most badges. The finalised intervention components are presented as follows:

##### Role play

The role-play centres on peer and parental influence on youth behaviour. It centres on a character, Ali, a bright student who adopts unhealthy habits from his environment. Seeing his health and academic scores decline, his concerned friends intervene creatively by organising a celebration day at school. They also came up with the idea of launching a youth-led movement, ‘YHHs’, to raise awareness of healthy behaviours. Using visual charts to illustrate the distinction between healthy and unhealthy choices, the role-play concludes with an oath from younger peers to adopt and promote healthier lifestyles. The manual presents the detailed role-play script as online supplemental file 3). Youth named the role-play ‘Jahan Sath Wahan Sehat’ (Where there is support, there is health) (23/23) from the three options in online supplemental table 3.

##### Physical activity

The other intervention component was physical activity. We demonstrated a few shortlisted classroom-friendly physical activities, including ‘Heads, Shoulders, Knees and Toes’, ‘Arm circles’, ‘Simon Says’, ‘High Knee’ and ‘Hand Clap’. Among these, YHHs shortlisted ‘Heads, Shoulders, Knees and Toes’ (19/23), ‘Simon Says’ (19/23) and ‘Arm circles’ (18/23) (see online supplemental table 3). YHHs, teachers and the study team performed the selected physical activities in the classroom to ensure they were appropriate and feasible for the setting. Online supplemental file 3 provides a detailed description of the physical activities.

##### Animated video

The video highlights how adopting healthy habits early can prevent NCDs later in life (figure 3). Structured into four scenes, the video features YHHs explaining heart disease, a teacher addressing diabetes prevention and management, and a powerful story about cancer linked to chewing tobacco. It also includes a scene on lung disease, where an older man warns a teenage smoker about the consequences of smoking. The video concludes with the empowering message that youth can drive positive changes in their communities. Most importantly, the YHHs recorded the voiceover for the animated video during the second workshop. The link to the animated video is: https://youtu.be/Q_V21BoeFkY

**Figure 3:**
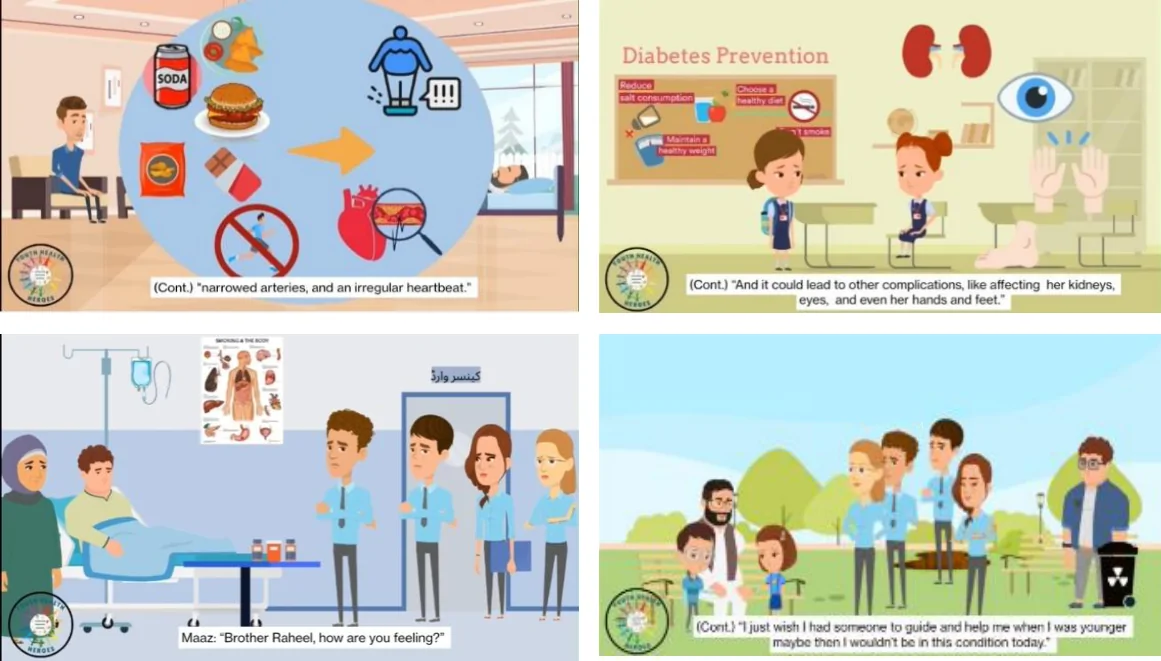
Glimpses of the animated video.

Youth named the animated video ‘Youth Health Heroes—Rooshan Kal ki Umeed’ (Youth Health Heroes–Hope for a bright tomorrow) (20/23) from the three options presented in online supplemental table 3.

##### Intervention training manual

We developed a training manual to guide YHHs throughout the intervention delivery (figure 4). The training manual included detailed instructions for each intervention component, along with facilitation methods and time allocation for each activity, to standardise intervention delivery across schools and help YHHs deliver it efficiently within their own schools. The complete manual is available as online supplemental file 3.

**Figure 4:**
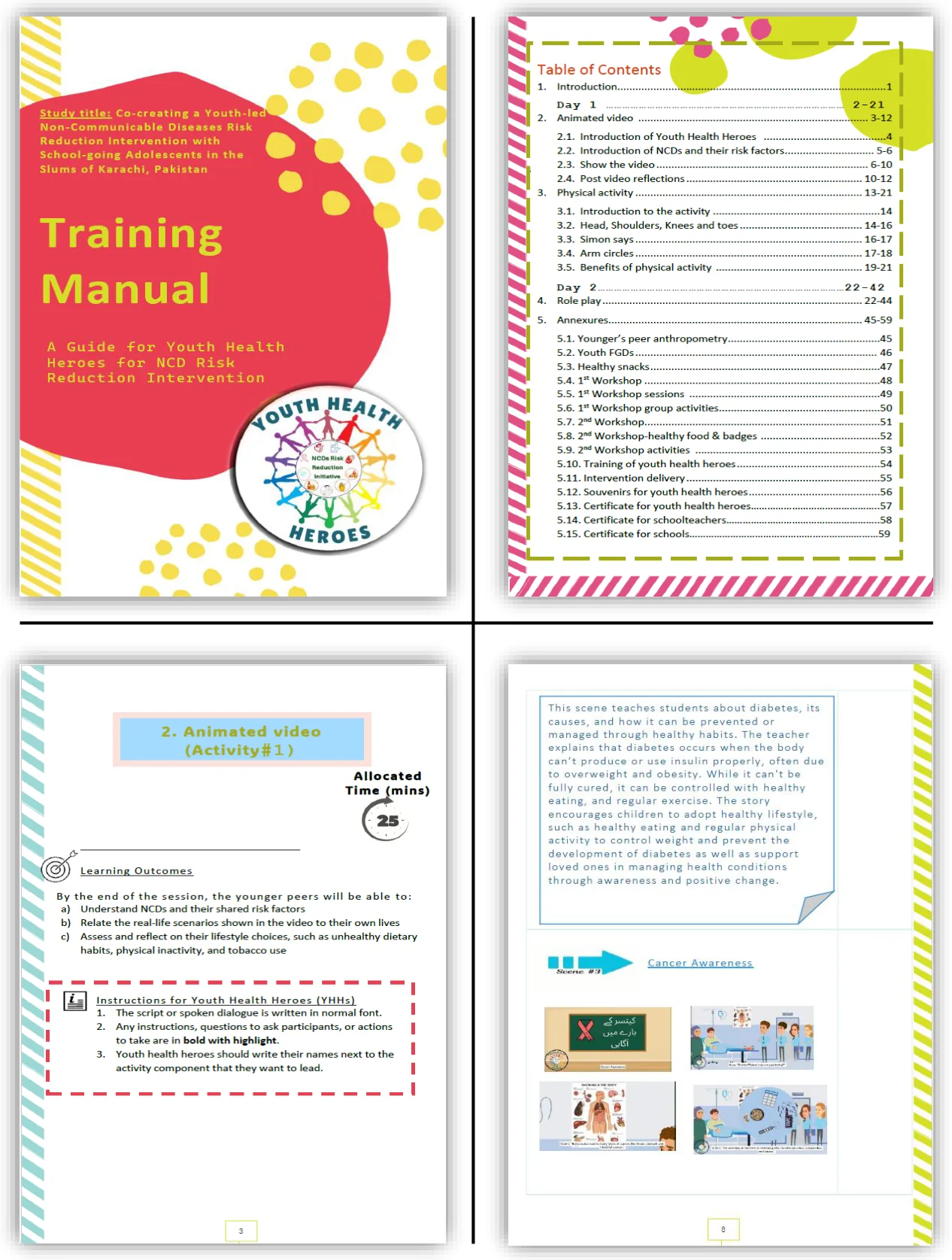
Highlights from the intervention training manual.

## Discussion

We co-created a youth-led NCD risk-reduction intervention in collaboration with YHHs and key stakeholders to ensure relevance. We used qualitative interviews, participatory workshops and ongoing engagement with youth through a WhatsApp group to co-create and iteratively refine the intervention materials and inform their delivery, drawing on the input, ideas and active contributions of youth and key stakeholders. The IM framework^15^ provided a systematic structure for intervention development, while YPAR principles^9^ supported active youth involvement in identifying priorities, generating ideas, reviewing materials and making decisions about the final intervention. The iterative process resulted in three core intervention components: an animated video, a physical activity session and a role-play, supported by visual materials and delivered over two 45-minute sessions within 1 week.

Literature supports the importance of engaging youth and relevant stakeholders in addressing health-related issues.^17^ For instance, a systematic review of participatory research involving children and youth on lifestyle-related childhood obesity demonstrated promising outcomes. The review found that youth engagement not only increased the intervention relevance but also led to significant improvements in at least one behavioural outcome, such as diet, physical activity or weight status. However, the review highlights variation across studies, depending on the strategies used and the extent of youth engagement.^17^ Similarly, a scoping review of adolescent participation in obesity-prevention research highlighted substantial variation in whether adolescents were consulted, involved collaboratively, or given greater influence over research decisions.^18^ In our study, both youth and other stakeholders generated ideas and provided suggestions during qualitative interviews. However, youth engagement extended beyond consultation, with YHHs actively involved in intervention development through participatory workshops and a WhatsApp group during the interim period. They identified priorities, generated ideas for intervention content, reviewed and refined intervention materials, participated in decisions about intervention components during the workshops, and helped name the intervention components. This iterative, sustained involvement gave youth opportunities to shape the intervention and refine its materials based on their perspectives and preferences.

Our approach shares similarities with the i-PROMISE study by Rawal *et al*,^10^ which developed an animated video, ‘Super Kid Aryan’, to promote healthy lifestyles among schoolchildren after obtaining feedback from students and teachers. While informative and engaging, children and teachers highlighted areas for improvement, such as addressing common myths, offering a Hindi version, and including real-life stories to make the content more relatable and impactful for students. We conducted extensive qualitative interviews with YHHs and key stakeholders to gather meaningful feedback and maximise the impact of our animated video. In addition, YHHs provided script ideas and reviewed successive versions of the script and video. The resulting video ‘Youth Health Heroes–Hope for a Bright Tomorrow’ is primarily in a local language (Urdu), with simple English words to aid comprehension. It addresses four major NCDs (cardiovascular disease, diabetes, cancer and lung disease) and their link to shared risk factors (unhealthy diet, physical inactivity and tobacco use). A distinct feature of our video is the inclusion of YHHs as animated characters, with voiceovers recorded by the YHHs, enhancing both relevance and ownership.

Recognising physical activity as a key risk factor for NCDs, stakeholders strongly recommended incorporating it as an intervention component during the qualitative interviews. YHHs and teachers strongly supported physical activity sessions and ranked them among their top priorities during the participatory workshop. Considering the school setting, classroom space and the students’ age group, YHHs selected three physical activities: ‘Heads, Shoulders, Knees and Toes’, ‘Simon Says’ and ‘Arm circles’. Other studies addressing obesity among school-going children, such as Healthy Buddies^11^ and the Eyto-Kids Project,^12^ also incorporated physical activity into their intervention programmes.

Most stakeholders identified role-play as a valuable activity, particularly given the resource-constrained context of selected schools. In addition, it has been widely recognised in the literature as a practical, interactive approach to delivering health-related education, particularly among children, as it promotes active learning, fosters empathy and enhances retention of key messages.^19,20^ Our role-play activity, titled ‘Where there is support, there is health’, centres on a relatable, real-life scenario in which a bright student, influenced by his environment, begins to adopt unhealthy behaviours. The student is guided back towards healthier choices through peer support and encouragement from friends. This approach reinforces the importance of positive peer influence and provides a practical, low-cost way to engage students in meaningful conversations about health within their social contexts.

Further, our intervention training manual served as an invaluable resource, designed to support consistent intervention delivery across schools. Inspired by the ‘I Support My Friends’ manual,^21^ which details participatory methods and the critical role that youth can play in community resilience and health promotion, our manual also provides instructions, scripts, activity guidance and facilitation techniques to help YHHs deliver the intervention. Similarly, the manual ‘Training of Youth Workers’^22^ highlighted approaches to equip youth with competencies such as leadership and teamwork. Our manual also served as a guide and a tool to instil confidence among the YHHs. A unique feature of our manual is that it includes step-by-step instructions and dialogues for each intervention component, ensuring standardised intervention delivery across schools.

Previous youth-led interventions have used specific identities to recognise participating adolescents and reinforce their role as peer educators. For example, in the EYTO project,^12^ the adolescents were designated as ‘Adolescent Challenge Creators (ACCs)’, while in the Canadian programme,^11^ they were named ‘Health Buddies’. Similarly, our stakeholders recommended this approach during the qualitative interviews, so we designated the participating youth as ‘YHHs’ throughout our study. This empowering identity extended beyond the badges they wore and was deeply embedded in our intervention components, such as animated video and role-play, highlighting their significant role in addressing NCD-related health behaviours.

A key logistical challenge during the co-creation process was bringing YHHs from 10 different schools together for participatory workshops. Addressing this challenge required close coordination with school principals and teachers, regular communication with parents through a WhatsApp group and transportation arrangements to facilitate students’ attendance. Providing a separate seating area for guardians at the workshop venue also helped accommodate parental concerns while allowing youth to participate in the activities. These experiences show that meaningful youth participation in co-creation requires more than providing opportunities to contribute; it also requires practical arrangements that address the logistical and family-related considerations affecting participation, particularly in resource-constrained settings.

After intervention development, YHHs were trained to deliver the intervention in their schools and then implemented it. While this progression shows the intervention moved beyond development to school-based delivery, its longer-term sustainability, including how long YHHs can continue activities independently and what support ongoing implementation requires, warrants further assessment. We will report on the training, implementation and sustainability aspects of the intervention separately.

Our study presents several notable strengths. The key strengths include an inclusive approach that involves youth and key stakeholders in the co-creation process to make the intervention relevant. In addition, we included youth from both public and private schools and brought them together during the co-creation workshops to facilitate the exchange of ideas and discussion across school contexts. This approach helped identify common priorities and informed the development of a single intervention package rather than separate versions for each school type. Another key strength was the integration of the IM framework and YPAR, which provided a structured approach to intervention development. Furthermore, the various components of the intervention, including an animated video, a role-play and a physical activity session, allow NCD risk-reduction messages to be delivered through multiple, interactive formats.

Despite its strengths, the study does have some limitations. Although we aimed to involve all youth in the workshop activities, participation decreased from 31/40 YHH in Workshop 1 to 23/40 in workshop 2 due to health issues, travel and unforeseen school commitments. As workshop 2 included the final refinement and voting on intervention components, the final decisions may have reflected more strongly the perspectives of youth who attended this workshop and may not fully represent those who were unable to participate. However, intervention development drew on multiple stages and data sources, including FGDs, IDIs, workshops and iterative feedback shared through the WhatsApp group. In addition, more girls (65%) than boys participated in the FGDs, and the qualitative data were not analysed by gender, as subgroup comparisons were beyond the scope of this co-creation study. However, both girls and boys participated in participatory activities that brought them together to incorporate diverse ideas into the intervention development process. Furthermore, the limited participation of fathers and canteen staff in the qualitative interviews may have limited the depth of insights considered during the intervention design. However, those who did participate offered suggestions and ideas that were well aligned with those from other stakeholder groups, which had already been integrated into the intervention. Since the intervention was developed with schools from informal urban settlements, these components may align more with the context of schools from similar settings. However, we incorporated multiple elements, such as an animated video that might align with the needs of high school students and can be easily adapted with minor adjustments for implementation in high school settings. Finally, although youth actively helped shape intervention content and refine materials, they were not directly responsible for independently producing all materials. This distinction is important when considering the practicalities of co-creation in low-resource settings. The intervention was developed with schools serving socioeconomically disadvantaged communities, where time constraints, competing school commitments and the considerable engagement required from both schools and students presented practical challenges to sustained participation. School and student dropouts further complicated this process. While involving youth directly in activities such as scriptwriting and video production could potentially strengthen ownership and relevance, these approaches require sufficient time, resources and appropriate technical support. Future co-creation initiatives could adopt a phased approach, giving youth opportunities to progressively take on more responsibility for material production through supported scriptwriting, peer-led content development and accessible digital tools. Such approaches may balance meaningful youth ownership with the practical constraints of developing interventions in low-resource settings.

In conclusion, we successfully co-created a youth-led intervention addressing the risk factors of NCDs among children and adolescents. Comprehensive interviews with key stakeholders and the active participation of youth throughout the co-creation process helped inform the development of a contextually relevant intervention. Future papers on the implementation and evaluation of this intervention will enhance the credibility of this work. In addition, the resulting intervention may provide a useful foundation for future adaptation and evaluation in similar low-resource settings, with potential for broader scale-up if feasibility and effectiveness are demonstrated.

## Supplementary material

10.1136/bmjph-2026-005286 online supplemental table 1

10.1136/bmjph-2026-005286 online supplemental table 2

10.1136/bmjph-2026-005286 online supplemental table 3

10.1136/bmjph-2026-005286 online supplemental file 1

10.1136/bmjph-2026-005286 online supplemental file 2

10.1136/bmjph-2026-005286 online supplemental file 3

10.1136/bmjph-2026-005286 online supplemental file 4

## Data Availability

Data are available on reasonable request.
