# Supplementary material for: Youth-led co-creation of a non-communicable disease risk reduction intervention in urban informal settlements of Karachi, Pakistan: a participatory action research
Source: BMJ Public Health. 2026 Nov 5;4(4):e005286. doi: 10.1136/bmjph-2026-005286 (PMC13756321; doi:10.1136/bmjph-2026-005286)
Supplement: Supplementary file 1 [file bmjph-4-4-s001.pdf]

Supplemental Table 1: Detailed characteristics of Youth who participated in FGDs

FGDs with Youth						
**Interview**	**School ID**	**School type**	**Participant ID**	**Grade**	**Age (years)**	**Sex**
**FGD 1**	21	Public	21013	9	15	Male
			21014		16	Male
			21033		16	Male
	50	Private	50009		14	Female
			50010		16	Female
			50029	10	15	Female
			50030		15	Female
**FGD 2**	11	Public	11011	9	14	Female
			11012		14	Female
			11032	10	16	Female
	20	Private	20003	9	16	Male
			20004		16	Female
			20023	10	16	Female
			20024		16	Female
	31	Public	31015	9	14	Female
			31016		14	Female
			31035	10	15	Female
			31036		15	Female
**FGD 3**	11	Public	11031	9	14	Female
	51		51019		14	Male
			51020		16	Male
			51039	10	15	Male
	30	Private	30005	9	16	Male
			30006		15	Female
			30025	10	16	Female
			30026		16	Male
