# Supplementary material for: Youth-led co-creation of a non-communicable disease risk reduction intervention in urban informal settlements of Karachi, Pakistan: a participatory action research
Source: BMJ Public Health. 2026 Nov 5;4(4):e005286. doi: 10.1136/bmjph-2026-005286 (PMC13756321; doi:10.1136/bmjph-2026-005286)
Supplement: Supplementary file 2 [file bmjph-4-4-s002.pdf]

Supplemental Table 2: Detailed characteristics of Stakeholders who participated in qualitative interviews

Interview	School ID	School type	Participant ID	Age (year)	Sex	Education	Occupation	Experience (years)	Position (Grade teacher)	Subject
**FGDs with Parents**										
**FGD 1**	30	Private	30111	40	Female	Grade 12	Housewife	Not applicable	Not applicable	Not applicable
			30112	30	Female	Grade 10	Housewife			
			30113	37	Female		Housewife			
	11	Public	11114	40	Female		Polio Worker			
			11115	40	Female	No formal education	Housewife			
	41	Public	41116	42	Male	Grade 10	Private job			
			41117	30	Female		Housewife			
	51	Public	51118	40	Female	Grade 12	Polio Worker			
**FGD 2**	50	Private	50101	36	Female	Grade 5	Housewife			
			50104	40	Female	Grade 10	Housewife			
			50105	45	Male	Grade 12	Gatekeeper			
	21	Public	21102	33	Female		Housewife			
			21109	43	Female		Housewife			
			21110	50	Male	Grade 10	Private job			
	31	Public	31103	35	Female	Grade 8	Housewife			
			31107	42	Female	Grade 12	Housewife			
			31108	45	Female		Housewife			
	41	Public	41106	42	Male	Grade 10	Private job			
**IDIs with Headteacher**										
**IDI 1**	50	Private	50004	65	Female	Grade 16	Headteacher	28	Not applicable	Not applicable
	30		30021	50	Female	Grade 16		28		
	11	Public	11016	58	Female	Grade 16		41		
	31		31019	50	Female	Grade 16		34		
	21		21010	57	Male	Grade 16		40		
**IDIs with Teacher**										
**IDI 2**	31	Public	31018	31	Female	Grade 16	Teacher	10	10	Physics and Mathematics
	11	Public	11013	36	Female	Grade 14		12	6 and 7	Biology and Social Studies
	21	Public	21008	36	Male	Grade 16		10	9	Biology
	21	Public	21009	56	Male	Grade 14		29	6	Islamic Studies

	11	Public	11014	25	Female	Grade 16		2	9 and 10	Biology and Chemistry
	11	Public	11015	39	Female	Grade 14		9	9 and 10	Mathematics, Islamic Studies, and biology
	31	Public	31017	31	Female	Grade 16		2	6	Mathematics, Biology, and Social Studies
	50	Private	50007	19	Female	Grade 12		<1	10	Physics and Chemistry
	30	Private	50006	32	Female	Grade 14		4	9	Biology
	50	Private	50005	34	Female	Grade 14		3	6	Social Studies
	30	Private	30022	29	Female	Grade 16		3	9	Physics, Chemistry, Biology and International Primary Curriculum
	30	Private	30023	33	Female	Grade 14		10	6	Mathematics and International Primary Curriculum
	30	Private	30024	34	Female	Grade 14		9	10	Biology and Chemistry
**IDIs With Canteen Staff**										
**IDI 3**	51	Public	51025	70	Male	No formal education	Canteen staff	58	Not applicable	Not applicable
	31	Public	31020	57	Male	No formal education		35		
	21	Public	21011	42	Male	Grade 12		1.5		
