# Supplementary material for: Youth-led co-creation of a non-communicable disease risk reduction intervention in urban informal settlements of Karachi, Pakistan: a participatory action research
Source: BMJ Public Health. 2026 Nov 5;4(4):e005286. doi: 10.1136/bmjph-2026-005286 (PMC13756321; doi:10.1136/bmjph-2026-005286)
Supplement: Supplementary file 3 [file bmjph-4-4-s003.pdf]

Table S3: YHH voting for different aspects of the Intervention

Different aspects of the Intervention	Workshop Day 1	YHH Votes (N=31)
Intervention activities	Role-play	28
	Physical Activity	31
	Animated Video	30
	Storytelling	11
	Charts	11
	PowerPoint Presentation	20
Intervention session duration	45-min	24
	30-min	11
Intervention delivery time frame	Once-a-week	16
	Within-a-week	24
Number of sessions	1	9
	2	24
	**Workshop Day 2**	**N= 23**
Role-play title	Sehat ka Safar, Saath Mil Kar	6
	Jahan Sath Wahan Sehat	23
	Dosti se sehat tak ka safar	21
Selection of physical activities	Heads, Shoulders, Knees and Toes	19
	Simon Says	19
	Hand Clap	7
	High Knee	15
	Arm Circle	18
Animated video title	YHH - k sath sehat ka safar	2
	YHH - Rooshan Kal ki Umeed	20
	YHH - Sehat kay Rakhwalay	16

YHH= Youth Health Heroes

* Youth were allowed to vote for multiple options
