# Supplementary material for: Youth-led co-creation of a non-communicable disease risk reduction intervention in urban informal settlements of Karachi, Pakistan: a participatory action research
Source: BMJ Public Health. 2026 Nov 5;4(4):e005286. doi: 10.1136/bmjph-2026-005286 (PMC13756321; doi:10.1136/bmjph-2026-005286)
Supplement: Supplementary file 5 [file bmjph-4-4-s005.pdf]

Focus Group Discussion (FGD) with Youth

BEFORE THE INTERVIEW/DISCUSSION STARTS

- Thank the participant(s) for their time and contribution.
- Explain that the interview/FGD will take approximately 45-60 minutes
- Ask them if they have any questions about the interview and answer them.
- Complete and sign the assent and parental consent form (2 copies, participant keeps one, researcher keeps one).
- Complete the Demographics Information Form.
- We would like to know what they think about suitable program for youth in understanding shared risk factors of noncommunicable diseases (NCDs) and promoting prevention among their peers.
- The interview/FGD will be digitally audio-recorded. Explain why we use the recorder by telling the participants we can talk to each other directly without the researcher having to write it down and more accurately record what they say. (If they refuse to record the interview/FGD, you will need to take notes.)
- Reassure them that there are no right or wrong answers; we are interested in their experiences and views so that we can improve the study, so please be honest. Participation is anonymous, and they will not be identified.
- Tell them they can choose not to answer any questions if they feel uncomfortable or prefer not to disclose certain information.
- Tell them they can stop us at any time.
- Turn on the recorder and start the interview.

Participant Information:

Collect basic information, such as age and gender for context.

Part A: Introduction:

To start our discussion, may I request everyone briefly introduce yourself?

Part B: Discuss the findings of the Phase I study:

Before we proceed, I want to share some findings of our Phase I study, i.e., the shared risk factors of NCDs among school-going children in Azam Basti.

Part C: Ask and then Introduce the topic:

1. Can you share what you understand about NCDs and how did you learn about them?

2. Have you seen people in your family who experience NCDs (in your immediate family, close relatives)? Can you name some NCDs?

3. Do you know what are some causes of NCDs?

Great! During this interview, we will discuss some noncommunicable diseases (NCDs) like diabetes, heart disease, high blood pressure, and cancers and the shared risk factors of these diseases, such as unhealthy diet, physical inactivity, and tobacco use.

4. Can you share your thoughts on the importance of educating youth about the shared risk factors of noncommunicable diseases?

5. In your opinion, why is it valuable for youth to take an active role in promoting NCD prevention among younger peers?

Part D: Existing Materials Assessment:

6. Can you think of any materials related to NCD prevention that you've found effective? If yes, can we discuss the components of that material?

Part E: Preferred Learning Materials:

7. What types of intervention materials do you think would be most engaging and effective for youth and younger peers?

8. Are there specific formats (storytelling, videos, infographics, pamphlets, interactive sessions, games, etc.) that you believe would help youth and younger peers understand the topic and absorb as much information as possible?

9. What do you think the intervention should include different modules covering different aspects of learning strategies like interactive session, followed by storytelling, a video clip, and games, such as word search?

10. Do you think real-life examples should be incorporated into the material to make it more relatable to younger peers?

11. To what extent should technology be integrated into the intervention material, and what types of technology would be most effective considering the facilities available in the schools?

12. How can we ensure that the intervention material is directly relevant to the daily lives of younger peers?

Part F: Other aspects of the intervention:

13. What language do you think would be most effective for conveying information?

14. In your opinion, what would be the ideal number of sessions for the intervention to ensure comprehensive understanding among younger peers?

15. In how many days should the intervention sessions be delivered completely to younger peers? (e.g., within a week, the response for this question is depending on the participant's responses for the number of intervention sessions)

16. How long should each intervention session last to ensure maximum engagement and understanding among younger peers?

Part G: Empowerment and Motivation:

17. How can we actively involve youth in developing and delivering the intervention to ensure it aligns with their interests?

18. How can the intervention empower youth to take ownership and become advocates for preventive health measures for NCD prevention in their communities?

19. What motivational strategies do you believe would be effective in encouraging youth to actively participate in and commit to the intervention delivery?

20. Are there potential barriers that might deter youth from participating, and how can these be addressed to enhance engagement?

Final Thoughts:

Are there any additional suggestions or considerations you would like to share regarding the intervention material?
