# Supplementary material for: Youth-led co-creation of a non-communicable disease risk reduction intervention in urban informal settlements of Karachi, Pakistan: a participatory action research
Source: BMJ Public Health. 2026 Nov 5;4(4):e005286. doi: 10.1136/bmjph-2026-005286 (PMC13756321; doi:10.1136/bmjph-2026-005286)
Supplement: Supplementary file 6 [file bmjph-4-4-s006.pdf]

Study title: Co-creating a Youth-led Non-Communicable Diseases Risk Reduction Intervention with School-going Adolescents in the Slums of Karachi, Pakistan

Training Manual

A Guide for Youth Health Heroes for NCD Risk Reduction Intervention

YOUTH HEALTH HEROES

NCDs Risk Reduction Initiative

Acknowledgements

We extend our heartfelt gratitude to all those who contributed to the development of this manual. Special thanks to our study team, the school leadership, staff, youth, and parents, whose insights and experiences helped shape the content. We acknowledge the dedicated efforts of all Youth Health Heroes who contributed to the development of the intervention. Special thanks to the Aga Khan University (AKU) and our study funders the Royal Society of Tropical Medicine & Hygiene (RSTMH) and the National Institute of Health and Care Research (NIHR) for their support.

Table of Contents

1. Introduction....1

Day 1 .... **2-21**

2. Animated video 3-12
   - 2.1. Introduction of Youth Health Heroes 4
   - 2.2. Introduction of NCDs and their risk factors.... 5-6
   - 2.3. Show the video 6-10
   - 2.4. Post video reflections 10-12
3. Physical activity 13-21
   - 3.1. Introduction to the activity 14
   - 3.2. Head, Shoulders, Knees and toes 14-16
   - 3.3. Simon says 16-17
   - 3.4. Arm circles 17-18
   - 3.5. Benefits of physical activity 19-21

Day 2.... **22-42**

4. Role play 22-44
5. Annexures.... 45-59
   - 5.1. Younger's peer anthropometry....45
   - 5.2. Youth FGDs 46
   - 5.3. Healthy snacks....47
   - 5.4. 1st Workshop 48
   - 5.5. 1st Workshop sessions 49
   - 5.6. 1st Workshop group activities....50
   - 5.7. 2nd Workshop....51
   - 5.8. 2nd Workshop-healthy food & badges 52
   - 5.9. 2nd Workshop activities 53
   - 5.10. Training of youth health heroes....54
   - 5.11. Intervention delivery 55
   - 5.12. Souvenirs for youth health heroes....56
   - 5.13. Certificate for youth health heroes....57
   - 5.14. Certificate for schoolteachers....58
   - 5.15. Certificate for schools....59

1. Introduction

Non-communicable diseases or NCDs, including diabetes, cardiovascular diseases (CVDs), cancers, and chronic respiratory diseases are the leading causes of morbidities and pre-mature in Pakistan. These diseases are often linked to modifiable lifestyle risk factors, including unhealthy diet, physical inactivity, and tobacco use(1). These behaviors often begin during childhood and adolescence and persist into adulthood (2).

Studies highlights that children and adolescents often lack awareness regarding the consequences of such habits and early interventions are required to mitigate these risks and thus preventing the onset of NCDs later in life(2).

This manual is developed as a guide for youth to implement NCD risk reduction intervention among younger peers in schools. The intervention components part of this manual was developed with the help of youth and their teachers after comprehensive qualitative interviews and workshops. It helps youth develop leadership skills and health advocacy to become ambassadors of change.

References

1. Jafar TH, Haaland BA, Rahman A, Razzak JA, Bilger M, Naghavi M, et al. Non-communicable diseases and injuries in Pakistan: strategic priorities. The Lancet. 2013;381(9885):2281-90.
2. Changoh CM, Tatah L, Aroke D, Nsagha D, Choukem S-P. Noncommunicable diseases behavioural risk factors among secondary school adolescents in Urban Cameroon. BMC Public Health. 2024;24(1):377.

Day 1

2. Animated video (Activity#1)

Allocated Time (mins)

25

Learning Outcomes

By the end of the session, the younger peers will be able to:

a) Understand NCDs and their shared risk factors
b) Relate the real-life scenarios shown in the video to their own lives
c) Assess and reflect on their lifestyle choices, such as unhealthy dietary habits, physical inactivity, and tobacco use

Instructions for Youth Health Heroes (YHHs)

1. The script or spoken dialogue is written in normal font.
2. Any instructions, questions to ask participants, or actions to take are in **bold with highlight**.
3. Youth health heroes should write their names next to the activity component that they want to lead.

Activities	Duration (mins)
2.1 **Introductions of Youth Health Heroes (YHHs)**<br><br>**(Start with a smile and greet the younger peers)**<br><br>YHH1: “Assalamu Alaikum, friends! “How are you all doing?<br><br>**(Wait for a response and react positively)**<br><br>YHH1**:** “MashaAllah, glad to hear that!”<br>YHH1: “You probably already know us. We are students of grades 9 and 10 from your school and also your Youth Health Heroes. **(**Pause**)** As Youth Health Heroes, our role is to raise awareness about different diseases and to educate you about healthy and unhealthy habits.”<br><br>**(Each YHH will introduce themselves one by one).**<br>**(After all 4 YHHs have introduced themselves ask the question according to the time of day)**<br>**If it is morning**<br><br>YHH1: **"**Have you all had breakfast?" (Wait for a response and say) "Breakfast is very important; it gives us energy for the whole day!"<br><br>**If it is afternoon or after lunch break**<br><br>YHH1: "Have you all had lunch?"<br><br>**(Wait for a response and say)**<br><br>YHH1: "Great, lunch keeps us strong and energized for the rest of the day!"	*Name:*<br><br>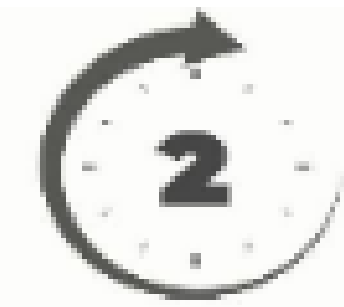

YHH1: "Now, I would like to invite my fellow facilitator to explain what are non-communicable diseases (NCDs) and what are their risk factors. Please give them your full attention!"

Look towards your fellow, and gesture for them to take over.

Name:

2.2 Introduction of the NCDs and their risk factors

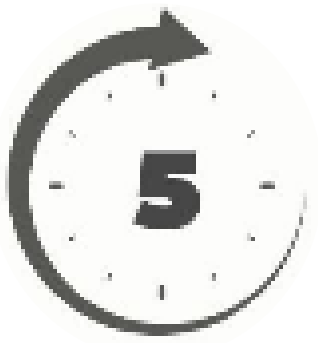

Speak with enthusiasm and engage the audience.

YHH2: "We often hear about diseases like diabetes, heart disease, lung disease, and cancer. Do you know why these diseases occur?"

(Pause and wait for responses.)

YHH: "Alright, dear friends! Today, we will share some very important information that is crucial for your health. Did you know that these diseases can be prevented?"

(Pause and observe the children's reactions.)

YHH2: "Yes! These diseases are called Non-Communicable Diseases (NCDs), and they usually develop due to unhealthy habits adopted in childhood, such as eating unhealthy food, not doing physical activities, spending too much time on mobile phones, TVs, and computers, and using tobacco products like cigarettes, gutka, and betel nut."

YHH2: But don’t worry! We have prepared an exciting**, animated** video for you that will explain all of this in a simpler way.

1. Before we start the video, we request everyone to sit quietly so that the sound is clear for everyone.
2. Watch the video carefully because we will ask you some questions afterward!
3. If you don’t understand anything, don’t worry! We will answer your questions at the end.

(Pause and wait for responses.)

"Let’s start the video!"
(In an enthusiastic tone)

2.3 Show the video: **https://youtu.be/Q_V21BoeFkY**

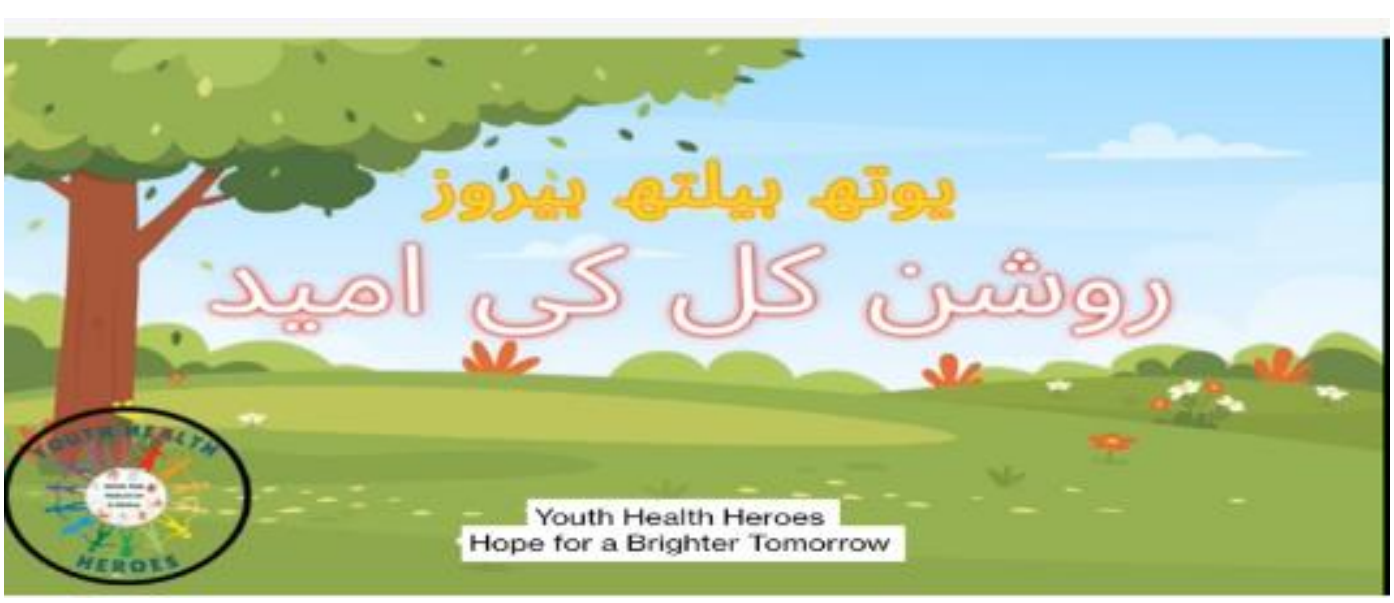

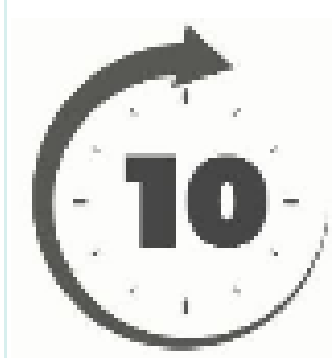

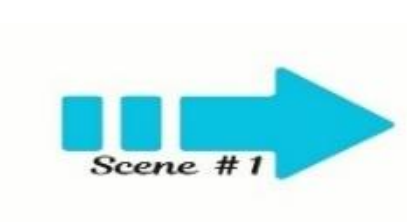

Heart Diseases Awareness

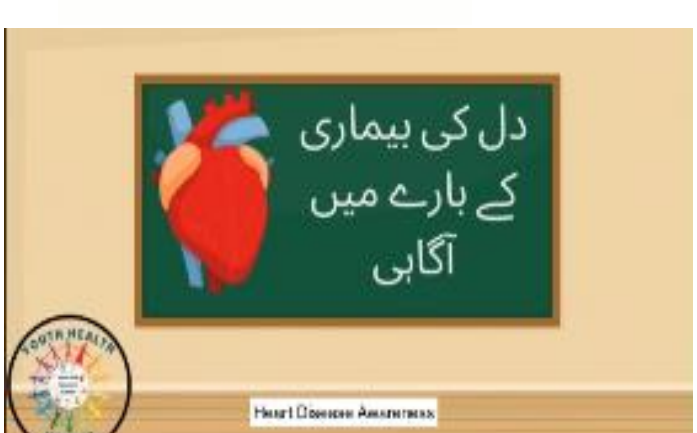

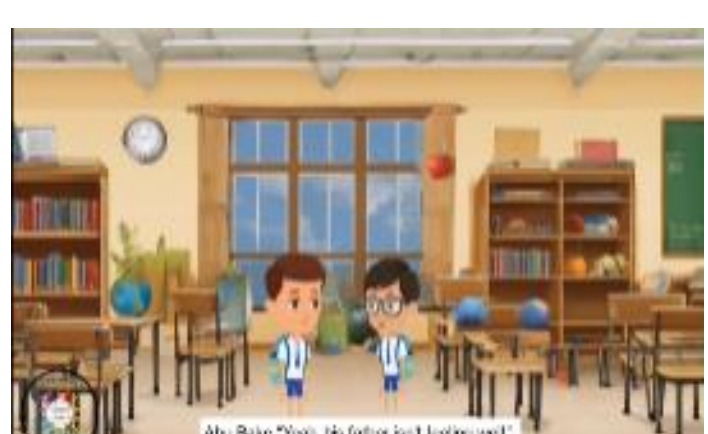

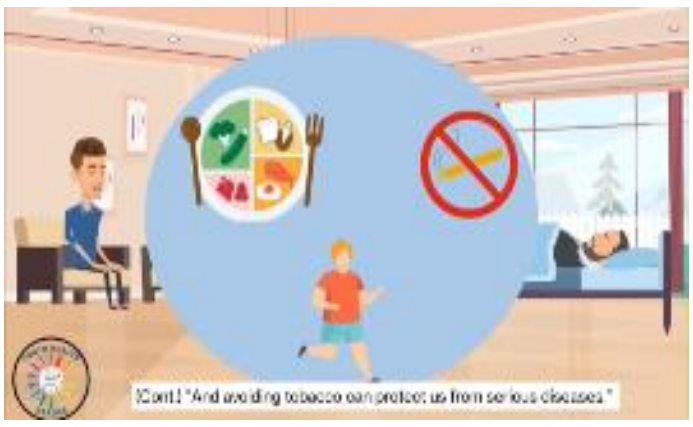

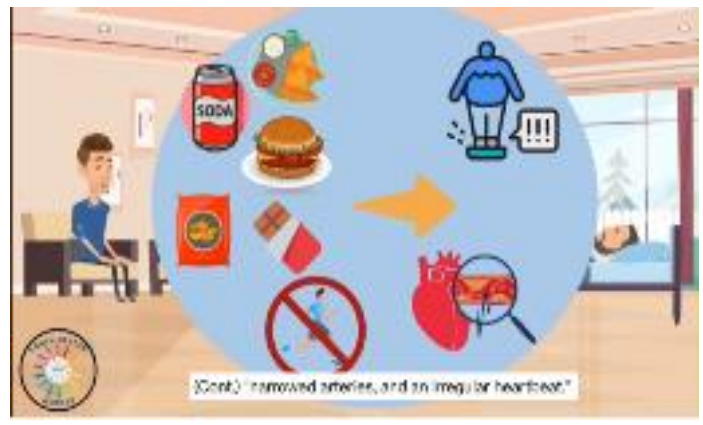

This scene is designed to teach students about the importance of adopting healthy habits, managing weight, and preventing heart disease. Here, the characters learn that heart disease is caused by poor lifestyle choices like unhealthy eating and lack of exercise. With guidance from a YHH, they understand how to prevent it and are encouraged to adopt good habits early and support others in spreading health awareness.

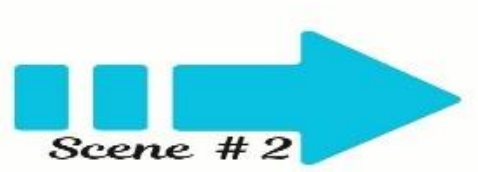

[Diabetes Awareness]

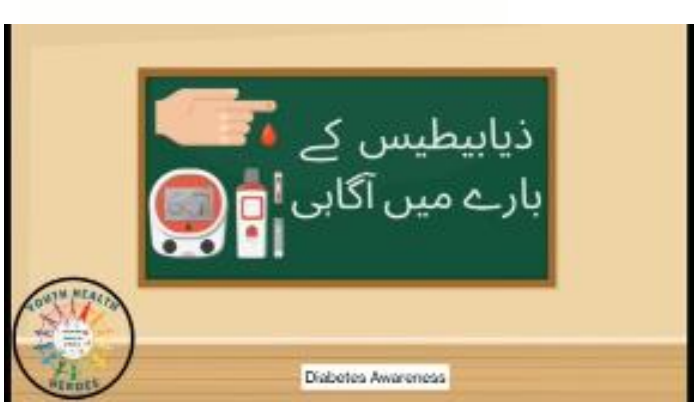

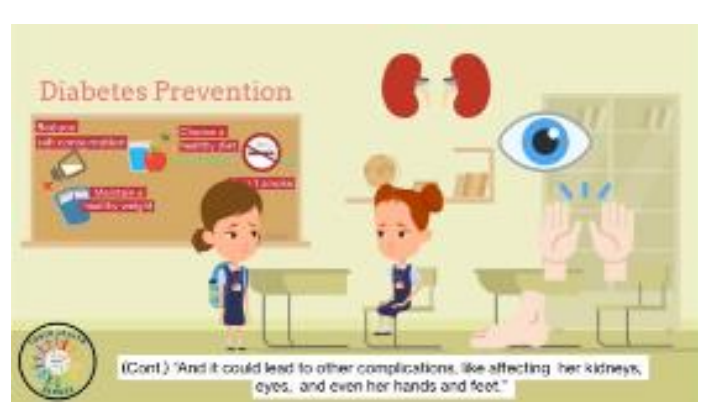

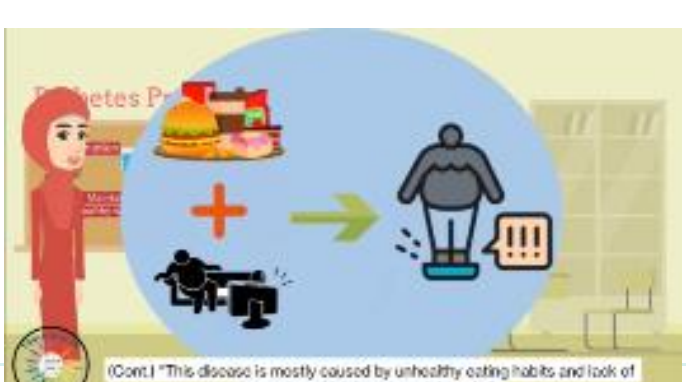

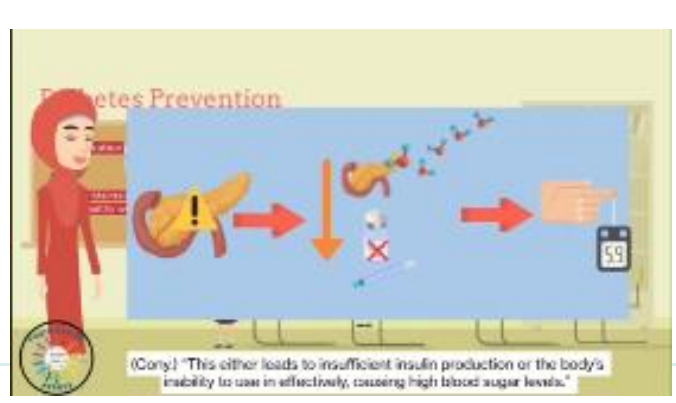

This scene teaches students about diabetes, its causes, and how it can be prevented or managed through healthy habits. The teacher explains that diabetes occurs when the body can’t produce or use insulin properly, often due to overweight and obesity. While it can't be fully cured, it can be controlled with healthy eating, and regular exercise. The story encourages children to adopt healthy lifestyle, such as healthy eating and regular physical activity to control weight and prevent the development of diabetes as well as support loved ones in managing health conditions through awareness and positive change.

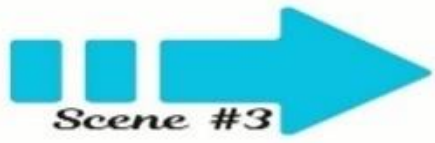

Cancer Awareness

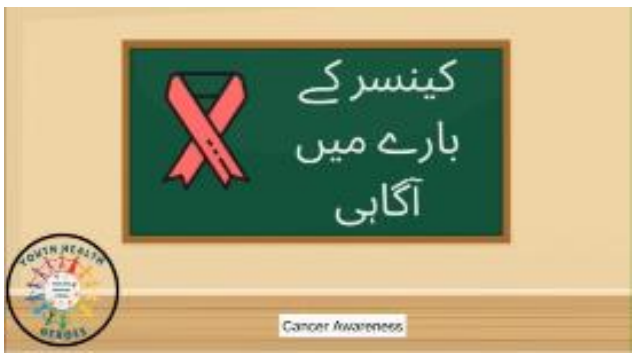

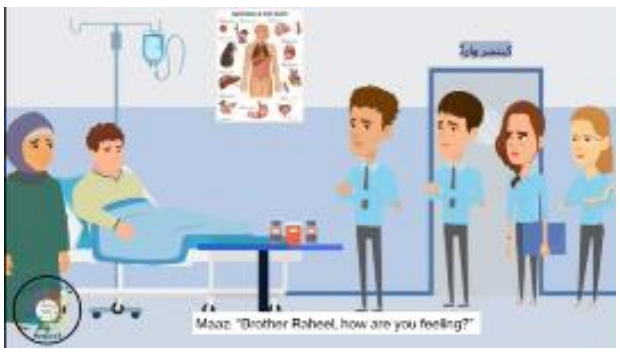

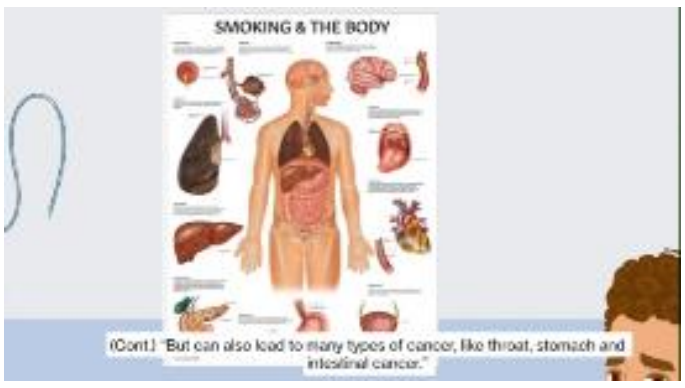

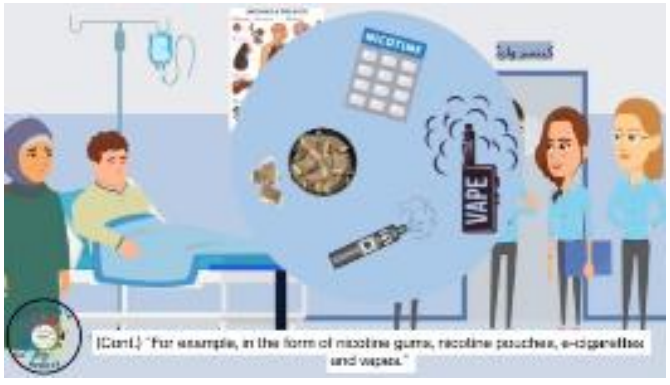

The message communicated through this story is that tobacco, gutka, betel nut, and modern nicotine products like vapes are dangerous and can lead to serious diseases like mouth cancer—even at a young age. It emphasizes that many youths fall into these habits thinking they are harmless or trendy, but the consequences can be life-threatening. The scene also promotes the idea that with awareness, support, and healthy choices, these risks can be prevented. It encourages young people to become Health Heroes by avoiding harmful substances, educating others, and valuing their health as a precious gift from Allah.

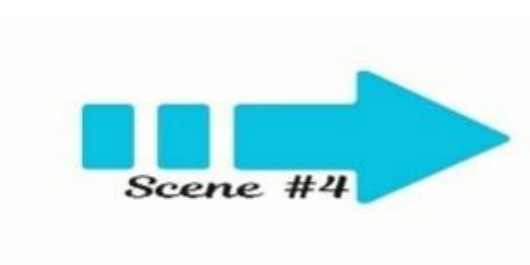

[Lung diseases Awareness]

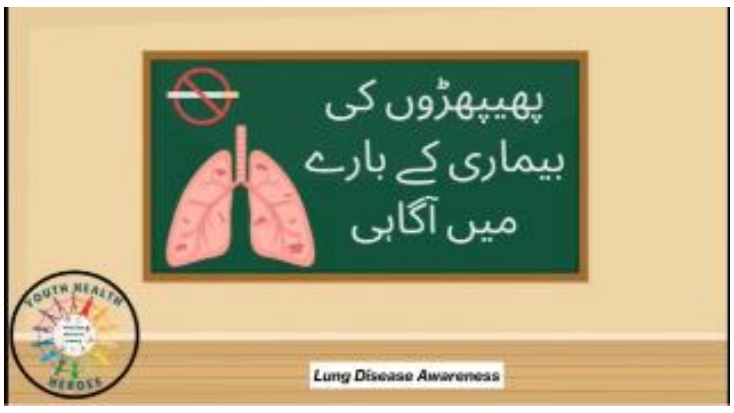

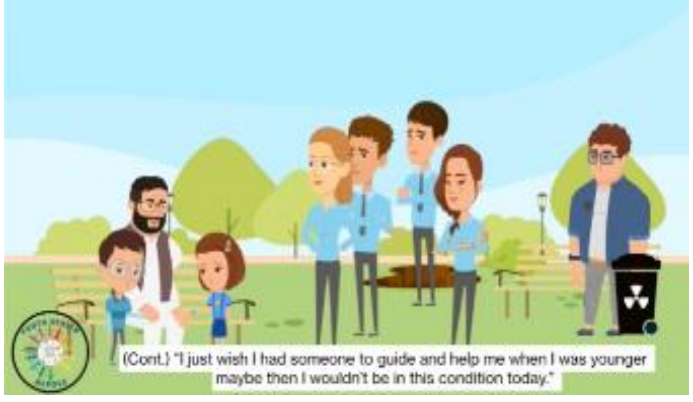

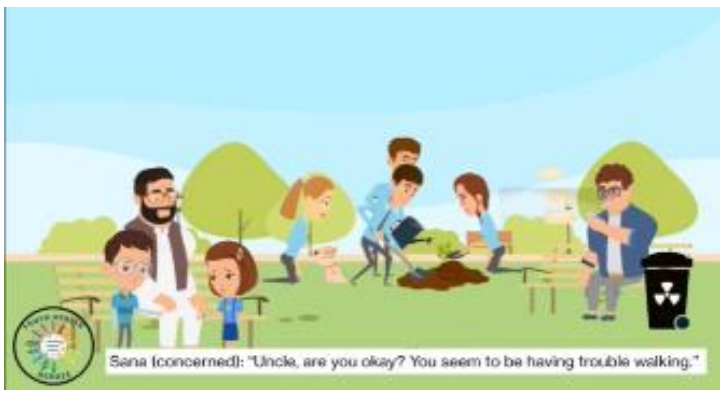

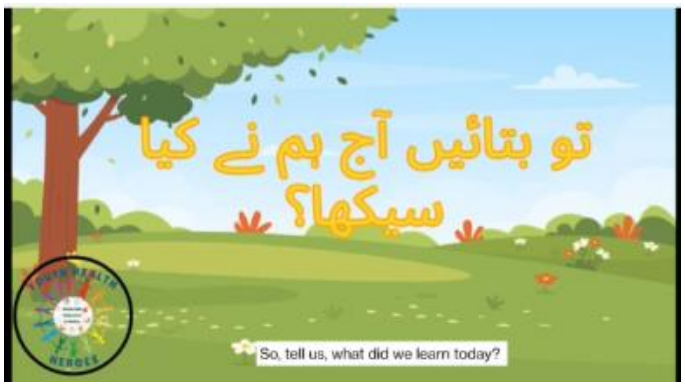

In the final scene of the video, the message communicated is that our everyday choices have a lasting impact on our health, and it's always encouraging to make a positive change. The older man, struggling with the consequences of smoking and an unhealthy lifestyle, expresses regret and wishes he had received guidance when he was younger. At the same time, a teenage boy begins to realize the risks he’s facing and is inspired by the YHH to reconsider his habits. This closing moment reinforces the idea that youth have the power to change unhealthy behaviors, protect future, and help others make healthier choices—proving that small actions today can lead to a healthier tomorrow.

Name:

2.4

Post video reflections

YHH 3 will ask 2 questions and YHH 4 will ask the other two

After listening to all the responses from the younger peers, the YHH responsible will also give the correct answers and take feedback after each question

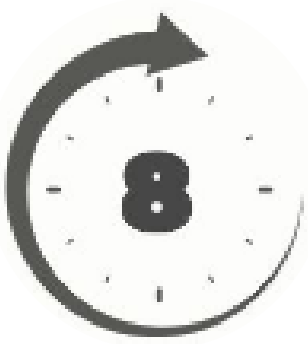

YHH3: “Friends! Did you Like the video? Do you have any questions? "

(After listening to the response)

YHH3: "Well, done. Now, we will ask some questions. Please raise your hand if you know the answer."<br><br>YHH3: Ques.1: What diseases did you learn about in this video? Mention their names.<br><br>**Answer: Heart diseases, diabetes, lung disease, and various types of cancer such as oral, throat, lung and colon cancer.**<br><br>YHH3: Ques.2: What are the causes of these complex diseases?<br><br>**Answer: Consuming unhealthy foods, lack of physical activity, excessive use of mobile phones, TV and computers, overweight/obesity and the use of tobacco products like cigarettes, gutka and betel nuts as well as secondhand smoke.**	
YHH4: Ques.3: How can we prevent these diseases?<br>**Answer:**<br>**By maintaining healthy body weight through**<br>• **adopting healthy eating habits, such as consuming more fruits and vegetables and avoiding unhealthy food that are high in fats and sugars.**<br>• **By doing regular physical activities like walking or exercising.**<br>• **By reducing screen time**<br>• **By avoiding smoking and other harmful substances like gutka and other nicotine products as well as betel nuts.**<br><br>YHH4: **Ques.:4: How will you convey the information from this video to others?**<br>**Answer:**	*Name:*

	• **I will educate my family members about the benefits of adopting a healthy lifestyle and preventing non-communicable diseases.**<br>• **I will create posters or charts highlighting the causes and prevention of diseases and display them in visible places at school or in the community.**<br>• **I will educate my friends, relatives, and neighbors about non-communicable diseases and their risk factors and how they prevent them.**<br>• **I will encourage and support those people in adopting healthier habits.**	

3. Physical Activity (Activity#2)

Allocated Time (mins)

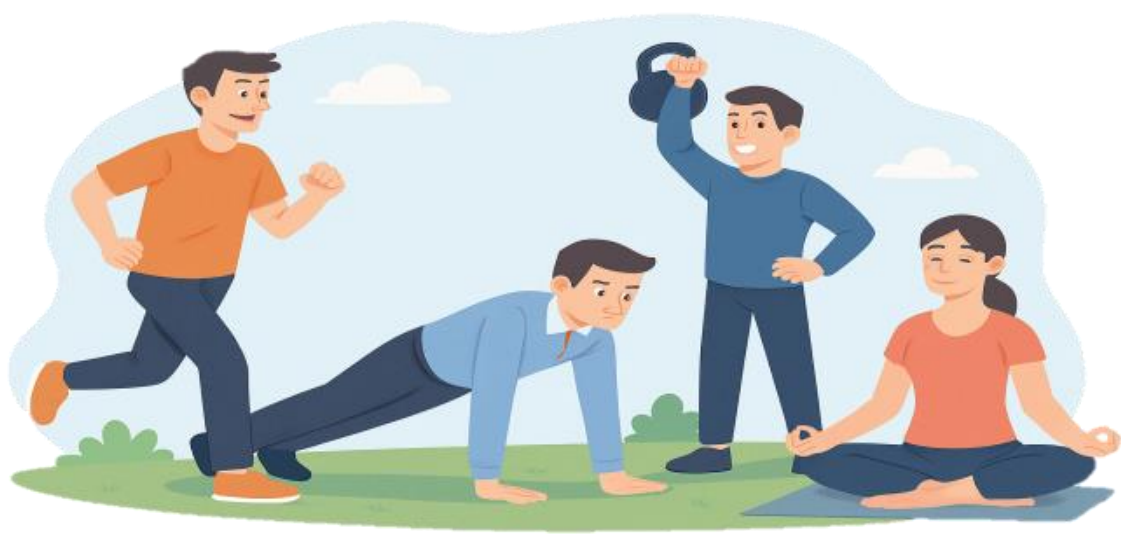

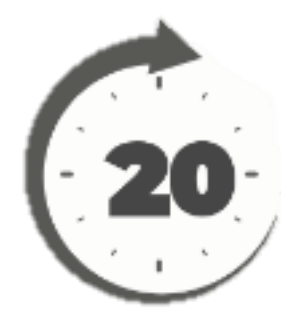

Learning Outcomes

By the end of the sessions, the younger peers will be able to.

a) Recognize physical inactivity as a risk factor for non-communicable diseases
b) Understand the benefits of physical activities
c) Learn classroom based physical activities

Instructions for Youth Health Heroes - (YHHs)

1. The script or spoken dialogue is written in normal font.
2. Any instructions, questions to ask participants, or actions to take are **highlighted**
3. Youth Health Heroes should write a name next to the activity component that they will lead.
4. Physical activities will first be demonstrated by the Youth Health Heroes.

Activities	Duration (mins)

3.1 **Introduction to the activity**

Name:

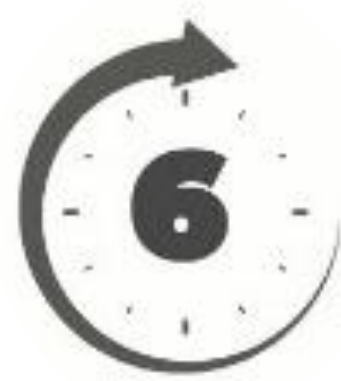

YHH1: "We have watched a video, now it is time to do some physical activity?"

In an energetic tone to get the younger peers' attention.

YHH1: "All students should sit at a distance so we can begin physical activity."

Pause and wait for responses.

YHH1: "We know that staying healthy requires a good diet, proper sleep, and regular exercise. Today, we will engage in some fun activities. In the end, we will ask you a few questions and share the benefits of physical activity with you. I invite my fellow Youth Health Hero.

Try walking around the room while saying this
Now, YHH 1 will invite YHH 2 to start the physical activity session.

3.2 **Head, shoulder, knees & toes**

Name:

YHH2: "Dear friends, I will now demonstrate a physical activity for you, and then we will all do it together. I need your full attention for this activity."

YHH: “The name of this activity is 'Head, Shoulders, Knees, and Toes.”

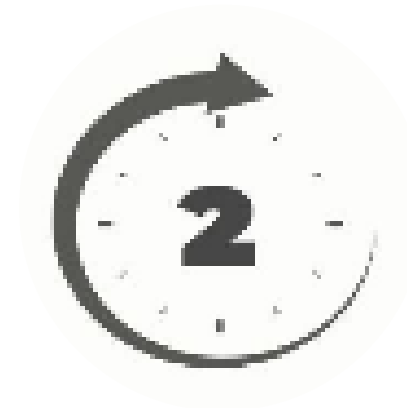

YHH2: “Now, everyone says the name of this physical activity together... Well, done!”

 “We will do this physical activity for 2 minutes.**”**

YHH 1 is responsible for ensuring that everyone is participating in the exercise and keeping track of the time.

“Great! Now, everyone stands up, and let's get started.".

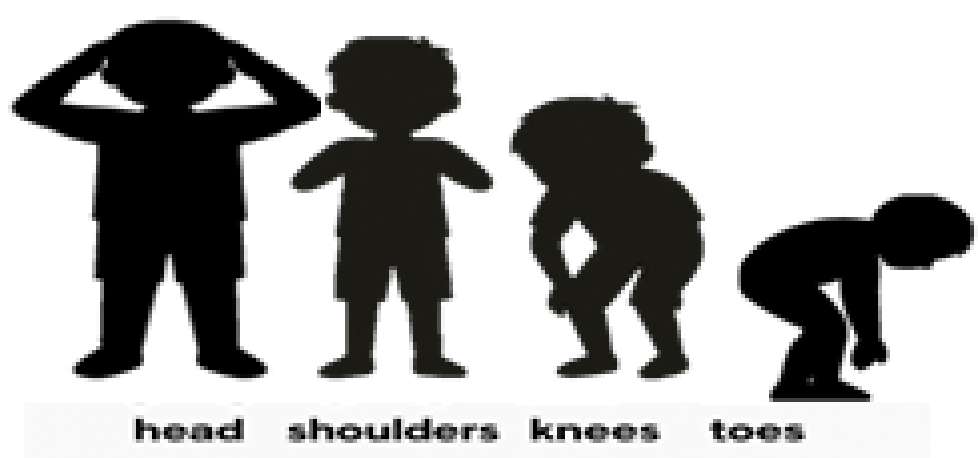

YHH 2will perform the activity twice.

The YHH 2 will lead the activity and guide everyone to follow along.

"Alright, friends, that's great! Please take your seats.”

YHH 1 will come back and call YHH 3.

YHH1: "Well done! Now, I would like to invite Youth Health Hero 3

3.3 Simon Says

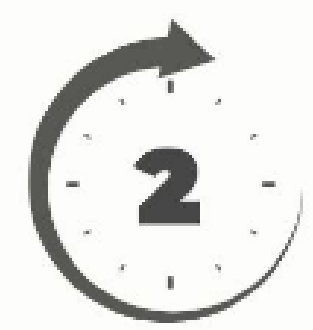

YHH3: "Now I will perform another physical activity, and then we will all do it together." (Pause)

"The name of our second activity is Simon Says. The Youth Health Hero will perform the activity".

"Now, repeat the activity name after me."

With a smiling face

"We will do this physical activity for 2 minutes. Alright, let's get started!"

The YHH 3 will demonstrate the activity and instruct everyone to continue following along

- Stand up
- Sit down
- Stand up
- Turn to the left
- Turn to the right
- Stand straight
- Raise your hands and clap
- Turn around
- Jump
- Sit down

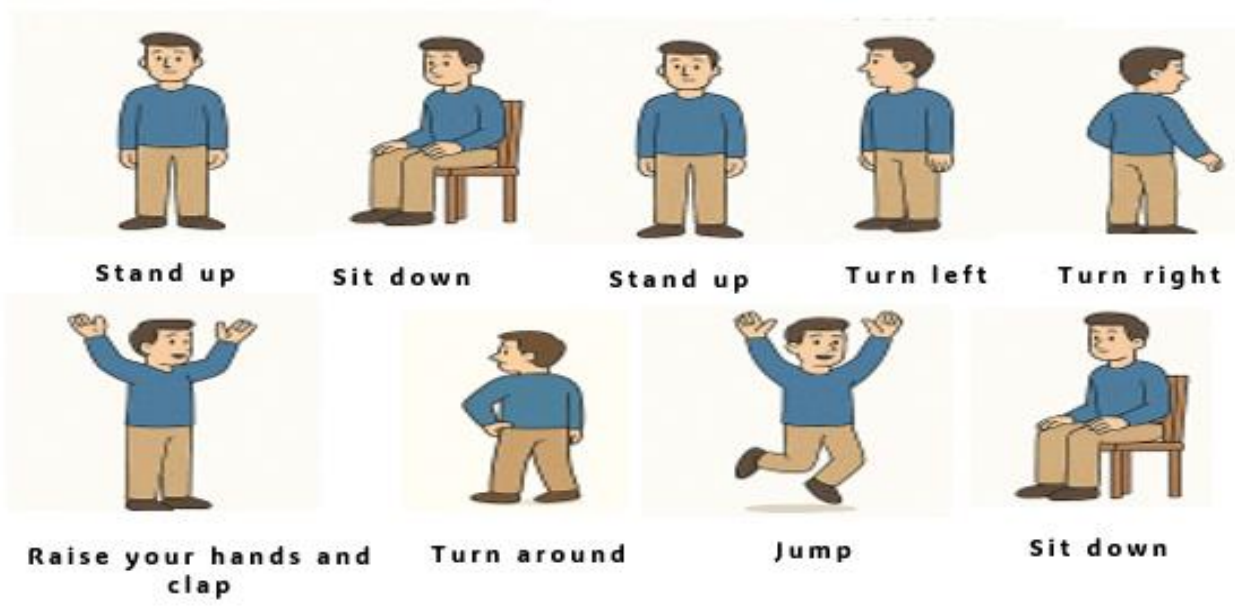

All these guides will be repeated twice.

YHH 1 is responsible for ensuring that everyone is participating in the activity and keeping track of the time.

YHH3: "Mashallah! You all did very well. Please sit down."

YHH 1 will come back and call YHH 3.

YHH1: "Very good! Now, I will request Youth Health Hero 4 **(say the name of YHH 4)** to demonstrate the third activity."

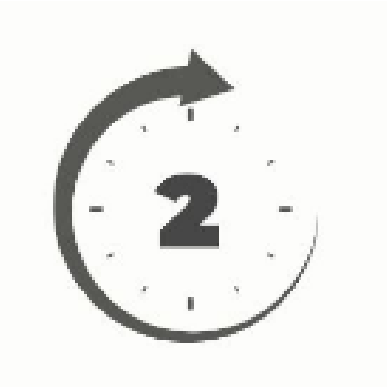

3 . 4 Arm Circle

YHH4: "Thank you!"

(With a smiling face, say)

YHH4: "Now, we will move on to the final activity, called Arm Circle."

"Now, all of you say the name of this physical activity together... **(wait for their response)** Well, done!"

YHH 4 will first perform the activity.

YHH 1 is responsible for ensuring that everyone is participating in the exercise and keeping track of the time.

"Alright, now let's start."

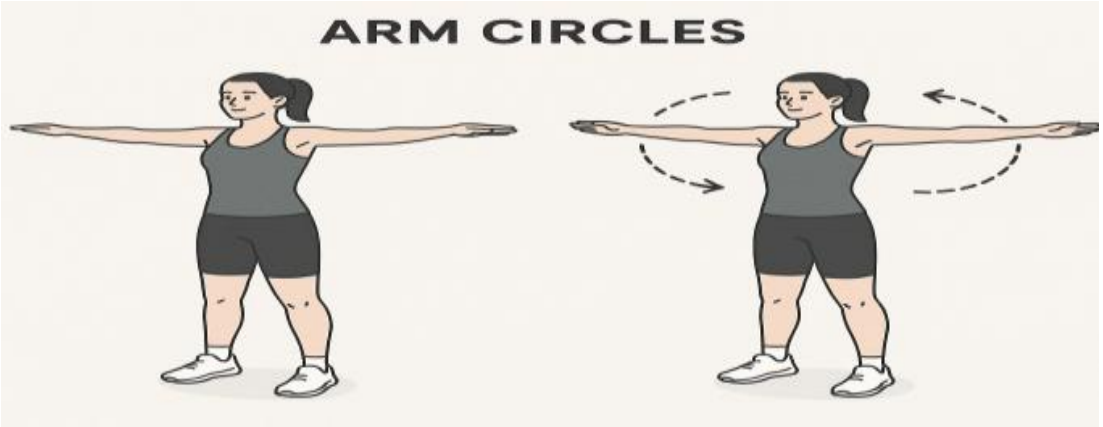

YHH 4 will demonstrate and instruct everyone to follow along.

"Extend both hands straight and move them in circles, starting small and gradually making them larger, first forward and then backward."

(After 2 minutes)

"Thank you! You all did very well. Please sit down."

"Do you know why physical activity is important?

(Pause and wait for answers)

"Alright, to discuss this, I will now invite Youth Health Hero **(say the name of YHH 1)** to come and discuss the benefits of physical activity and the disadvantages of not doing it."

3.5 Benefits of Physical activity

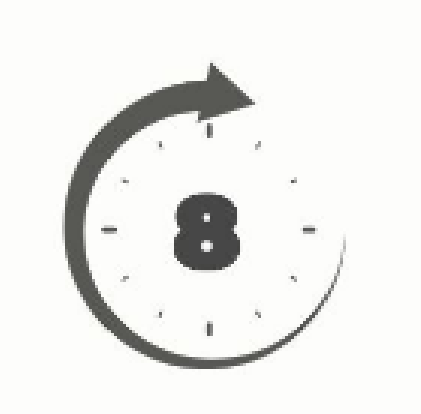

YHH1: "Friends, as my fellow Youth Health Hero 4 (**say the name of YHH 4)** mentioned, we will discuss the benefits of doing physical activity and the disadvantages of not doing it.
I hope you enjoyed all the phys*ical* activities we did today. Can anyone tell me why physical activity is important?"

After listening to all the answers...

"That's right! I have brought two posters to explain this. Let's discuss them."
(YHH 1 and 2 will show the two posters to all the children.)

"Look at this poster, which highlights different types of physical activities, like skipping rope, yoga, and even watering plants, which is also a form of physical activity."

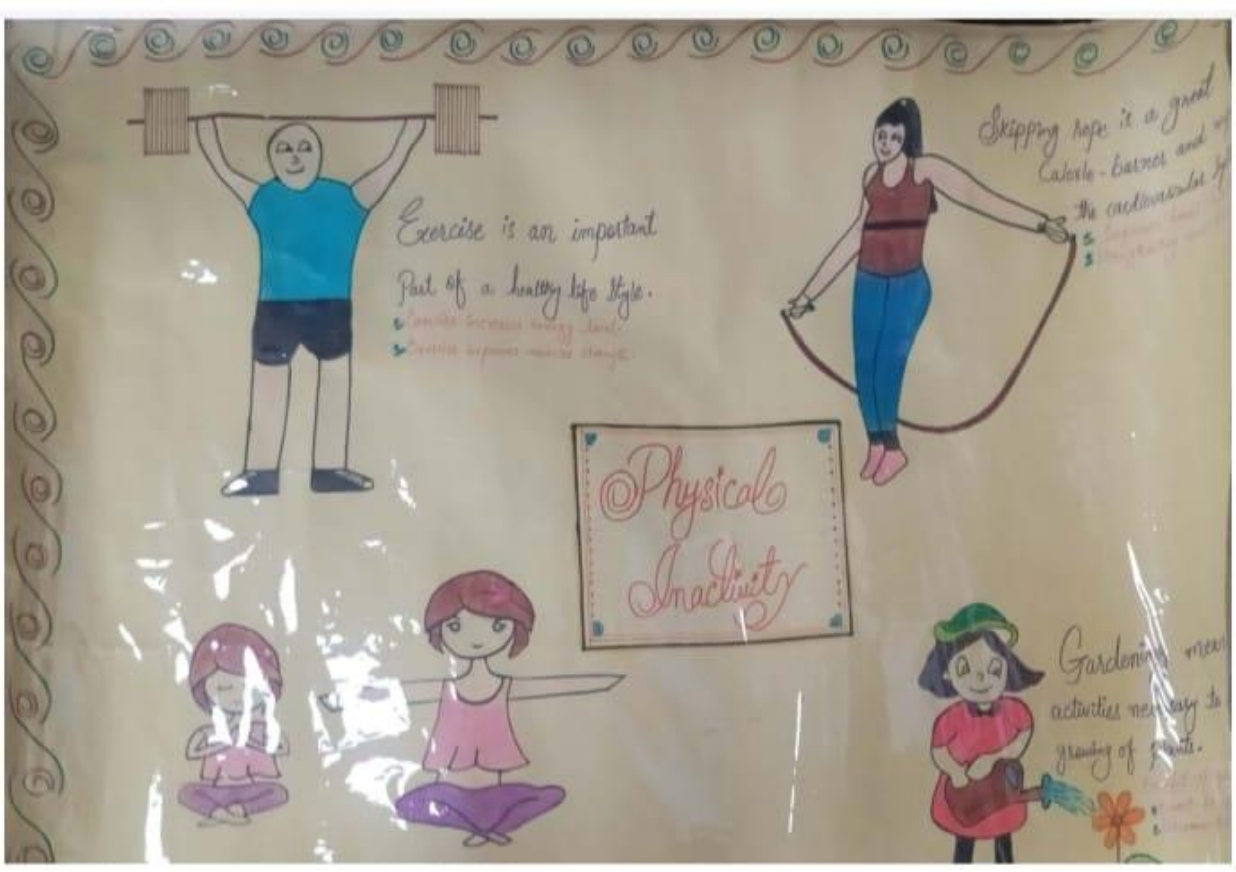

This poster will show that physical activity is not limited to structured exercises like gym workouts or sports. Everyday activities can also serve as valuable forms of physical activity that contribute to overall health and well-being.

(Make sure the second poster is within reach and smoothly transition between the posters)

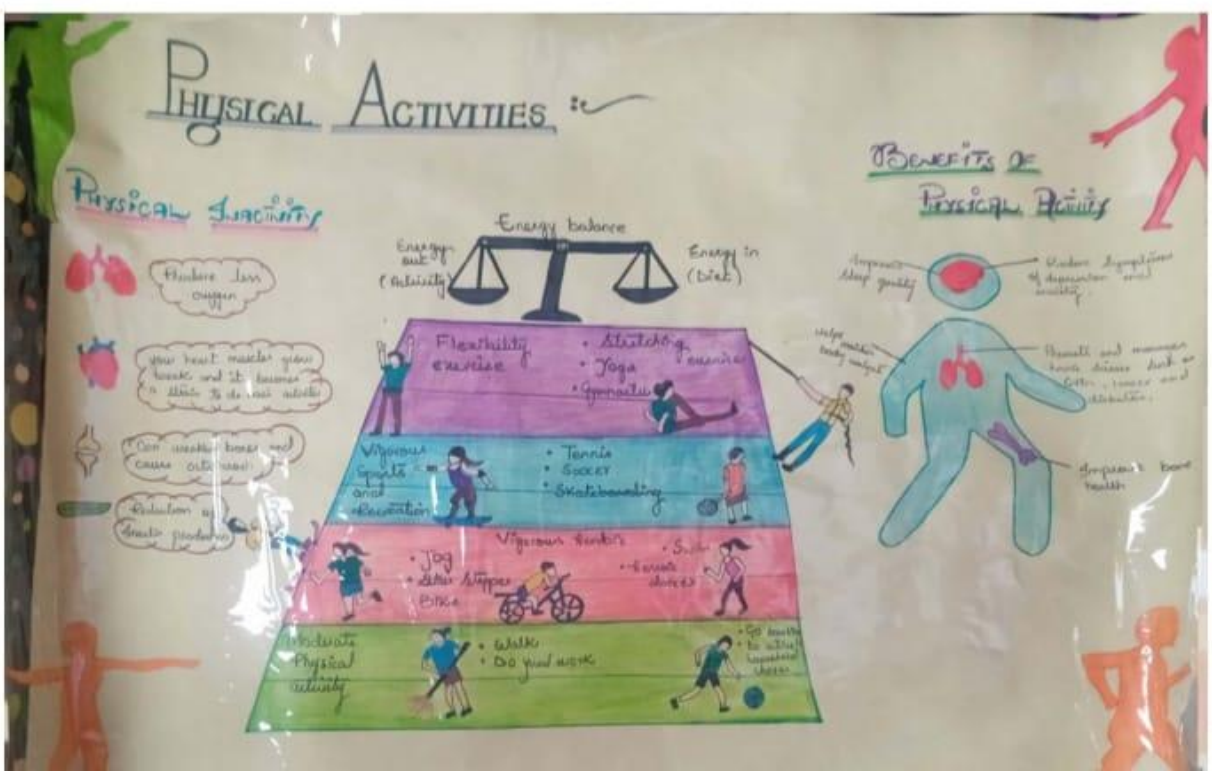

This poster will illustrate that any movement involving physical effort—whether structured exercise or daily tasks—is considered physical activity. The poster highlights its wide-ranging benefits, showing that regular movement not only supports heart health but also helps protect other organs from various diseases.

states
nt is
f it
are highlighted in the poster, showing that it not only keeps your heart healthy but also protects your other organs from many diseases.”

“In general, the benefits of physical activity are as follows:

- Physical activity helps maintain good health.
- It can protect us from complex diseases like diabetes, heart disease, and cancer.
- It helps in controlling weight.
- Physical activity aids in reducing stress."
- Physical activity helps keep the bones strong.

"We hope you all enjoyed it today, and I'm sure you're feeling great now.”

“So, will you make physical activity a regular part of your life?

Ask different questions relating to the activities performed and the video they were shown the remaining time left to ensure that the message being delivered was effectively conveyed.

(After responses)

“Well, done! (With enthusiasm)"Very well done! You all performed excellently. We hope you had fun. Thank you so much, and insha’Allah, we will meet again soon. Allah Hafiz.”

Day 2

4. Role-play (Activity#3)

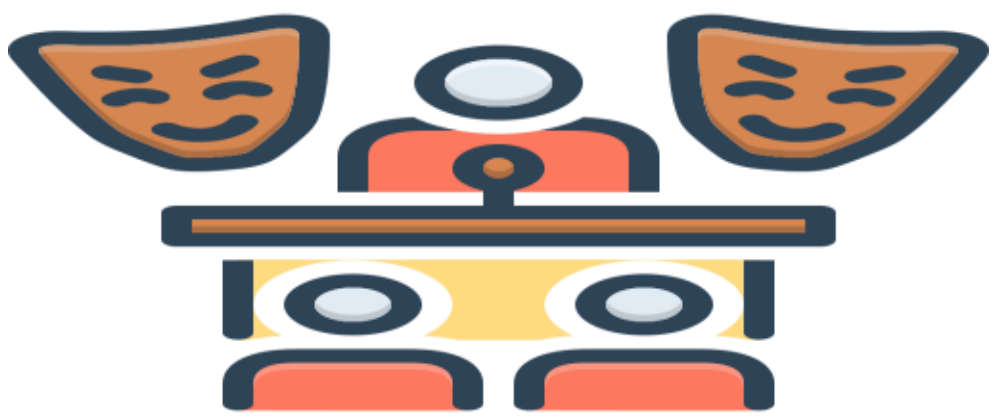

Allocated Time (mins)

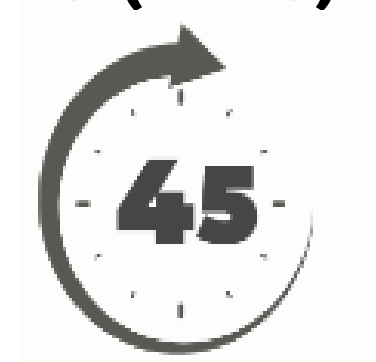

Learning Outcomes

By the end of the sessions, the younger peers will be able to.

a) Understand what are NCDs risk factors and how they develop
b) Recognize the importance of peer support in behavior change
c) Understand how healthy lifestyle can improve overall health and wellbeing

Instructions for Youth Health Heroes - (YHHs)

1. The script or spoken dialogue is written in normal font.
2. Any instructions, questions to ask participants, or actions to take will be written in **bold**.
3. The narrator's lines in the script have been underlined.
4. Youth Health Heroes have to make sure
   a. all props are used in the scenes
   b. change character name tags as per the scene
   c. After the scene ends, remove all the unnecessary props from the scene
5. Memorize your lines thoroughly.

Introduction

YHH1: Assalamualaikum! How are you all?

(After listening to the response) It's great to hear that you are all doing well.

"Do you remember what we did in the last session?"

(Pause and wait for a response)

“Exactly! We watched an animated video and did some exercises. Do you remember? Let's refresh our memory and see how much you recall!”

“Can anyone tell us what the video was about?”
(After listening to the responses) Well done. Today, we will present a drama (role play) titled 'Jahan Saath, Wahan Sehat (Where there is support, there is health).'

“We hope you all will watch it with full attention and understand its message so that you can gain complete knowledge.”
(With a friendly face)

- During the drama, no one will call out to anyone.
- All the participants will watch the drama in silence.
- No one will point at anyone.

Now, let's begin."

All Youth Health Heroes should write their names next to the character they want to play.

Characters
Ali=YHH 1
Ahmed=YHH 2; Father= YHH 2
Ayesha= YHH 3; Narrator= YHH 3
Habiba= YHH 4; Shopkeeper= YHH 4; Mother= YHH 4

Scene no 1

Characters
Narrator= YHH 3
Ali=YHH 1
Father= YHH 2
Mother= YHH 4

Scene props

- Desk (as study table)
- Cigarette (dummy)
- Mobile phone
- Pizza box
- Cake box
- Cold drink bottle
- Chairs

***Narrator 3*:** Ali is a bright kid. He always focuses on his studies and wants to score better in his exams. However, due to the environment around him, he gradually develops some bad habits, and his lifestyle starts becoming unhealthy. Today, we will present Ali's story to you.

In this scene, Ali will be shown sitting and studying at a table. Behind him, his father will be on the sofa, smoking a cigarette and using his mobile phone. Ali will glance back, notice his father smoking, but won't say anything and will return to his book.

Narrator 3 Ali aspired to be a perfect student in every way, and he was, but at home, certain factors were making it challenging for him. His father smoked cigarettes every day, which had a bad impact on his health. Ali's mother, a working woman, was often busy with her tasks at home as well.

Ali paused for a moment, then looked back at his father. However, he lowered his head again and focused on his books. He took a deep breath and tried to maintain his concentration.

Ali: **(thinking)** "I take care of my health, but with all this going on, what can I do?

Ali will shrug his shoulders

Ali’s mom enters the scene

Ali's mother: “Come on, Ali, today we’ve ordered pizza and cake for your brother's birthday.”

Ali’s mom will put the food in front of him

Ali (thinking): “My teacher always says fast food is unhealthy, but everyone at home eats it... (pause) Well, what's the harm in, having it? I haven't had anything fun to eat all day, so I might as well enjoy it now.”

Ali will pick up a slice of pizza, take a big bite, and quickly follow it with a sip of cold drink. A brief smile appears on his face, but then he pauses, lost in thought for a moment.

Narrator: Ali's thoughts reflect that this is not a good choice for his health.

Scene no 2

Characters
Ali=YHH 1
Father= YHH 2

Scene props

- Mobile phone
- Office bag
- Desk (table)
- Money
- Chairs

Ali is sitting in the living room, looking at his phone. His father, tired, enters the room.

Ali's father: "I'm really exhausted today.”

Show tiredness on his face and he will place his office bag on the side table.

Ali's father: “Ali, son, could you please get me some cigarettes? I’m out of them. Here’s the money. And if the shopkeeper refuses, just tell him that Raheel Sahab asked for them. He’ll give them to you. And you can keep the change."

Scene no 3

Characters
Shopkeeper= YHH 4
Ali=YHH 1
Narrator= YHH 3

Scene props

- Cigarette (dummy)
- Shop (written on a white paper)
- Money

When the scene ends, the character of the shopkeeper will quickly stick the shop tag on the wall and stand in front of it.

Then Ali will walk in, with money in his hand, and buy the pretend cigarettes from the shop. Once he has them, Ali will look at the cigarettes and analyze them.

Ali: “Dad smokes cigarettes every day. Maybe I should try it too, just once.”

Looking at the cigarette in his hand

“Let me try what cigarettes taste like.”

(Ali takes a puff and coughs)

“(coughing) What is this taste? It feels a bit strange...
(Ali coughs again) I won't smoke again.”

Scene no 4

Characters
Father= YHH 2
Ali=YHH 1

Scene props

- Mobile phone
- Chairs
- Ball

Next day: Ali's father is using his phone and asks Ali to bring him cigarettes.

Ali's father: Ali, son, please bring me some cigarettes.

(Ali was playing with a ball)

Ali: Okay, Dad.

Narrator: It became part of Ali's routine to bring cigarettes every day. As a result, he secretly started smoking, away from everyone's notice.

Scene no 5

Characters
Mother= YHH 4
Narrator= YHH 3
Ali=YHH 1

Scene props

- School bag
- Cup of tea
- Mobile
- Chairs

Ali:(**with a bag on his shoulder, returns home from school**). "Mom, today I'm going to the nursery………. My friends will come to pick me up soon. We're all going to the nursery for a group assignment on plants that the teacher gave us.

Ali's mother: **(holding a cup of tea in her hand, listens with slight concern.)** "There's no need for that. When all the information is available on Google, why go outside?"

Ali: **(in a convincing tone**) "but Mom, it's a group activity that we all need to do together. We have to observe the plants and then complete the assignment".

Ali's Mom: What's the big deal? You and your friends can divide the topics among yourselves, search for information, complete your parts, and let one of you combine everything.

Ali: **(in a low tone, sad)** "Alright, Mom, as you say. I'll just search for it and inform my friends as well."

Ali, convinced, starts using the computer and mobile.
"Wow! What an amazing game, Fortnite... It looks so much fun. Let me give it a try!"

Next Day: Ali comes home from school and starts playing games on his mobile.

Narrator: Ali began spending all his time after school engrossed in mobile games, gradually losing focus on his studies.

Scene no 6

Characters

Ali=YHH 1
Ahmed=YHH 2
Ayesha= YHH 3
Habiba= YHH 4

Scene props

- Ball
- Chips
- Soft drink
- Chairs

At school: During break time, Ali's friends look for him to play with the ball, but they find him looking tired, eating chips and drinking soft drink. The friends approach Ali together, holding the ball, and say

Ahmed: Ali, come on, let's play with the ball! It's always more fun when you're playing with us.

Habiba: **Speak encouragingly**
"Come on, let's play! It's been quite a long time since we played together!"

Ali: **(reluctantly)** "Guys, I really want to play, but I'm feeling a bit exhausted. I stayed up late last night. You all go ahead and play, I'll just watch."

The friends, while playing with the ball...

Habiba: **(with disappointment)** "What's happening to Ali day by day? He doesn't even play with us anymore, and the exams are coming up. He hasn't even completed his homework. "Ahmed: **(holding the ball)** Yeah, I've been noticing this change for some time now.

Scene no 7

Characters
Ali=YHH 1
Ahmed=YHH 2
Ayesha= YHH 3
Habiba= YHH 4

Scene props

- Exams File

Exams Scene, the friends will be shown holding the exam file in hand, walking slowly

Ayesha: **(asking Ali)** “how the exam was?”

Habiba: **(excitedly)** “Will you hit a six this time too?”

Ali: **(in a low tone, sad)** "It didn’t go well, guys. I don’t think I’ll make it this time."

Ayesha: **(worriedly)**"Why? What happened?"

Ali: “Honestly, I just can’t concentrate on studying these days. I constantly feel exhausted, and I’m not even getting enough sleep.”

Ahmed: **(reassuring)** “It’s okay, don’t worry. These are just the midterms — make sure to work hard for the finals.”

Ali: “Yeah, I just want to go home and sleep.”

Ahmed: “Alright, I’ll Walk with you. Okay then, see you later. Allah Hafiz.”

Scene no 8

Characters
Ali=YHH 1
Ahmed=YHH 2

Scene props

- Exam Files
- Cigarettes (dummy)
- (Shop) written on a white paper

After saying this, Ali and Ahmed start walking home slowly. On their way back, Ali and Ahmed are talking to each other.

Ahmed: Pakistan has a match today as well. I hope the team performs well.

Ali:(lost in his thoughts) Is the match today?

Ahmed: (surprised) Huh, don't you know?

Soon, they will reach the shop near which Ali buys cigarettes.

Ali to Ahmed: **(hurriedly)** "Alright, you go ahead, we'll meet later.

Ahmed will walk a few steps ahead and suddenly remember something and turns around and found Ali smoking. He will come back quickly, places his hand on Ali' shoulder, and says in surprise
Ahmed**: (shocked)** What’s this, Ali? You're smoking?

(Ali will panic, and the cigarette falls from his hand)

Ali: (**ashamed**) "Yeah Ahmad, I smoke sometimes... didn’t you go home?"
Ahmed: (**concerned**) "You know how harmful cigarettes are to your health, right?"

Ali: "Yeah, I know, but I smoke sometimes to relieve stress."

Ahmed: (**disappointing expressions)** "Okay Ali, see you at school tomorrow. “Ali: Allah Hafiz.

Scene no 9

Characters
Ahmed=YHH 2
Ayesha= YHH 3
Habiba= YHH 4
Narrator= YHH 3

Scene props

- Result sheet
- Chairs

Result day, a poster with results will be stuck on the front prior to the scene

Ayesha, Ahmed, and Habiba are sitting together. After looking at the result sheet hung on the wall together, they started talking.

They all seem sad seeing Ali has scored very low in the exams

Habiba: "Did you both see? Ali, who used to take positions every time, this time only passed with passing marks."

Ayesha: "Yeah, and Ali has changed a lot now. He doesn't engage in physical activities anymore, and even during lunch, he buys and eats unhealthy things.

Ahmed: "This is nothing, I've seen Ali smoking a cigarette..."
Habiba and Ayesha: Oh no!

Ahmed: "We should support Ali during this difficult time so that he can quit these bad habits and lead a better and healthier life."

Habiba: **(thinking)** Ahmad is absolutely right. We should work together to protect Ali from the complex diseases that could occur in the future, such as diabetes, heart disease, and cancer, which result from unhealthy eating, lack of physical activity, and smoking.

Habiba will look at both with questioning expressions.

Ayesha and Ahmed will nod their heads in yes

Habiba: For instance, participating in sports activities held at school is highly important, as such activities greatly contribute to both mental and physical well-being. I will take the responsibility of encouraging Ali to actively take part in them."

Ayesha **(smiling): "**Yes, of course. Ali is our friend, and taking care of his health is also our responsibility. I will look after his diet, try to keep him away from unhealthy food, and encourage him to eat healthy meals."

Ahmed (with enthusiasm):
"And I will make sure that Ali refrains from smoking and refocuses on his studies so that he can regain his position once again."

Ayesha & Habiba: "We will support him."

Ahmed: "And guide him back to a healthy lifestyle."

Habiba: **(suggestive tone)** "Why don't we all celebrate together tomorrow at school in honor of passing the exams? We can have a party and discuss both good and bad habits.

Ayesha **(with enthusiasm):** "Great idea, Habiba! I'll take responsibility for healthy food."

Habiba: "Alright then, I'll suggest some games that we can play."
Ahmed: "Okay, then I'll create a poster to illustrate good and bad habits."
Habiba: "Sounds perfect. Now let's go and prepare for tomorrow. Allah Hafiz!"

Ayesha and Ahmed: "Allah hafiz!"

Scene no 10

Characters
Ali=YHH 1
Ahmed=YHH 2
Ayesha= YHH 3
Habiba= YHH 4

Scene props

- Desk
- Snacks (fruits, yogurt, and egg sandwiches)
- Sports equipment (badminton and a ball)
- Posters
- Chairs

Ali is sitting at his desk, resting his head on it and Ahmed will enter the classroom

Ahmed**: (excitedly)** "Come on, Ali, today we've organized a party to celebrate passing the exams. We'll play lots of fun games, and Ayesha has brought some delicious snacks."

Ali: **(with sadness**) "Guys, I'm not in the mood. You all go ahead."
Ahmed: "Come on, buddy!" (Ahmed will grab Ali's hand) "Get up."

They will walk out of the scene and Ayesha and Habiba will enter the scene with snack props and sport equipment. Ali and Ahmed will re-enter the scene

Ahmed and Ali:"Assalamu Alaikum."

Habiba and Ayesha**:**"Wa Alaikum Assalam."

Habiba: "How are you both?"

Ahmed: "We're good."

Habiba: "Shall we play? I’ve brought badminton and a ball."
(Ayesha and Ahmed together):"Alright, let's go."

Ali: "Yes," (nodding his head)

(All the friends are playing together, 2 minutes later)

Ali: "I’m just tired now."

Habiba: **(smiling)** "Alright... let's eat something. What have you brought, Ayesha?"

Ayesha: "I’ve brought fruits, yogurt, and egg sandwiches for all of you today."

Habiba: **(smiling**)"Very good, Ayesha!"

All the friends will eat snacks together and soon Ahmed will start the conversation

Ahmed: "Guys, have you noticed that nowadays, Non-Communicable Diseases like heart disease, cancer, lung disease, and diabetes have become very common?"

Ayesha: "You’re right, Ahmed. At my home too, my parents often talk about these diseases.”

Ahmed: "And we know that these diseases are caused by unhealthy habits adopted in childhood. So, shouldn't we spread awareness about this?"

Habiba: "The idea is really good. But how are we going to do all of this?"

Ahmed: "Yes, in this regard, I have made some posters. We should start a campaign."

Ayesha: "That's a great idea, Ahmed. We should also give it a name."

All four will start thinking

Ahmed: "Health... Superheroes?"

Habiba: "Youth Health Heroes?"

Ayesha: **(with excitement)** "Wow, amazing, Habiba!"

Ahmed: "And look, friends, I've brought some posters too. We should start this campaign from our homes, school, and neighborhood."

Ayesha: "Great, Ahmed, show us your posters."

Ahmed: **(pointing to the food pyramid)**"Yes, here, take a look. Through this poster, we will highlight that we should eat a healthy and balanced diet."

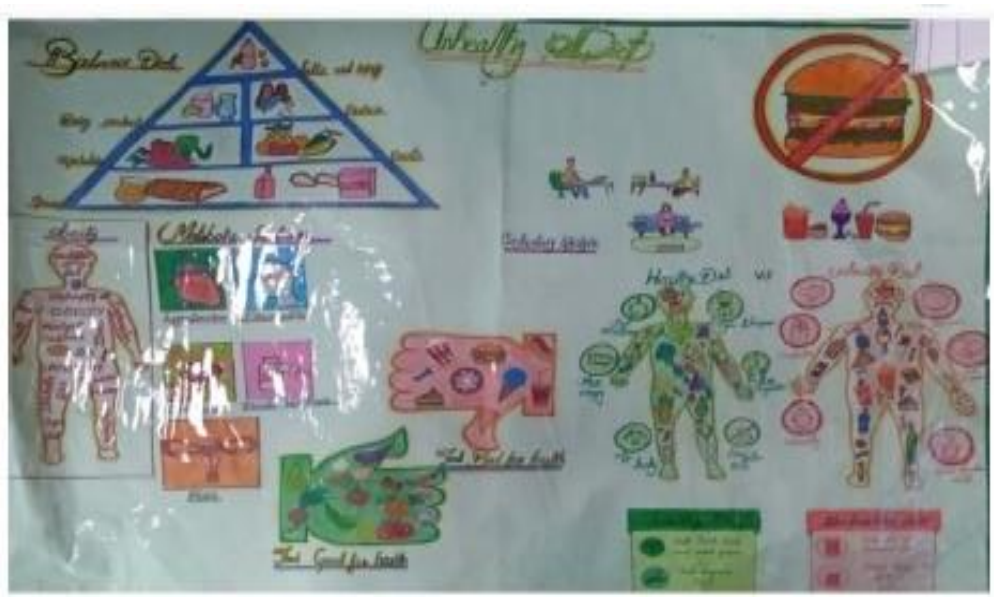

This poster is designed to discuss healthy and unhealthy food by the Youth Health Heroes."

"We should primarily focus on foods high in fiber, such as fruits, vegetables, and porridge. It's also important to choose whole wheat flour instead of white flour for roti, as white flour contributes to weight gain."

Ayesha: "Really?"
Ahmed: "Absolutely. After that, it's essential to include

vegetables and fruits in our diet, as they are excellent sources of vitamins and minerals. Additionally, we should incorporate milk and yogurt into our diet, because they help strengthen our bones. Protein intake, in the form of meat and eggs, is also important as it helps build our muscles. After all this, we should minimize the consumption of unhealthy foods like junk food, fried items, bakery items, food high in fat and sugar. We should also stay away from cold drinks and sugary beverages so we can remain healthy.

Habiba: **(pointing to the poster on physical activity)**

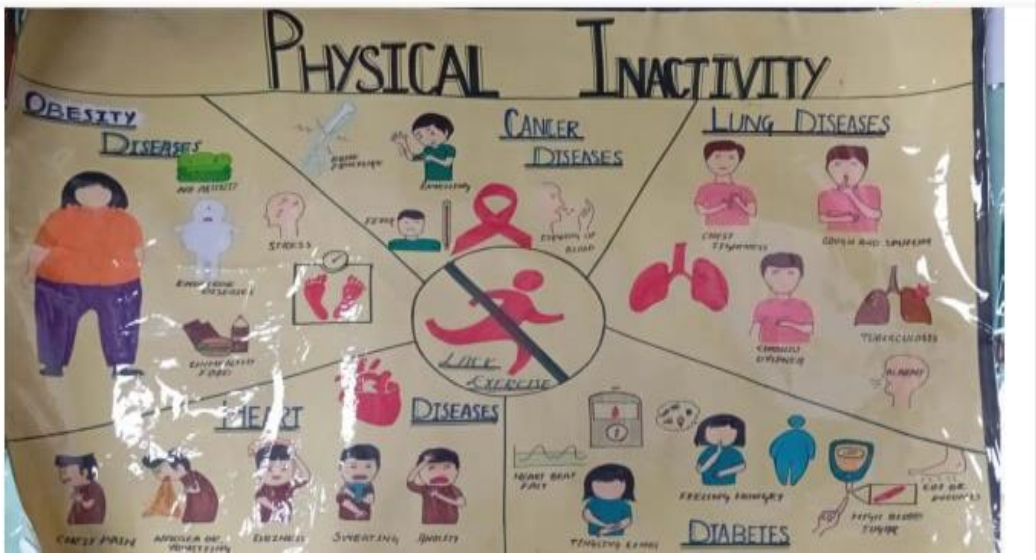

This poster aims to demonstrate the importance of regular physical activity for overall health, while also highlighting the growing concern around increased screen time and its effect on our health.

"Well done, Ahmed! ... And look at this. These days, we’ve all become so dependent on our mobile phones and TV screens. We often don’t realize how spending so much time on them can lead to various health issues like weight gain."

Ahmed: "Yes, Habiba, you know we should be doing physical activity for at least an hour every day to stay fit and avoid these chronic diseases. Another important point is that physical activity is essential for everyone, whether they are overweight or underweight, so we can all stay healthy."

Ayesha: **(pointing to the tobacco poster)**

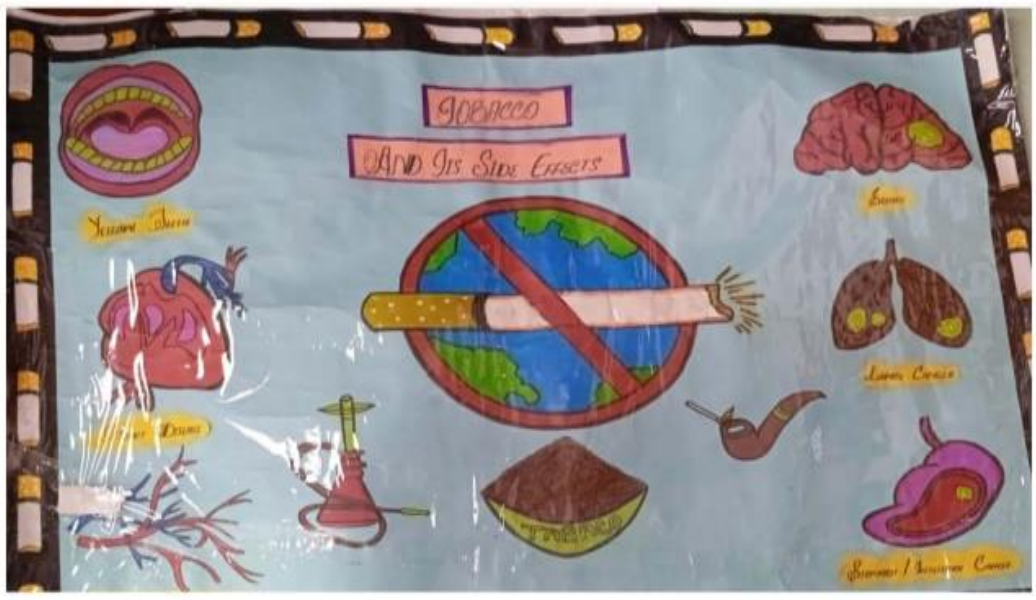

This poster will highlight the different forms of tobacco around us and its effect on our health.

Ayesha: "And what's this you've shown here, Ahmed?"

Ahmed: "Yes, take a look. Tobacco is found in many forms around us: cigarettes, gutka, naswar, hookah, shisha, and nowadays, there's the trend of e-cigarettes and vapes. All of these are extremely harmful. Once a person becomes addicted to them, quitting becomes incredibly difficult."

Ayesha: "Oh no! Is it available in so many different forms?"

Ahmed: "Yes, Ayesha, and people who use cigarettes or vapes are not only damaging their own health, but the smoke also puts those around them, including their loved ones, in danger."

Habiba: "Thank you so much, Ahmed! You've really enhanced our knowledge. These habits have become so common these days that we don't even think about the harm they cause. It's a great idea that we should raise awareness about it... Why are you silent, Ali? You should say something too."

Ali: "Friends, thank you so much. I've realized that I was on the wrong path, and I've now decided that not only will I stay away from all bad habits, but I will also spread this knowledge. I will start by promoting it in my own home."

Habiba: "Well done, Ali! You've made a great decision. Let's all make a promise that not only will we stay away from these habits, but we will also encourage our loved ones to do the same."

All four will turn to the students and say

Ali: "So, friends, are you all with us?"

(After hearing the response)

Ali: "Well done! Now, let's all take an oath. Please raise your right hand and repeat after us."

Wait for the students to raise their hands, and then each health hero will say their line.

Ahmed: "We promise that we will stay away from bad habits like smoking and teach others to do the same."

Ensure that all students, including the remaining 3 YHHs, are repeating after them

Habiba: "We promise that we will perform regularly physical activity and teach others to do the same."

Ensure that all students, including the remaining 3 YHHs, are repeating after them

Ayesha: "We promise that we will stay away from unhealthy foods and educate others about the harmful effects of these foods."
Ali:"Thank you all very much."

Annexures

Annex
Younger peer's anthropometry

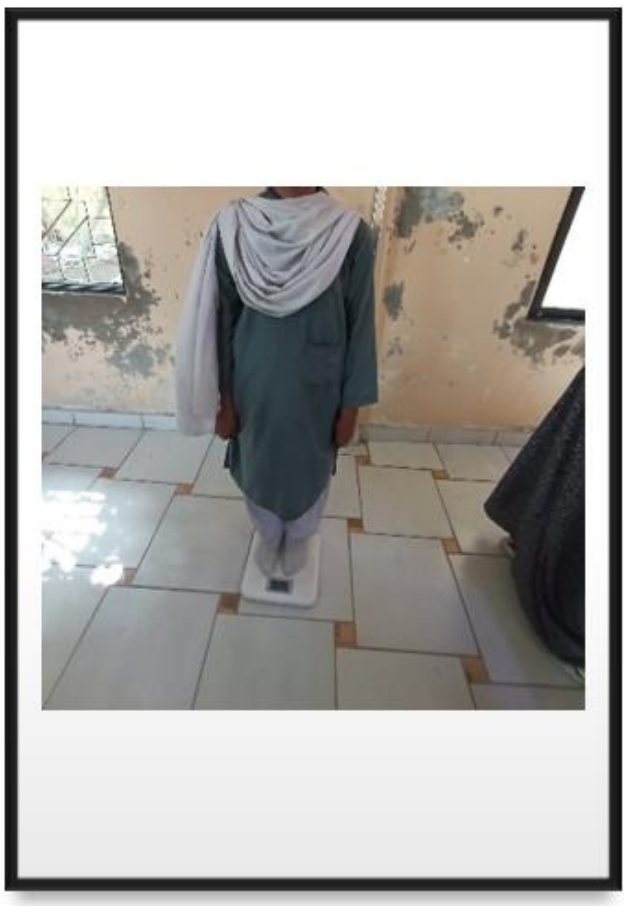
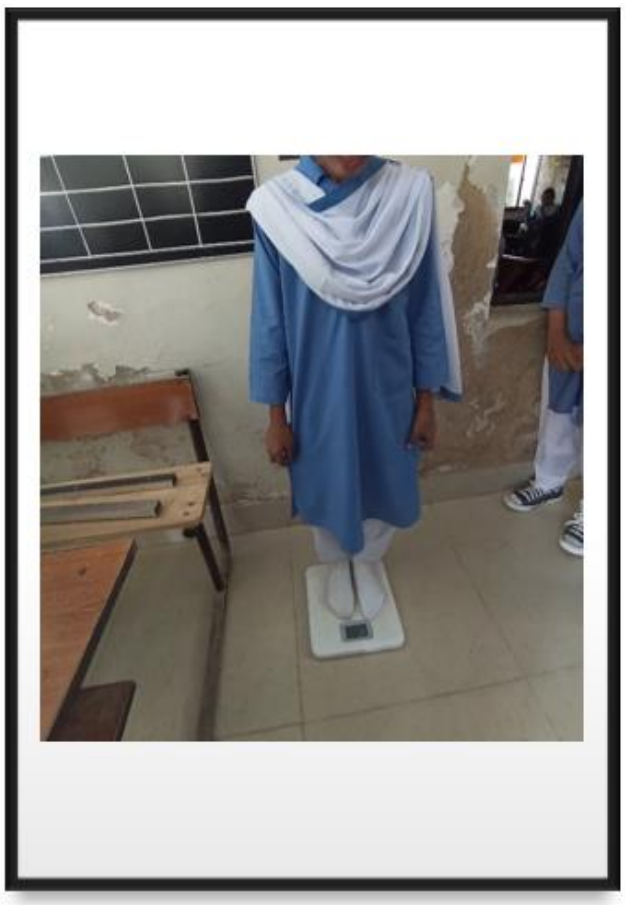
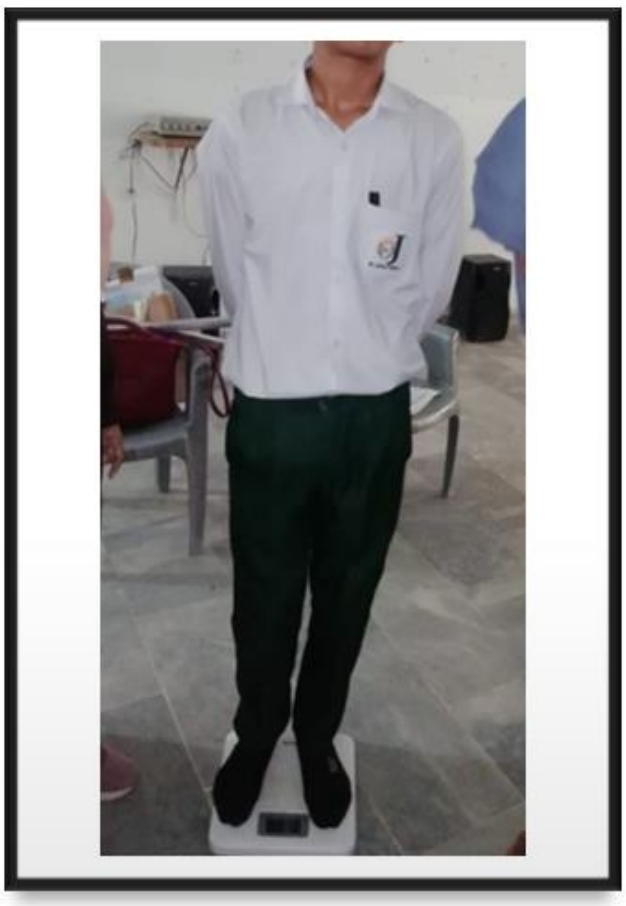

Anthropometry measurements of younger peers

5.2

Annex
Youth FGDs

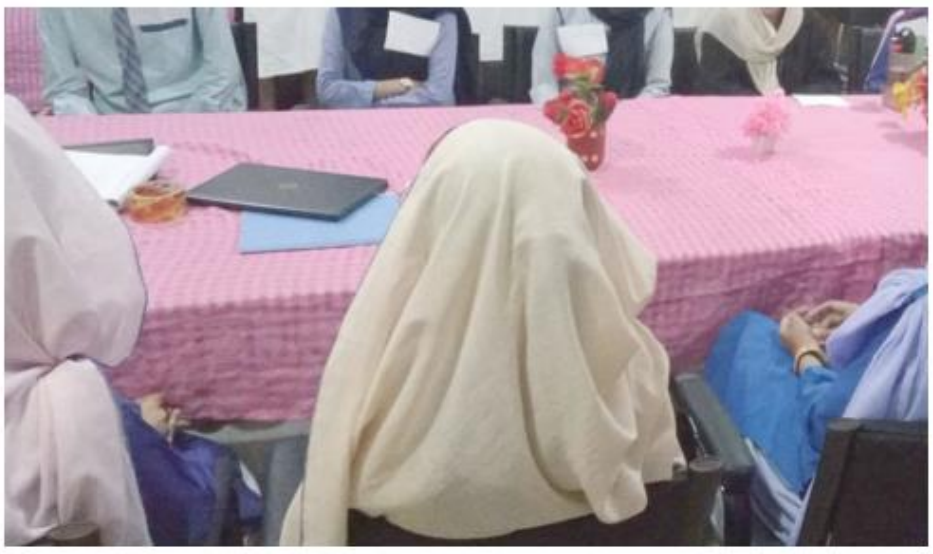

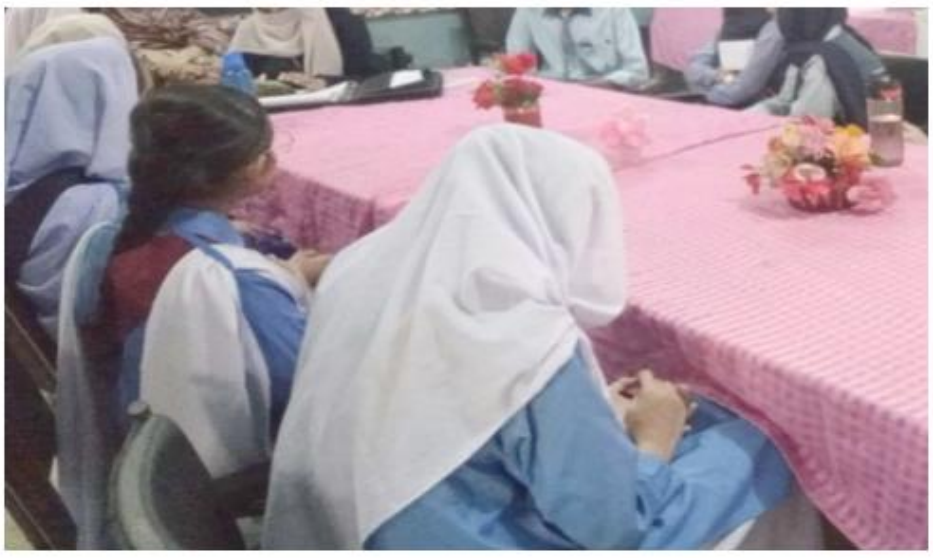

Focus Group Discussions (FGDs) with youth participants

Healthy snacks

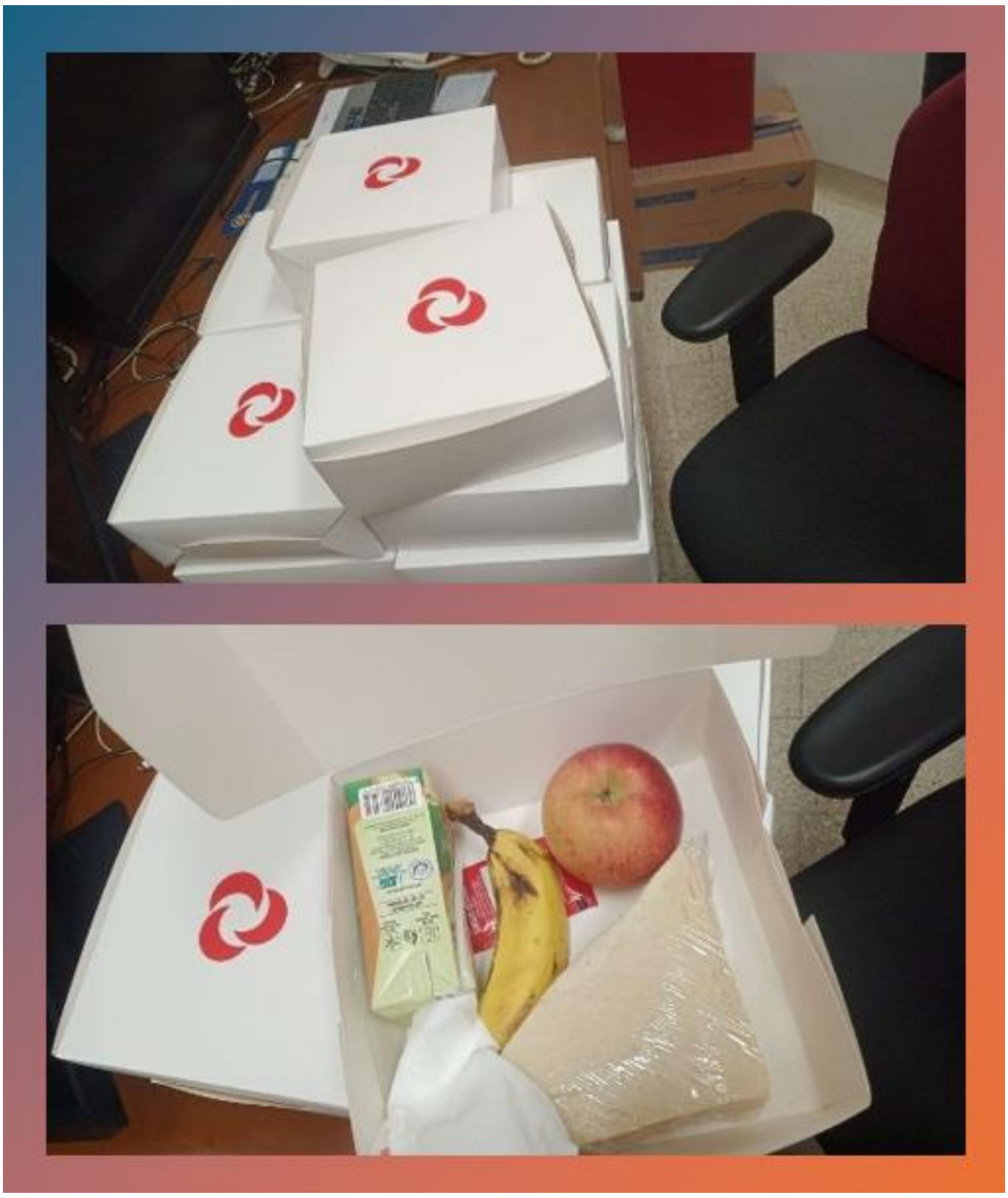

Refreshments distributed during the FGDs and IDIs with the stakeholders

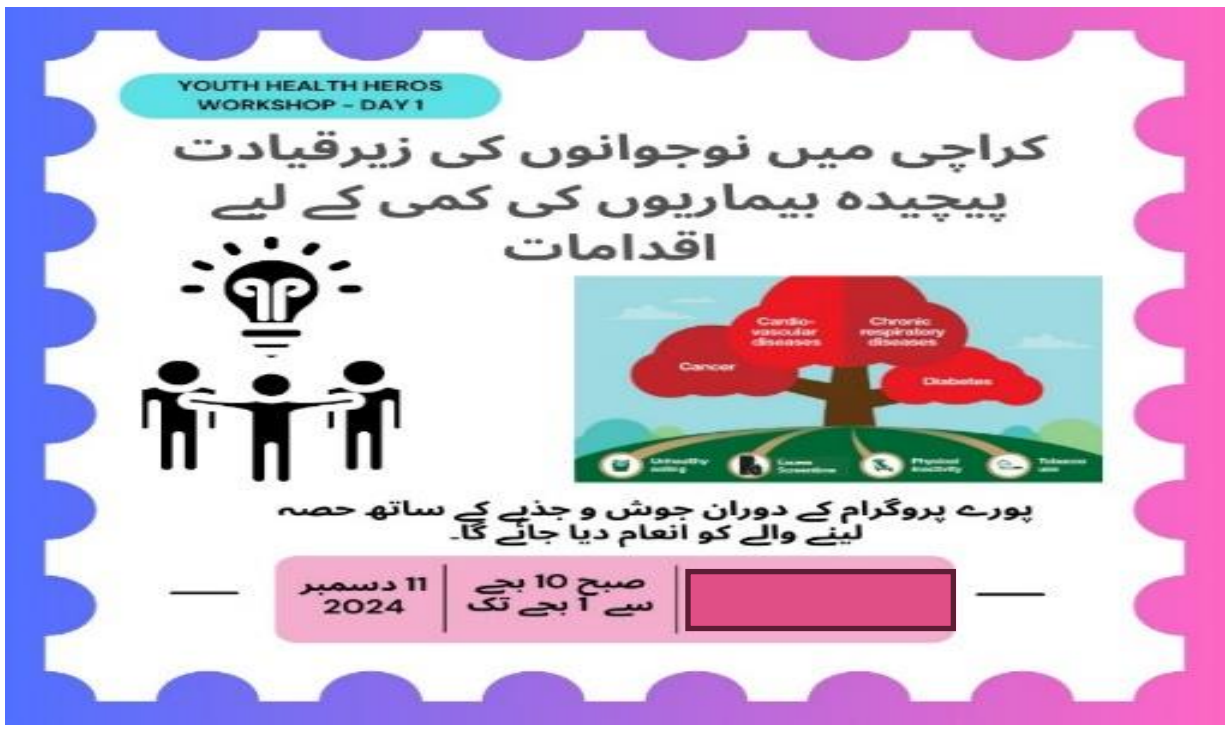

1st Co-creation Workshop information flyer shared with schools, youth, and their families

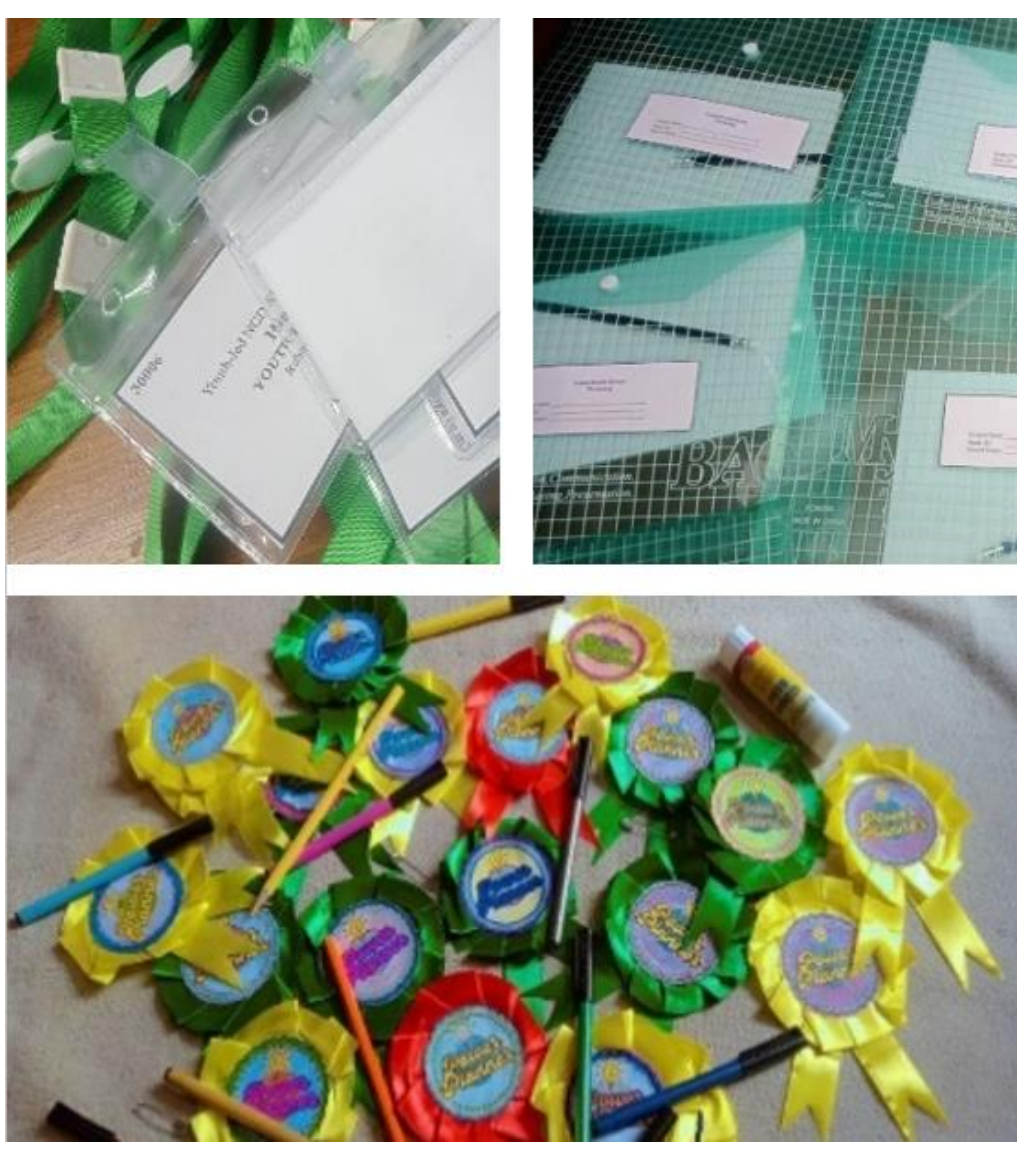

Youth ID cards, files for youth containing workshop agenda, a pencil, a notebook, and voting flags, badges provided to youth for their participation

1st Workshop sessions

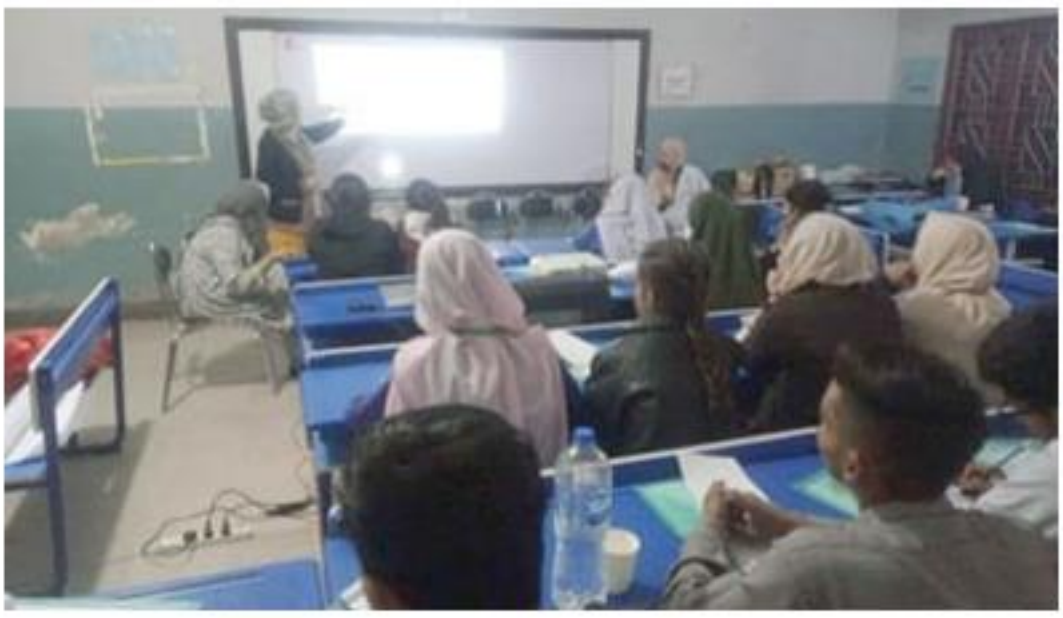

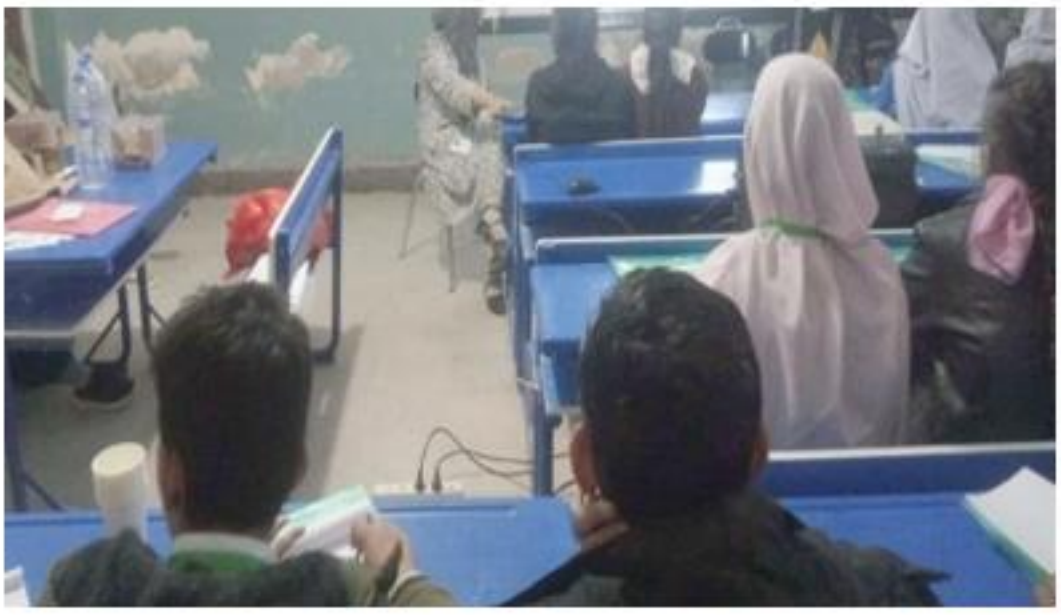

Facilitators conducting sessions during the 1st workshop

1st Workshop group activities

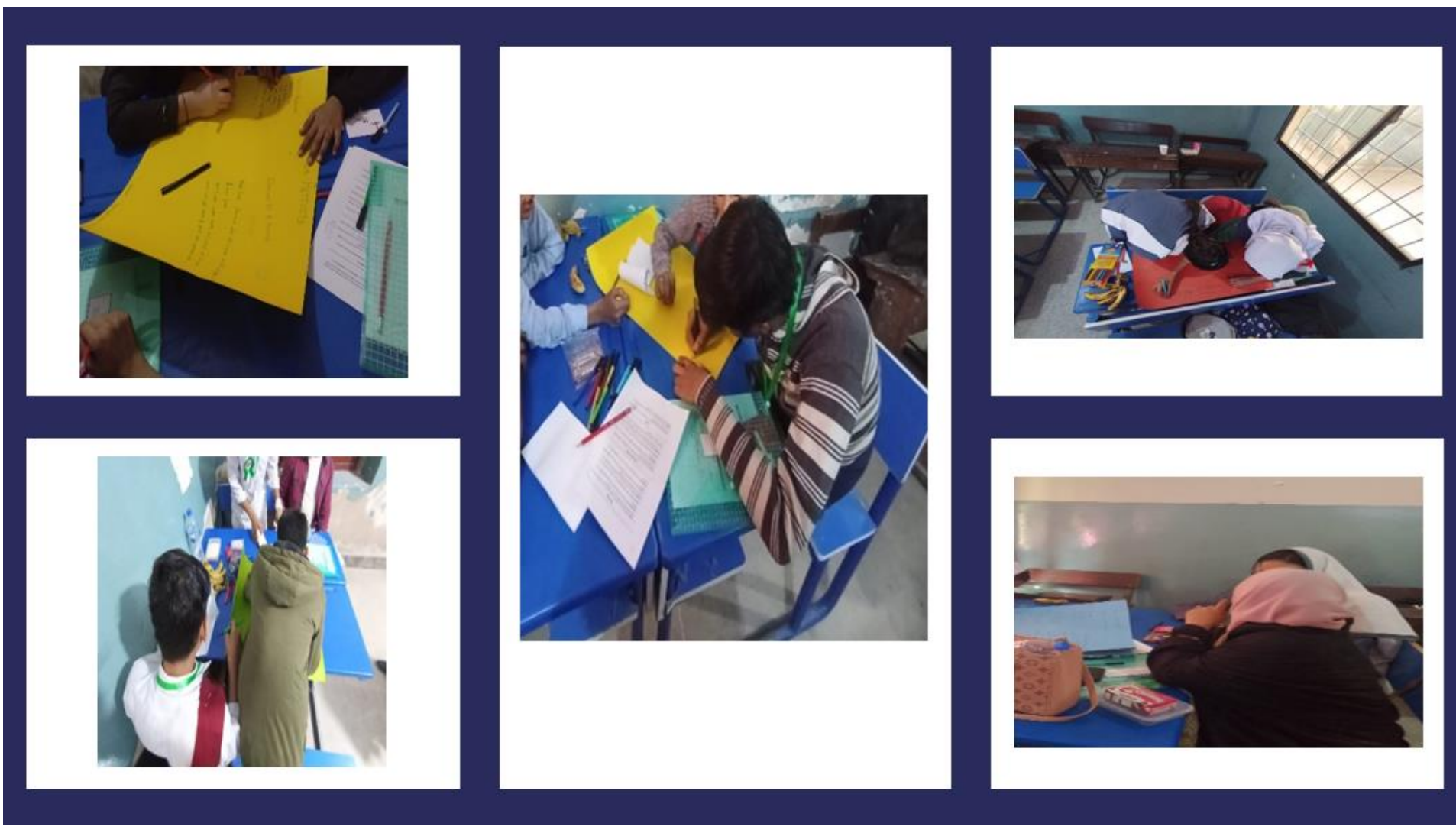

Group activities during the 1st workshop

2nd Workshop

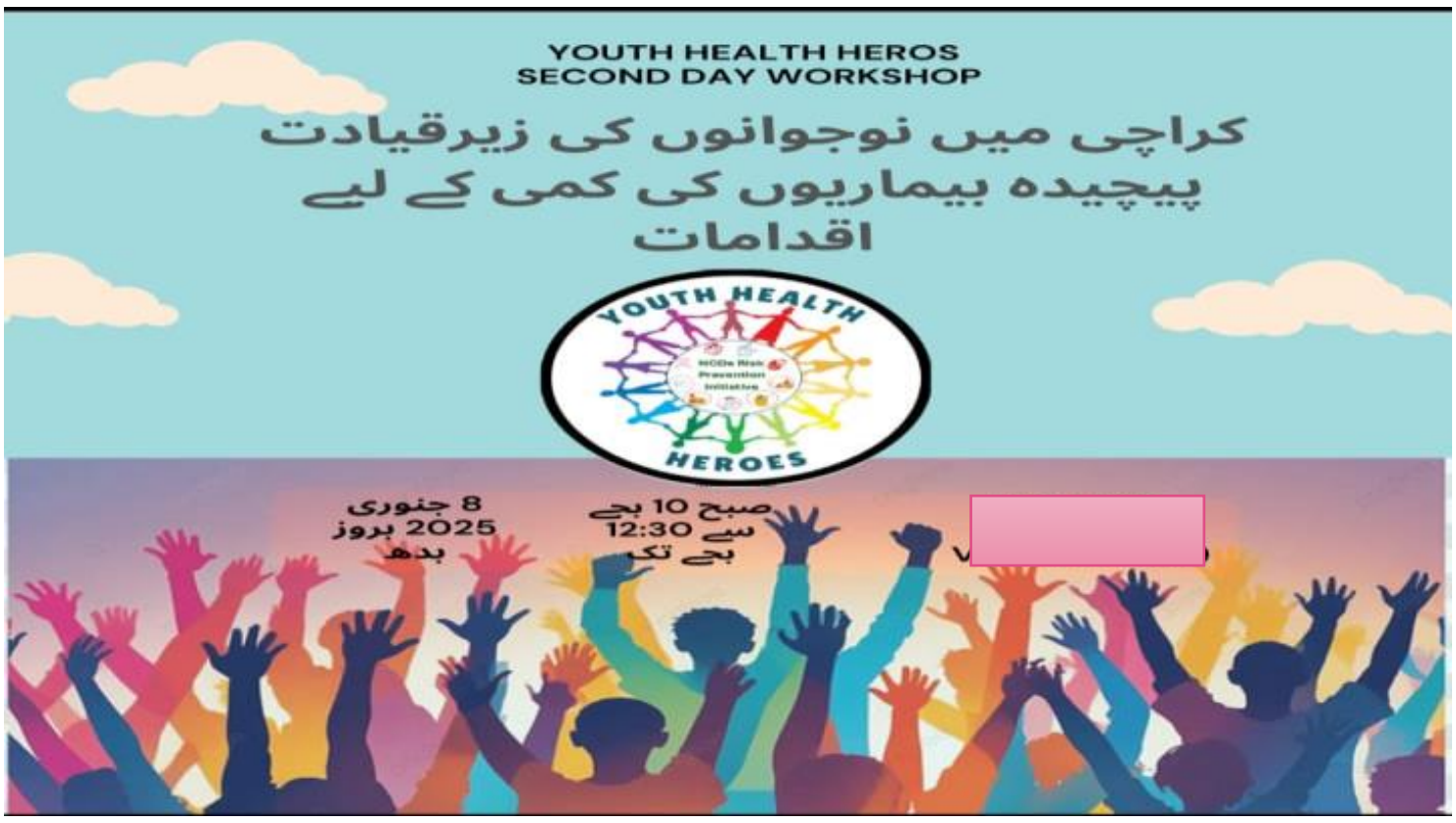

2nd Co-creation Workshop information flyer shared with schools, youth, and their families

2nd Workshop-healthy food & badges

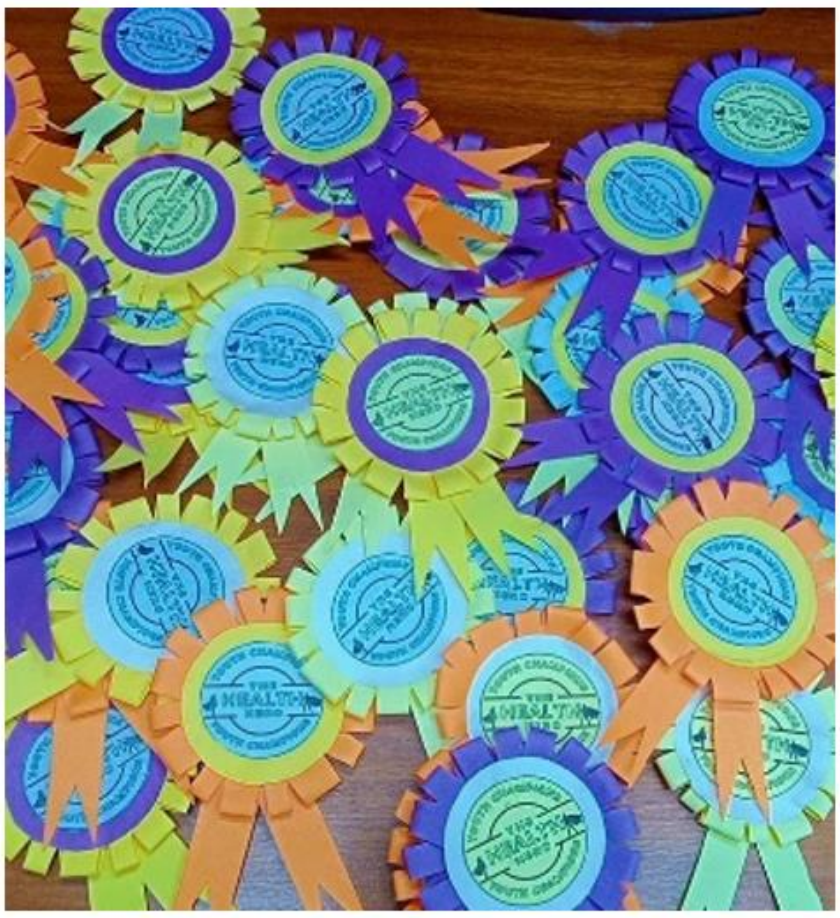

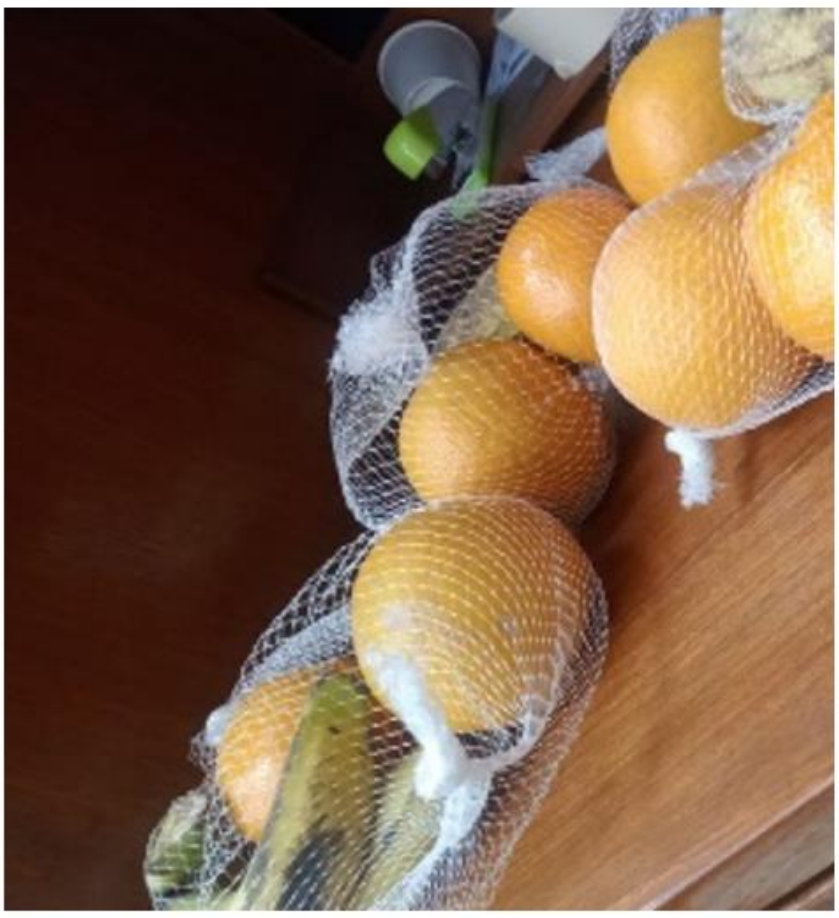

Badges provided to youth for their participation and fruits distribution to encourage healthy eating

2nd workshop activities

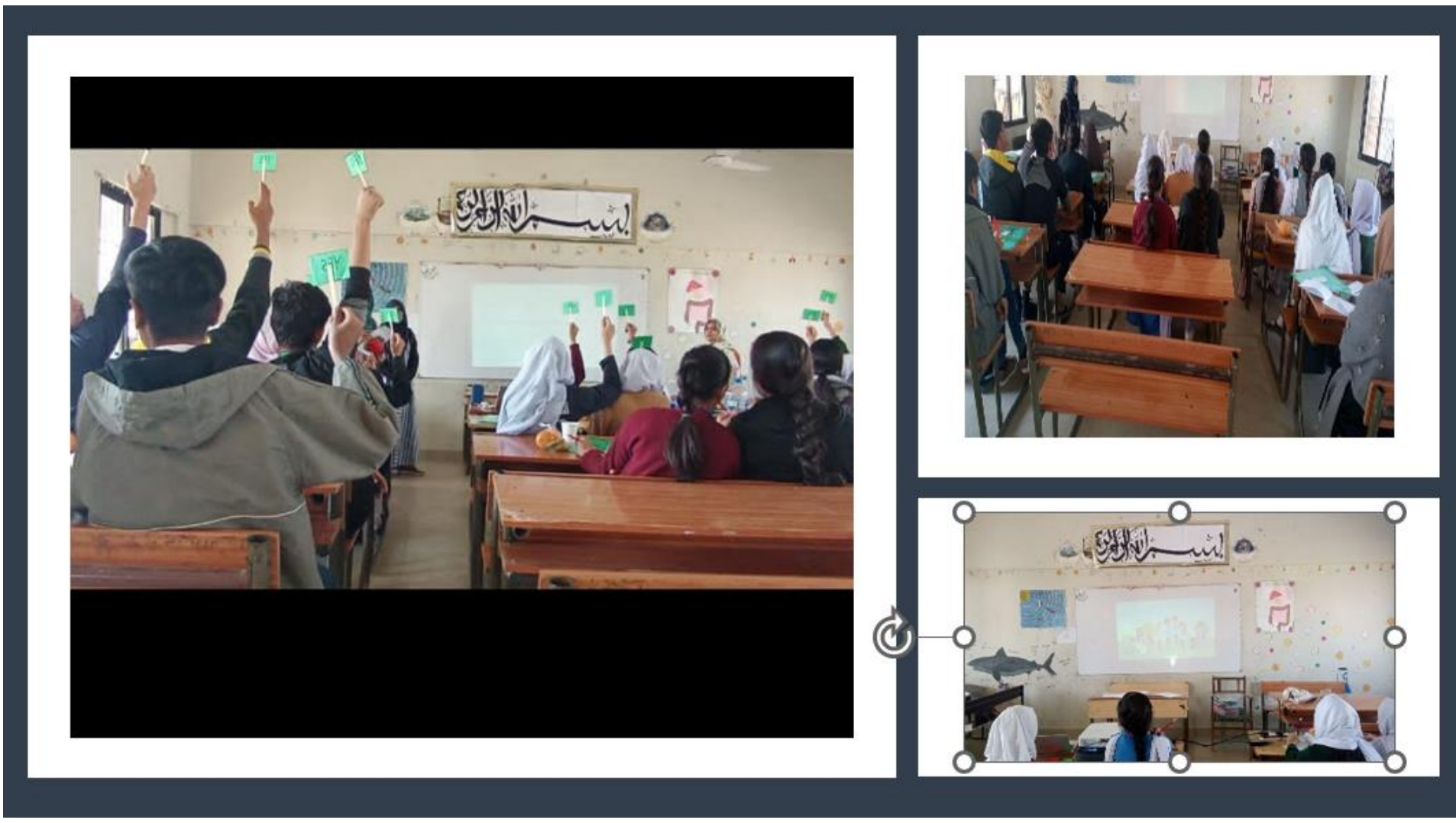

Intervention display and voting during the 2nd workshop

Annex

Training of youth health heroes

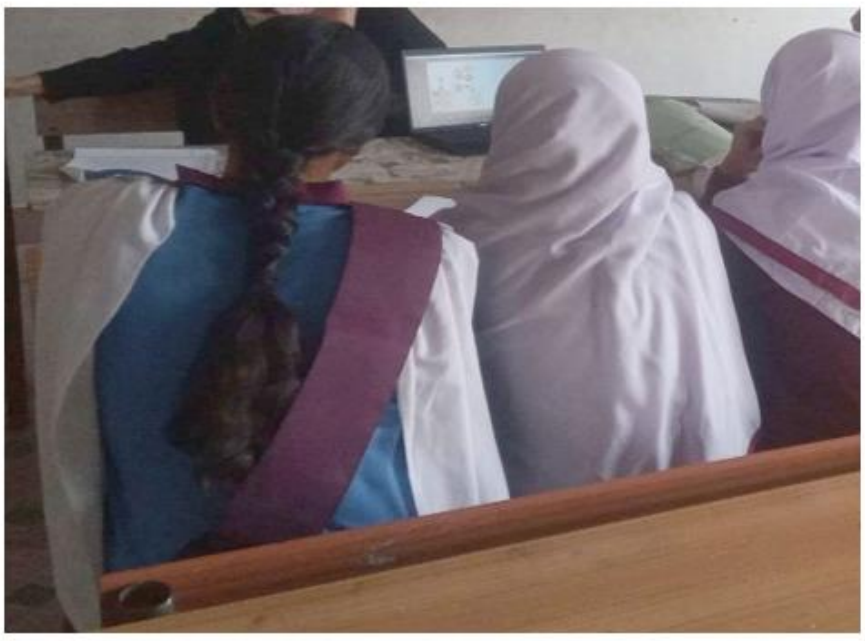

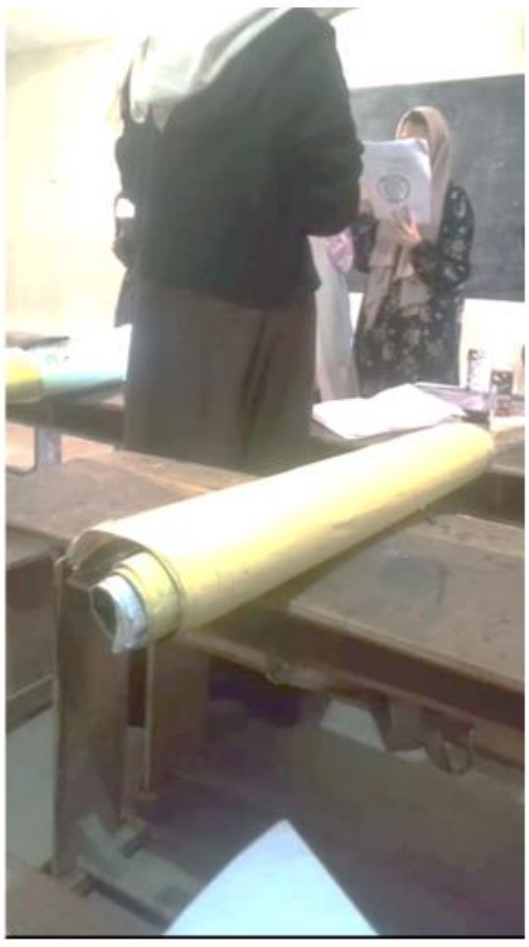

Training of youth health heroes for intervention delivery

Intervention delivery

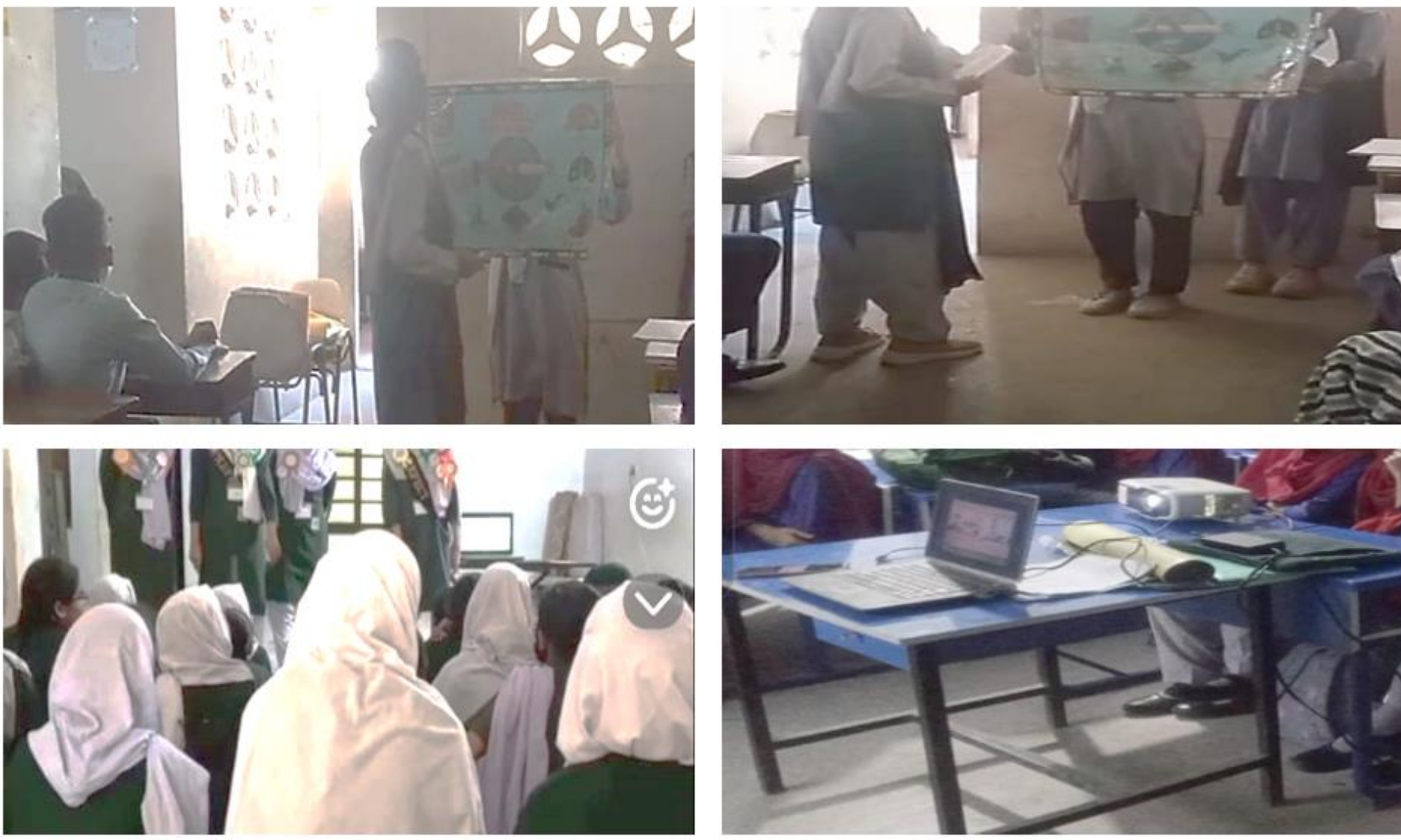

Intervention delivery

5.12

Souvenirs for youth health heroes

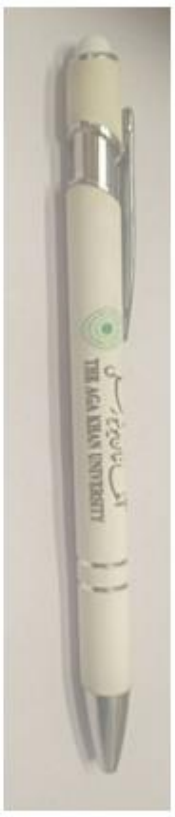

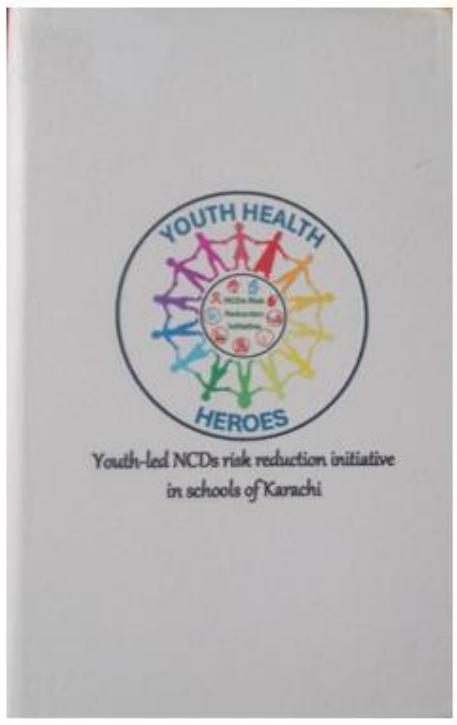

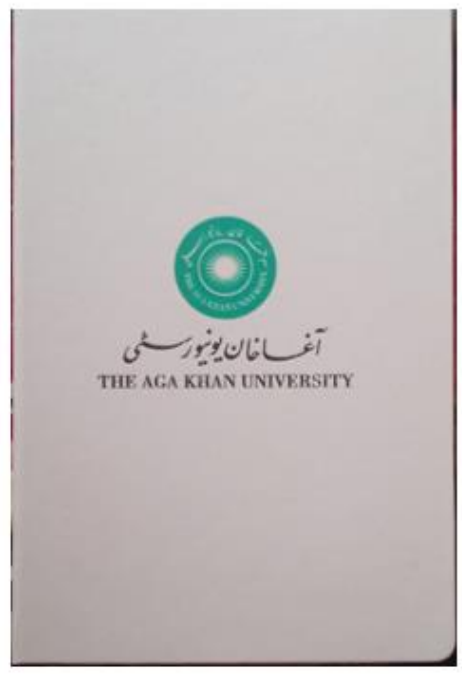

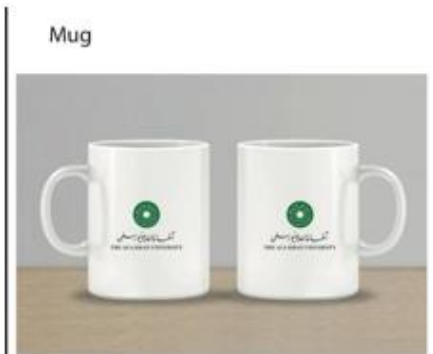

Souvenirs for youth health heroes

5.13

Annex

Certificate for youth health heroes

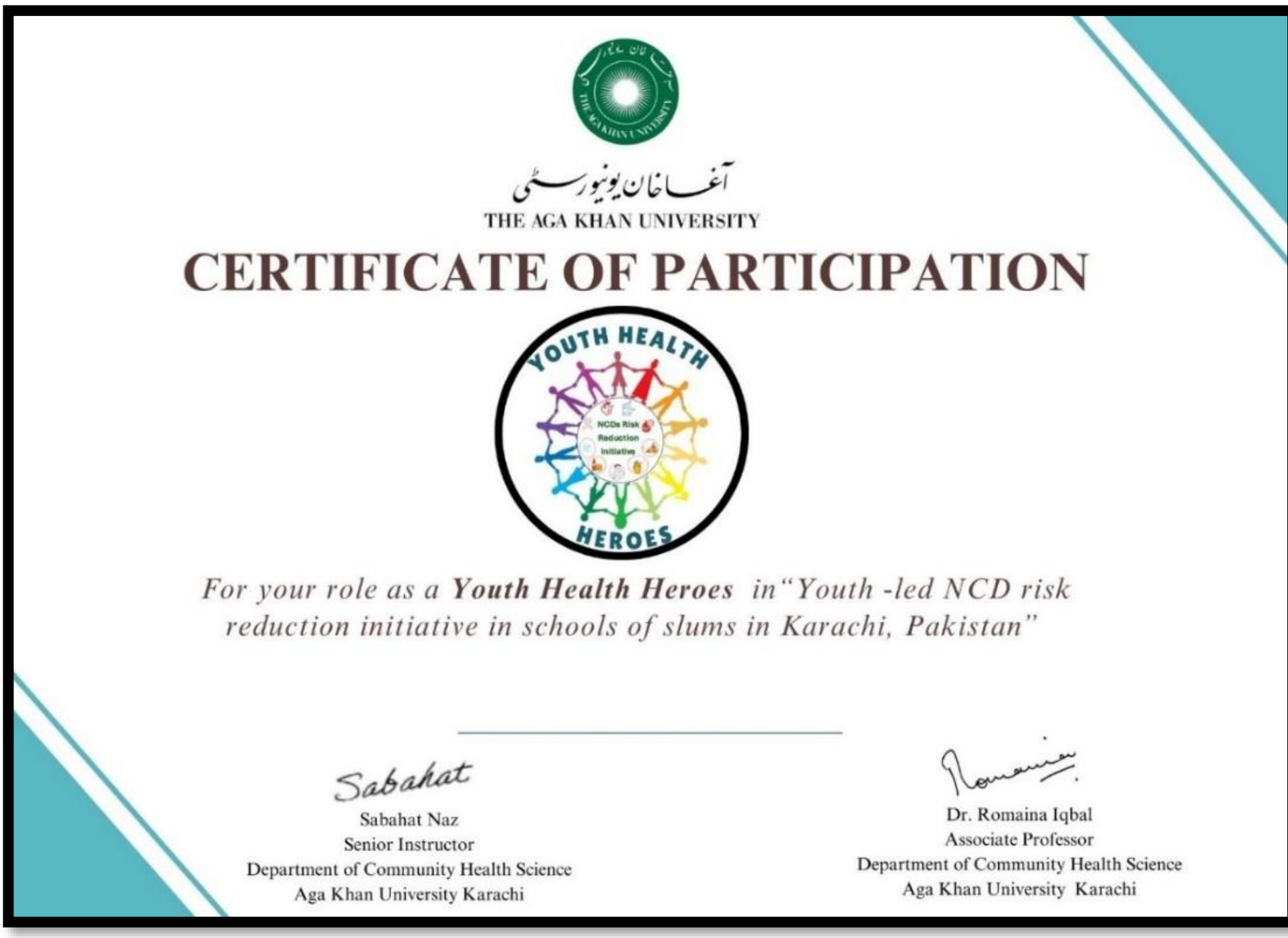

Certificate for Youth Health

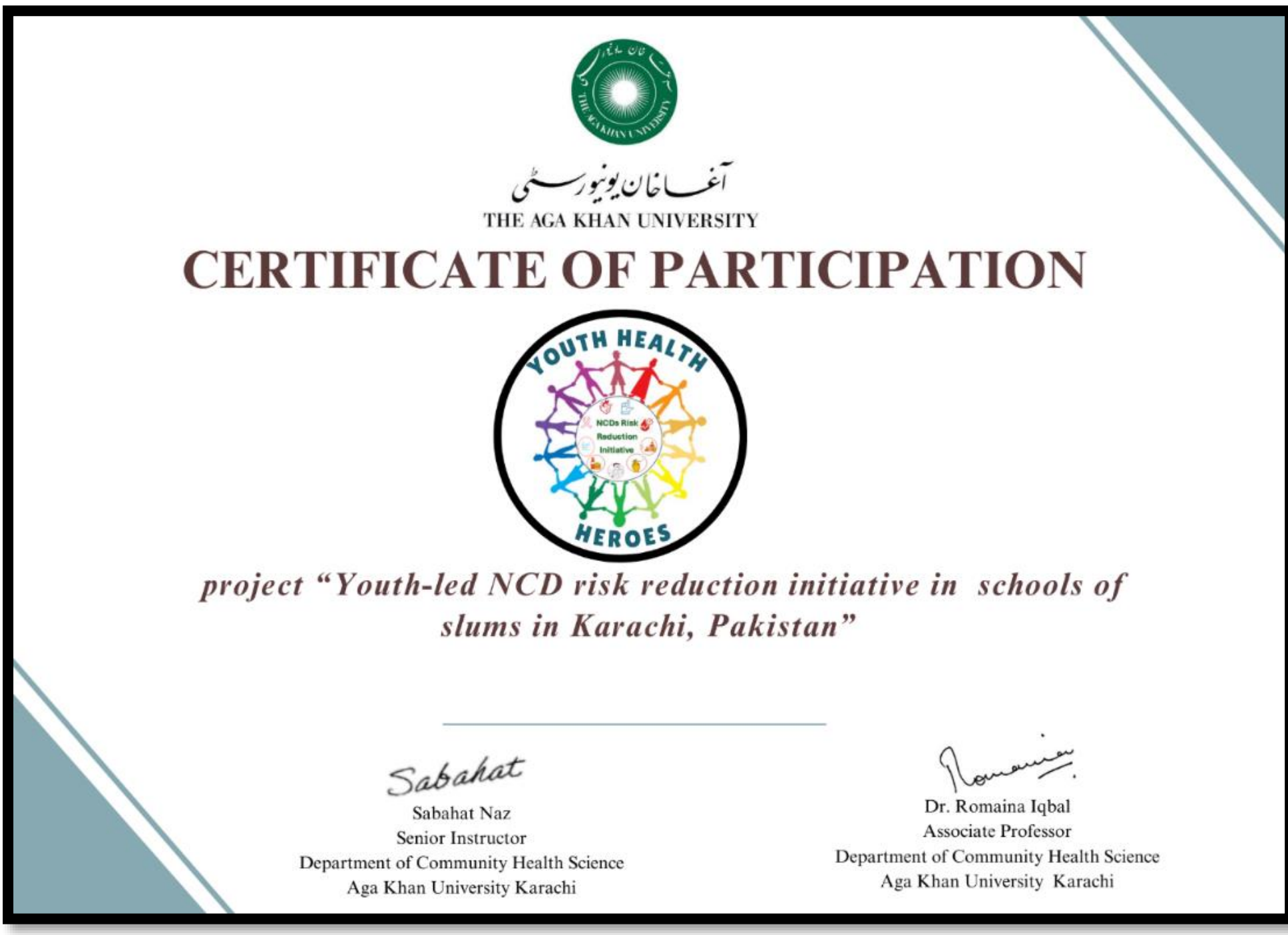
آغا خان یونیورسٹی

THE AGA KHAN UNIVERSITY

CERTIFICATE OF PARTICIPATION

project "Youth-led NCD risk reduction initiative in schools of slums in Karachi, Pakistan"

Sabahat Naz
Senior Instructor
Department of Community Health Science
Aga Khan University Karachi

Dr. Romaina Iqbal
Associate Professor
Department of Community Health Science
Aga Khan University Karachi

Certificate for Schoolteachers

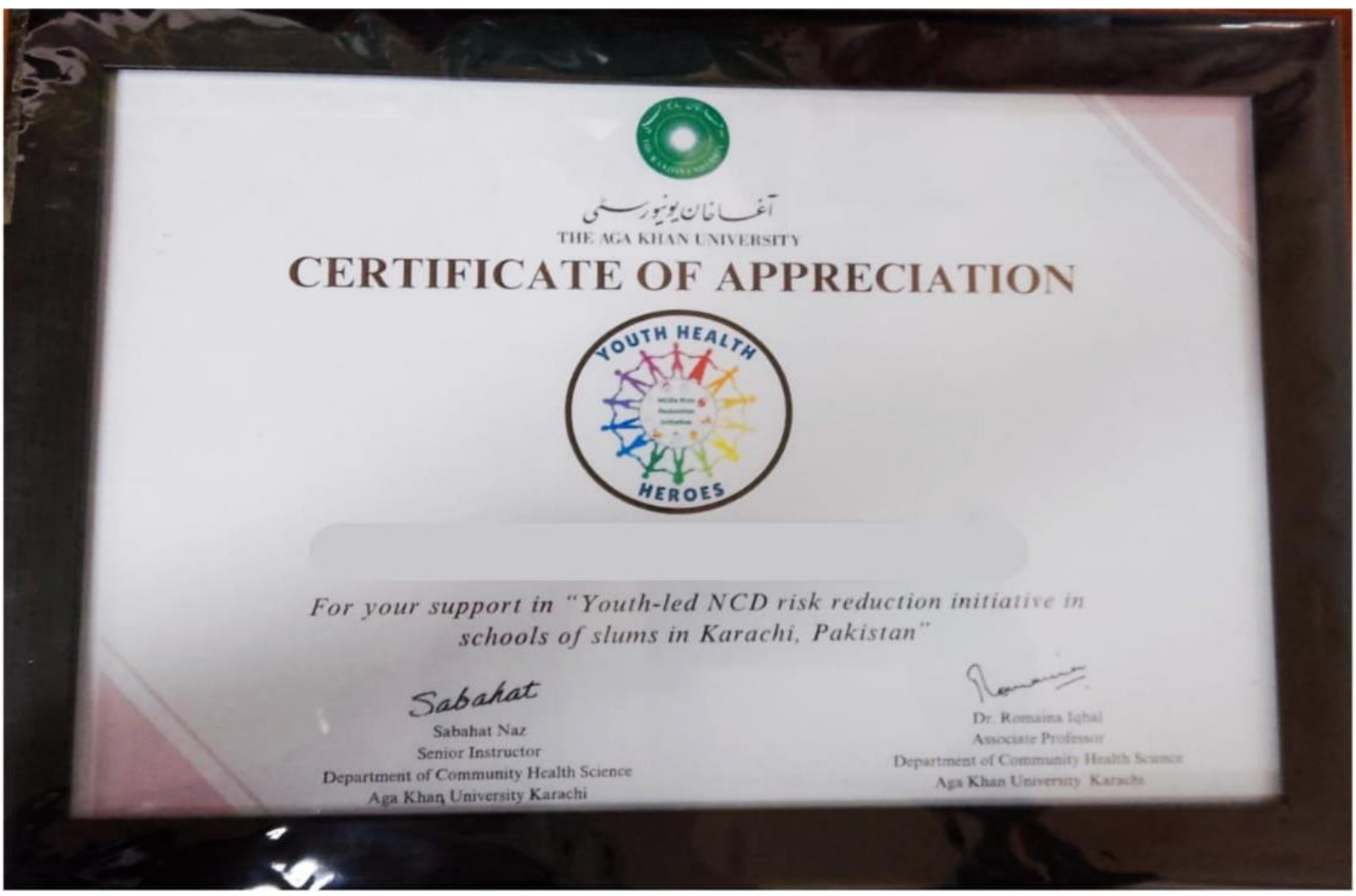

Certificate for Schools
