# Supplementary material for: Youth-led co-creation of a non-communicable disease risk reduction intervention in urban informal settlements of Karachi, Pakistan: a participatory action research
Source: BMJ Public Health. 2026 Nov 5;4(4):e005286. doi: 10.1136/bmjph-2026-005286 (PMC13756321; doi:10.1136/bmjph-2026-005286)
Supplement: Supplementary file 7 [file bmjph-4-4-s007.pdf]

Pilot and Feasibility Studies

STUDY PROTOCOL

Open Access

Feasibility of co-creating and delivering a youth-led noncommunicable diseases risk reduction intervention in schools of slums in Karachi, Pakistan: protocol for a mixed-methods, pre- and post-intervention study

Sabahat Naz[1*], Munazzah Ilyas[1] and Romaina Iqbal[1]

Abstract

Background Adolescents are more susceptible to the shared risk factors of noncommunicable diseases (NCDs), including physical inactivity, tobacco use (smoking and smokeless), and unhealthy diets. However, there is limited literature from Pakistan that involves school-going adolescents in developing and delivering NCD risk-reduction interventions among their younger peers.

Objective This study aims to assess the feasibility of co-creating and delivering a youth-led NCD risk reduction intervention in schools of slums in Karachi, Pakistan.

Methods Using a mixed-methods, pre- and post-intervention design approach, we will conduct the study in 10 randomly selected schools (5 government and 5 private) in Azam Basti and Mehmoodabad, the urban slums in Karachi. The study comprises five sequential phases: Phase I involves identifying shared risk factors of NCDs among younger peers aged 9 to 12 (grades 5 and 6 students) ($n = 100$) using structured diaries, which will also serve as a pre-intervention assessment. Phase II entails conducting qualitative interviews with youth aged 14 to 16 (grades 9 and 10 students) ($n = 40$), their parents, school head teachers, class teachers, and canteen staff to discuss intervention components, materials, and delivery methods. In Phase III, workshops will be held to co-create the intervention with the help of youth. Phase IV will involve 3 days of comprehensive youth training by a qualified nutritionist on delivering the intervention to their younger peers. Finally, in Phase V, youth will deliver the intervention among all grades 5 and 6 students in their schools, followed by a post-intervention assessment, in which the younger peers will complete the structured diaries for the shared risk factors. Two independent data collectors will also perform fidelity checks of the intervention delivery using a checklist. The expected outcomes will include the feasibility of developing and implementing a culturally relevant youth-led NCD risk reduction intervention and youth training manual. The feasibility of the intervention will be assessed using the recruitment and retention rates for schools, youth, and younger peers, youth attendance at workshops and training sessions, structured diaries completion rates

*Correspondence:
Sabahat Naz
sabahat.naz1@aku.edu

© The Author(s) 2025. **Open Access** This article is licensed under a Creative Commons Attribution-NonCommercial-NoDerivatives 4.0 International License, which permits any non-commercial use, sharing, distribution and reproduction in any medium or format, as long as you give appropriate credit to the original author(s) and the source, provide a link to the Creative Commons licence, and indicate if you modified the licensed material. You do not have permission under this licence to share adapted material derived from this article or parts of it. The images or other third party material in this article are included in the article's Creative Commons licence, unless indicated otherwise in a credit line to the material. If material is not included in the article's Creative Commons licence and your intended use is not permitted by statutory regulation or exceeds the permitted use, you will need to obtain permission directly from the copyright holder. To view a copy of this licence, visit http://creativecommons.org/licenses/by-nc-nd/4.0/.

at pre- and post-intervention, the fidelity of delivering the intervention, and the preliminary change in the risk factors at post-intervention compared to the baseline.

Ethics and dissemination The study was approved by the Ethical Review Committee of the Aga Khan University (ref.: 2024–9763-29,256). If the intervention proves feasible, we will disseminate our findings to both academic and nonacademic audiences. Additionally, we will implement a definitive trial across various slum settings in Karachi, Pakistan.

Trial registration ClinicalTrials.gov, NCT06465771. Registered 20 June 2024—prospectively registered, https://clinicaltrials.gov/study/NCT06465771.

Keywords Noncommunicable diseases, Youth, Shared risk factors, Slums, Intervention co-creation, Adolescent health

Background

Noncommunicable diseases (NCDs) account for 41 million deaths each year, which is attributable to 74% of all deaths globally. Unfortunately, 77% of all these deaths occur in low- and middle-income countries (LMICs) [1]. According to findings from the Lancet Commission on Adolescent Health and Well-Being, people aged 10 to 24 years reported a higher burden of NCDs, accounting for 56% of global disability-adjusted life-years (DALYs) and 38.8% of all deaths [2]. Children and adolescents are more susceptible to NCD risk factors, including unhealthy diets, physical inactivity, tobacco use (both smoking and smokeless), and air pollution [1].

Developing countries have faced a rapid transition toward globalization and urbanization over the last few decades [3]. This drastic shift has led to a rapid surge in the prevalence of NCDs, such as diabetes (16%), hypertension (40%), and cardiovascular diseases (17%) in Pakistan [4]. It is estimated that more than 70% of people developing these diseases during adulthood are associated with health-related behaviors that have been initiated during childhood and adolescence [5, 6]. For instance, a recent study from Pakistan found a higher prevalence of metabolic syndrome (17%) among marginalized school-going adolescents, with 8% being physically inactive and 6% having lower fruit intake [7]. In addition, increasing rates of obesity among school-going children due to poor dietary habits and physical inactivity predispose them to many chronic conditions later in life [8]. Estimates from the National Nutrition Survey of Pakistan found that about one in every six boys (18%) and girls (17%) aged 10 to 19 are overweight or obese [9].

The health situation of children in slums is compromising due to the drastic change in the size of slums in Pakistan [10]. For instance, children are more prone to unhealthy lifestyle choices due to the lack of basic facilities, such as improper housing conditions and lack of basic health facilities [10]. Moreover, adopting poor dietary patterns, sedentary behaviors, and increased use of tobacco products among children makes them more susceptible to chronic conditions during early adulthood. These chronic conditions further increase the risk of morbidities and premature deaths [10].

To address the issue of inequalities and healthcare gaps, the Sustainable Development Goals (SDGs) highlight the importance of investing in youth-led initiatives to address NCDs [11]. Empowering youth to prevent and reduce the development of shared risk factors may help mitigate the burden of NCDs. Literature from the West also supports the role of youth in educating their younger peers to adopt healthy behaviors [12, 13]. For example, Tarro et al. found that youth-led lifestyle modification among children effectively increased physical activity and reduced junk food consumption [12]. However, there is limited literature from Pakistan that involves youth in developing and delivering NCDs risk-reduction interventions among school-going children. Therefore, this study aims to assess the feasibility of co-creating and delivering a youth-led NCD risk reduction intervention in schools of slums in Karachi, Pakistan.

Study objectives

- To identify shared risk factors of NCDs among school-going children in selected schools of slums in Karachi, Pakistan
- To co-create a youth-led NCD risk reduction intervention for school-going children with the involvement of key stakeholders
- To assess the feasibility of co-creating and delivering the intervention among school-going children in selected schools of slums in Karachi, Pakistan, through the following:
- The recruitment and retention rates for schools, youth, and younger peers
- The attendance rate of youth at workshops and training sessions
- The rate for completing the structured diaries for diet, physical activity, and tobacco use at pre- and post-intervention assessments

- The fidelity of delivering the intervention in schools
- The preliminary change in the risk factors at post-intervention compared to the baseline

Methods

Study design, setting, and duration

We will conduct a school-based intervention study using a mixed-methods, quasi-experiment (pre- and post-intervention) design. The study will be carried out in five sequential phases (Fig. 1): identifying NCD risk factors through structured diaries (Phase I), followed by qualitative interviews (Phase II), intervention co-creation (Phase III), training of youth (Phase IV), and intervention delivery (Phase V). The study setting will be two urban slums in Karachi, namely Azam Basti and Mehmoodabad. The slums are situated in the District East. People living in these slums belong to low- and middle-income families with poor access to healthcare facilities. The study commenced in September 2024 and will last for 1 year.

Identifying and recruiting schools

We have already sought permissions from the district education officer of elementary, secondary, and higher secondary schools and the Directorate of Government and Private Schools, District East, Karachi. We have also received a list of government and private schools situated in Azam Basti and Mehmoodabad. Using the list, we will contact the school administrators to identify eligibility and send the school head teacher a letter containing brief study information. During the recruitment process, both government and private schools will be ensured to participate. Using the eligibility criteria, we will randomly recruit 10 schools, 5 from each public and private sector.

Recruiting youth and younger peers

We will recruit 40 youth from grades 9 and 10 (aged 14 to 16), i.e., 4 from each school. Students in grades 9 and 10 are senior members of their schools and are in the middle of adolescence. At this stage, they serve as role models for younger peers and take on leadership roles, making them well-suited to influence and promote positive behaviors among their younger peers [12, 13]. We request that schools provide a list of all eligible students in grades 9 and 10 based on the study eligibility criteria. Using the list, we will send a study information sheet along with parental consent to the parents of randomly selected youth through schools. Those who provide parental consent will be invited to participate in the study, and written assent will be obtained before enrollment.

Similarly, we will recruit 100 younger peers (aged 9 to 12) from grades 5 and 6, i.e., 10 from each school. In grades 5 and 6, students are in early adolescence, a critical period for establishing lifelong health-related behaviors [14]. These behaviors can significantly impact their overall health and well-being both in the short and long term [15]. This stage presents a valuable opportunity for early intervention to promote healthy habits and reduce the risk of developing NCDs later in life. We will request schools to provide a list of all eligible students from grades 5 and 6 based on the study eligibility criteria. Using the list, we will send a study information sheet along with parental consent to the parents of randomly selected younger peers through schools. Those who provide parental consent will be invited to participate in the study, and written assent will be obtained before enrollment.

During the recruitment, we will give the boys and girls an equal opportunity to participate as youth and younger peers. In addition, students will have the right to withdraw from the study at any point without facing any consequences. This ensures that their participation is entirely voluntary, and that they feel comfortable making decisions that are best for their well-being.

Eligibility criteria

Inclusion for schools and their staff

- Both government and private schools

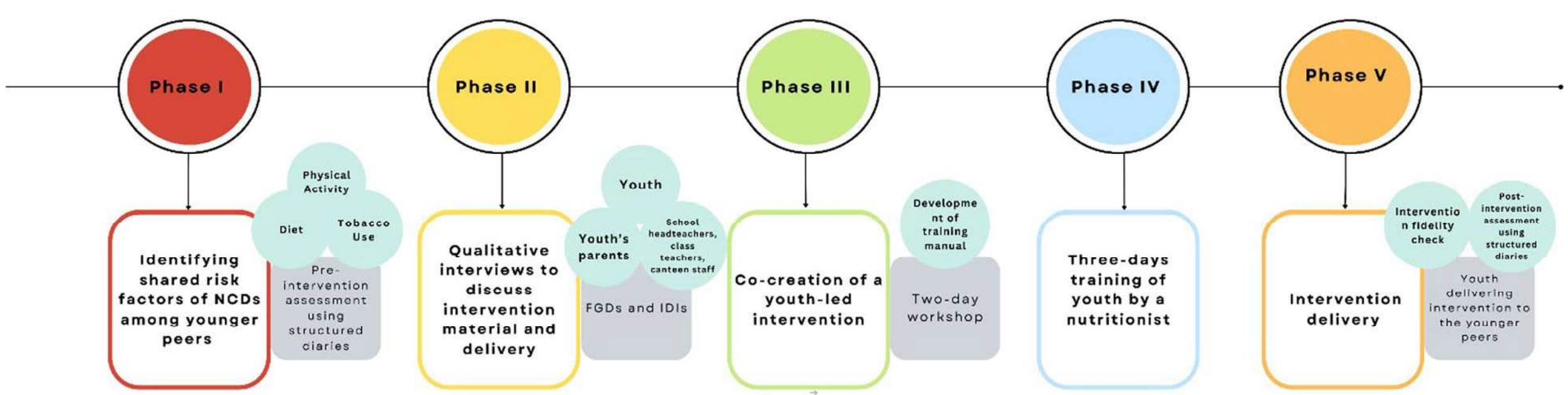

Fig. 1 The study workflow

- Schools that have grades 1 to 10 in one boundary
- Schools who agree to take part in the qualitative interviews
- Schools that permit to engage youth, their parents, and younger peers in the study for 10 months
- School head teachers, class teachers (grades 5, 6, 9, and 10), and canteen staff who provide written informed consent

Inclusion for youth and younger peers

- For youth: Students of grades 9 and 10 (aged 14 to 16 years)
- For younger peers: Students of grades 5 and 6 (9 to 12 years)
- Students who provide written informed assent along with parental consent

Inclusion for parents

Parents of youth who agree to participate in focus-group discussions (FGDs) and provide written informed consent

Exclusion for schools

- Schools currently enrolled in any NCD-related intervention program or any similar activity during the last 6 months
- Schools who do not use Urdu/English as their education medium

Exclusion for youth and younger peers

- For youth: Students with severe or chronic medical conditions or disabilities that prevent active participation in the intervention co-creation and delivery
- For younger peers: Students with severe or chronic medical conditions or disabilities that prevent them from completing structured diaries

Intervention co-creation framework

We will adopt an integrated co-creation framework by combining Youth Participatory Action Research (YPAR) [16] and intervention mapping (IM) [17] (Fig. 2). The working methodology of YPAR is rooted in the principles of participatory action research (PAR) [16], which advocates involving the target population (youth) in every step of the process, as discussed in Table 1. YPAR ensures that youth actively contribute to decision-making, and

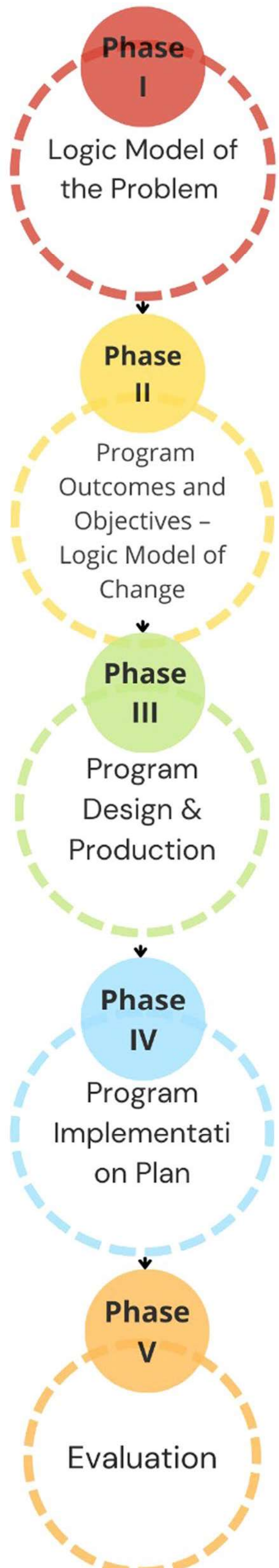

Fig. 2 Intervention mapping framework

Table 1 Stakeholder mapping

Name<br>Identify stakeholder and groups	Involvement<br>What level of involvement is expected	Anticipated issues<br>Known or predicted issues	Issues mitigation<br>What strategies will be used to mitigate these issues	Milestones<br>At what point is the stakeholder's involvement required	Priority
Youth (students of grades 9 & 10)	Complete involvement	Lost to follow-up, lack of cooperation	Build trust, clear and friendly communication, regular updates, incentives, parents and teachers involvement	Throughout the study	High
Parents of youth	Partial involvement	Low-response rate, especially from fathers	Flexible schedules, clear communication of the importance of the issue, reimbursement of transportation cost	Phase II (FGDs)	High
Head teachers	Partial involvement	Lack of cooperation due to busy schedule	Flexible schedules, clear communication of the importance of the issue, incentive and recognition	Phase II (IDIs)	Moderate
School teachers (grades 5, 6, 9, & 10)	Complete involvement	Lack of cooperation due to busy schedule	Flexible schedules, clear communication of the importance of the issue, incentive and recognition	Throughout the study	High
Canteen staff	Partial involvement	Lack of willingness and ability	Flexible schedules, clear communication on the importance of their role, incentive and recognition	Phase II (IDIs)	Low

IDIs In-depth interviews, *FGDs* Focus-group discussions

that outcomes align with their preferences [18]. IM provides a structured framework to help plan, implement, and evaluate the intervention, ensuring the stakeholders' opinions are incorporated into the intervention [19]. This approach has shown successful results in co-creating interventions targeted at adolescents [13].

Phase I: Identifying shared risk factors of NCDs among younger peers (grades 5 and 6)

Our study focuses on the shared risk factors, such as unhealthy diet, physical inactivity, and tobacco use (smoking and smokeless) of NCDs, including diabetes, heart disease, cancer, and lung disease. To identify the risk factors among younger peers, we will provide a structured diary (Fig. 3) in which they will record their usual diet, physical activity routine, and tobacco use for 4 days, for example, from Thursday to Sunday (2 weekdays and 2 days over the weekend). The structured diaries will be provided in the local language (Urdu), with detailed written instructions on how to fill out the diaries. Through the structured diaries, we will identify the number of days students consume healthy food (fruits, vegetables, milk and dairy products, and meat and its products) as well as unhealthy food (chips, biscuits, french fries, cold drinks, toffee, chocolates, etc.). We will also quantify the daily intake of fruits and vegetables. In addition, we will measure meal-skipping patterns by identifying how often students skip breakfast, lunch, or dinner. We will assess the frequency and amount of daily physical activity and screen time. We will also identify the prevalence and types of tobacco product use (smoking and/or smokeless tobacco) and betel nut consumption among students. The information collected through the structured diaries will serve as a pre-intervention assessment. We will also measure the height and weight of all randomly selected students.

Phase II: Qualitative interviews with youth and key stakeholders

To co-create a youth-led NCD risk reduction intervention, focus-group discussions (FGDs) and in-depth interviews (IDIs) will be conducted with youths, their parents, and other stakeholders (head teachers, school teachers, and canteen staff) (Table 1). We will perform all interviews on school premises after getting permission from schools. During the FDGs and IDIs, we will discuss the findings of Phase I, i.e., the shared risk factors among grades 5 and 6 students (younger peers). Using the

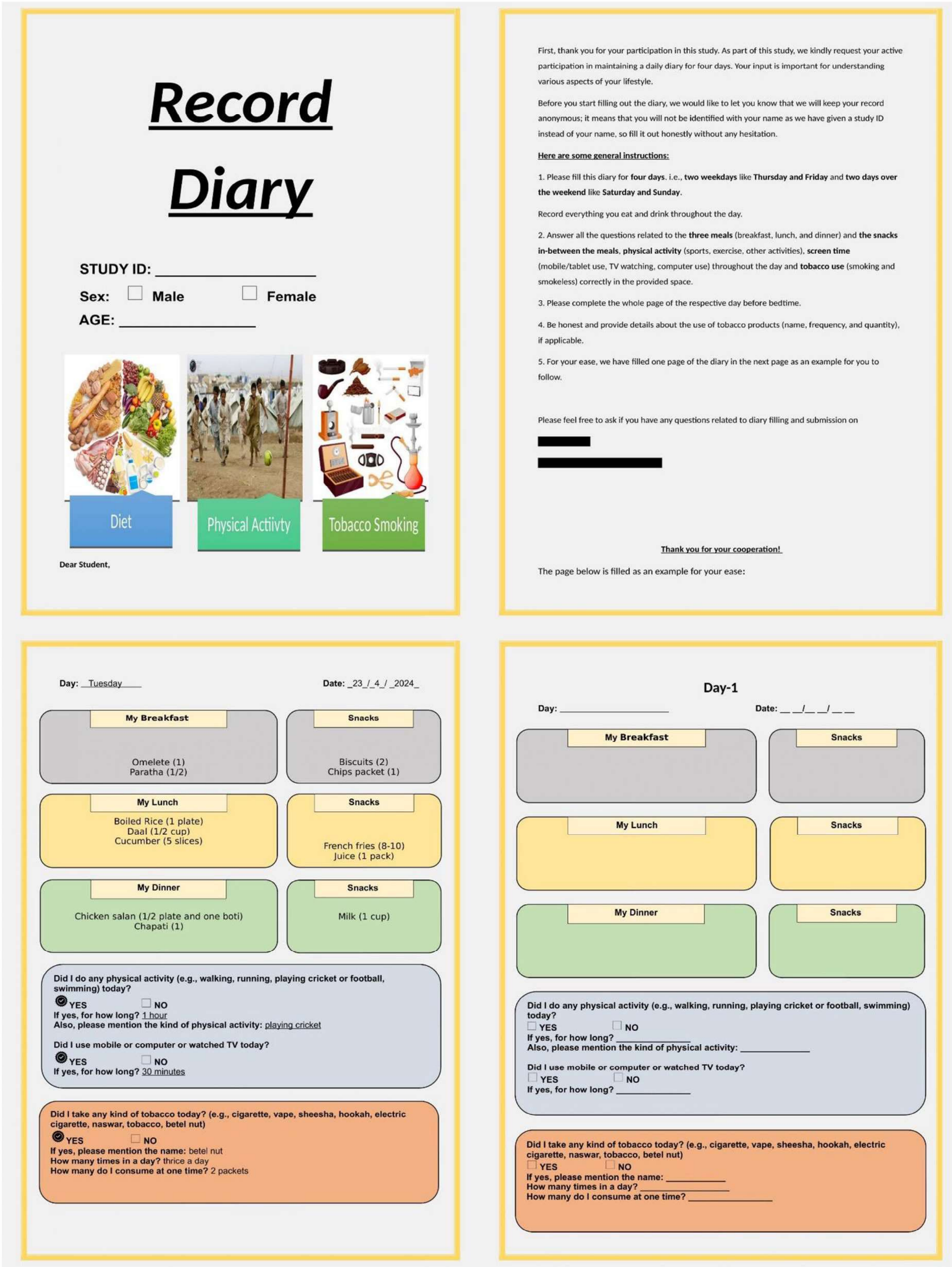

Fig. 3 Structured diaries for pre- and post-intervention assessments

semi-structured interview guides, a trained researcher will conduct FDGs and IDIs in the local language (Urdu). During the interviews, we will explore ideas about the intervention components, content, format, and delivery. In addition, we will specifically understand stakeholders' perspectives on the feasibility of implementing the intervention within schools in terms of the number of sessions, duration of each session, timing of intervention delivery, technology integration, language, etc., to identify potential challenges, required resources, and acceptability within the school setting.

Focus-group discussions (FGDs) We will conduct FGDs with youth. We will invite all youth ($n = 40$) to participate in FGDs. We plan to conduct four to five FGDs with youth (8–10 youth per FGD) or until we reach data saturation. We will discuss the format of the intervention with the youth by giving some examples like storytelling, videos, infographics, pamphlets, interactive sessions, and games.

Similarly, we will conduct FGDs separately with their parents. Consent will be sent to youth parents through schools, and those who provide written informed consent will be invited to participate in FGDs. We aim to conduct at least 5–6 FGDs with parents (8–10 parents per FGD) or until we reach data saturation. In addition to the questions related to intervention material and its content, we will discuss strategies to involve youth while developing and delivering the intervention actively, taking ownership, and becoming advocates for reducing NCD's shared risk factors among their younger peers.

In-depth interviews (IDIs) We will conduct IDIs with key stakeholders, including head teachers, school teachers, and canteen staff. We will provide complete study information to stakeholders and obtain written informed consent before the interview. We will approach stakeholders from all government and private schools. We aim to conduct 5–6 IDIs with each stakeholder group, such as 5–6 IDIs with different head teachers, different class teachers, and different canteen staff. We will also adjust these numbers (increased or decreased) based on achieving data saturation. We will conduct the IDIs using a semi-structured interview guide primarily focusing on questions about the development of an intervention considering the active participation of youth, schools' feasibility, and younger peer involvement.

Qualitative analysis plan We will audio-record all interviews and remove all identifiable information during the transcription. We will transcribe the interviews verbatim into Roman Urdu. The data will then be analyzed using the manual thematic analysis framework approach [20]to identify the key points for intervention co-creation. This approach will involve following the six steps: familiarization, coding, generating themes, reviewing themes, defining and naming themes, and writing up. Following these steps, we will read the transcriptions to familiarize ourselves with the data. The topic guide will guide the development of the thematic analysis framework. Using the deductive thematic analysis approach, we then systematically code the interview data and organize them into macro and micro themes in Excel. The themes will be discussed with the study team, and all macro themes will be finalized and presented with corresponding micro themes. Only the quotes used in the paper will be translated into English.

Phase III: Co-create a youth-led NCD risk reduction intervention

Once the qualitative analyses are finalized, we will conduct 2 days of workshops (approximately 3 h) with the youth to co-create the intervention material and finalize it. With the support of teachers, the workshop will be conducted during school hours on school premises. A trained nutritionist with experience working with school-going children and other study team members will facilitate the workshops. During the workshops, the nutritionist will discuss the findings of structured diaries from Phase I and the qualitative findings from Phase II with the youth. The youth will be actively involved in finalizing the intervention material so that it will be designed in a way that helps them absorb the information as much as possible and deliver it appropriately among their younger peers. In addition, the intervention material will also be mainly based on activities that the younger peers will take an interest in, such as storytelling, video demonstration, and word search activities. During these workshops, we will also develop an intervention training manual to standardize the intervention delivery method and help youth follow it while delivering it to their younger peers. The workshop's detailed plan is presented in Table 2.

Phase IV: Training of youth

Once the intervention is finalized, we will conduct a 3-day training session (each day comprising 3 h) with the youth. This training will cover all four major NCDs: diabetes, heart disease, cancer, and lung disease, focusing on their shared risk factors, which include unhealthy diet, physical inactivity, and tobacco use. The nutritionist will discuss the intervention material that we developed in Phase III with the help of youth. The nutritionist will also train the youth in delivering the intervention among their

Table 2 Workshop detailed plan (Phase III)

Day 1 workshop (10 am to 1 pm)		
Workshop opening	10:00 am–10:30 am	Introduction of the research team, teachers, and youth
Activity 1	10:30 am–10:45 am	A brief introduction to the purpose and goals of the study and co-creation process
Activity 2	10:45 am–11:00 am	Present infographics on the findings of diet, physical activity, and tobacco use obtained through structured diaries in Phase I
Activity 3	11:00 am–11:15 am	Flashcards on the key findings obtained from the qualitative interviews regarding the intervention material
Activity 4	11:15 am–12:15 pm	Brainstorming with youth to generate ideas about the intervention, including components, format, content, timing, number of sessions, and delivery through group activities
Activity 5	12:15 pm–1:00 pm	Presentation of the ideas generated through group activities and open discussion and feedback to refine ideas for intervention co-creation
Workshop concluded with healthy snacks		
Day 2 workshop (10:00 am to 12:30 pm)		
Workshop opening	10:00 am–10:45 am	Recap of the activities from the previous workshop
Activity 1	10:45 am–12:15 pm	Presentation of the developed intervention components, getting feedback and confirmation from youth and teachers
Activity 2	12:15 pm–12:30 pm	Discussing and scheduling dates for the intervention training
Workshop concluded with healthy snacks		
Workshop outcomes<br>• Development of a youth-led intervention<br>• Development of a training manual by the nutritionist		

younger peers. The activities designed for the intervention will also be conducted among youth so that they will understand it and deliver it to their younger peers in the same way. We will organize the training on school premises during school hours. We will train 4 selected youths from each school (a total of 40) who will deliver the intervention among their younger peers.

Phase V: Intervention delivery

Baseline assessment We will consider the findings of Phase I as a baseline assessment for this study, in which 100 younger peers from grades 5 and 6 will complete the structured diaries for their daily diet and physical activity routine and use of tobacco products for 4 days.

Delivering intervention to the younger peers As finalized in Phase III, the four youth from each school will deliver the intervention among their younger peers (all grades 5 and 6 students).

Fidelity index Two independent data collectors will perform the fidelity checks by observing the intervention delivery to assess whether the intervention is delivered as initially designed and intended. We will prepare a fidelity checklist once the intervention is finalized (Phase III). We will randomly select 6 schools (3 government and 3 private) out of 10. The data collectors will attend all intervention sessions that the youth will deliver to their younger peers and score them to assess the fidelity of the intervention using a fidelity index.

Post-intervention assessment After the intervention is delivered, we will conduct the post-intervention assessment. We will provide structured diaries to the randomly selected grades 5 and 6 students (n = 100) who completed the baseline assessment in study Phase I. The students will be requested to record their daily diet, physical activity routine, and tobacco use for 4 days, from Thursday to Sunday (2 weekdays and 2 days over the weekend).

Sample size calculation and study progression criteria for the full trial

This study aims to evaluate the feasibility of co-creating and delivering a youth-led NCD risk reduction intervention in the randomly selected 10 schools of slums (5 from each public and private). We did not conduct a formal size calculation; instead, based on Sim and Lewis, a sample size of at least 50 is required to determine the variability of the feasibility outcomes, such as recruitment and retention rates of students and the rate for completing the structured diaries, and changes in the shared risk factors of NCD at post-intervention assessment compared to the baseline [21, 22]. Estimates for these parameters are based on previous work by Siddiqi K. et al. in a

similar setting among school-going children [23]. Enrolling 40 youth and 100 younger peers would help us estimate recruitment rates of 83.5% with a 95% CI of 69.5% to 97.5% for youth and 76.5% to 90.5% for younger peers. Retention rates would be estimated at 85% with a 95% CI of 66% to 100% for youth and 73% to 97% for younger peers. In addition, the structured diary completion rate among younger peers would be estimated at 56% (95% CI: 46%, 66%).

These estimates will also be used as study progression criteria for designing a definitive RCT; however, we will modify our study data collection methods in the full trial based on the current study findings and observed challenges.

Study measures

The study assessments at different time points throughout the study are presented in Table 3.

Quantitative analysis plan

Descriptive analysis will be reported as mean (standard deviation) or frequencies with percentages as appropriate for participants' characteristics, such as age and gender for youth, age, gender, height, and weight for younger peers, while age, gender, education, and occupation for parents and other stakeholders. We will calculate the recruitment and retention rate of schools, youth, and younger peers, along with their corresponding 95% confidence intervals (CIs). In addition, we will calculate the completion rate (with 95% CIs) of the structured diaries for diet, physical activity, and tobacco use among younger peers at pre- and post-intervention assessments. Furthermore, we will compare the frequency (with 95% CIs) of reducing NCD risk factors among younger peers post-intervention compared to the baseline. Moreover, we will analyze and present the fidelity data descriptively. Data will be analyzed using Stata (V.17.0 StataCorp, Texas, USA).

Data quality management

Data related to NCD risk factors will be collected through structured diaries. Proper instructions will be given to the students on completing the diaries. We will also attach the instruction sheet with the diary for students' guidance. The data collected through structured diaries will be entered into a secure and password-protective database. Access to this data will be limited to the institution's research team, including the PI, co-investigator, and senior research assistants. Prior to data collection, we will conduct extensive training of senior research assistants on data collection processes. We will implement several measures to minimize data entry errors, such as skip pattern checks, variable coding, and checks on the length and type of variables. The PI will oversee the accuracy of data entry and maintain data backup. Data editing and cleaning will be performed simultaneously by the PI. We will keep all identifying information

Table 3 Study assessments at different phases throughout the study

Study activities	Participants involved	Study period					
		Enrolment	Phase I	Phase II	Phase III	Phase IV	Phase V (close-out)
Timepoint		t_1	t_2	t_3	t_4	t_5	t_6
Eligibility screening	Schools, youth and younger peers, their parents, school staff	✓					
Parental consent and assent	Youth, younger peers and their parents	✓					
Informed consent	Youth parents, head teachers, class teachers, canteen staff			✓			
Height	Younger peers		✓				
Weight	Younger peers		✓				
Structured diary	Younger peers		✓				✓
FGDs	Youths and their parents			✓			
IDIs	Stakeholders (head teachers, class teachers, canteen staff)			✓			
Workshop for intervention co-creation	Youth and class teachers				✓		
Training of the intervention	Youth and class teachers					✓	
Delivery of intervention	Youth will deliver the intervention to the younger peers						✓

FGDs Focus-group discussions, *IDIs* In-depth interviews

separate from research data and identify study participants through their unique IDs.

Audio recordings of the qualitative interviews will be handled in the strictest confidence and will only be listened to by research team members. After the interview, the recording will be transferred to a secure computer and deleted from the recorder. The interview will then be transcribed and deleted from the computer.

Protocol amendments

Modification of the study protocol may potentially influence the study's progression. Any amendments considered necessary will be formally proposed to the Ethics Review Committee of the institution following thorough discussion and consensus among the study team. Participants will be notified of any amendments that affect their involvement in the study. Minor modifications that do not impact the study's conduct will not be communicated to participants. Approved amendments will be documented in an updated version of the protocol.

Dissemination plan

We will disseminate our findings to academic and non-academic audiences, including students, educators, parents, policymakers, and the wider community. We will share our study findings through different platforms, such as publishing in peer-reviewed journals, presenting at educational and health conferences, and distributing summary reports to participating schools and stakeholders. This will help public health professionals interpret and implement the findings and boost international collaborations for the future development and/or scale-up of youth-led interventions to reduce the risk of NCDs.

Confidentiality

The study will adhere to ethical guidelines and regulations to protect participants' rights, privacy, and confidentiality. We will obtain informed consent from parents and other stakeholders for the qualitative work. We will obtain assent from youth and younger peers along with parental consent from their guardians through schools. The consent and assent procedure will provide detailed information about the study, its purpose, procedures, potential risks and benefits, and the voluntary nature of participation. Participants will be allowed to ask questions and clarify concerns before consenting. Their participation in the study will be entirely voluntary, and they will have the right to withdraw at any time without facing any consequences. Strict measures will be taken to ensure the confidentiality and anonymity of participants' data throughout the study. All data entered into the electronic database will be identified with a code (study ID) and password protected. The consent, assent, and parental consent forms (hard copies) will be stored securely under lock and key. We will give the participants a copy of the consent and assent form for their records. Participants' names will not be used in any published reports about this study. Data will be stored for 7 years and will be destroyed at the end of the 7 years.

Public health implications

The propose study promises multifaceted benefits beyond raising awareness of NCD risk factors among school-going children. Through active participation, youth will feel empowered and motivated to make informed choices about their health. The shared effort in developing and delivering the intervention will strengthen positive relationships within the community and instill a sense of responsibility in addressing health challenges. Additionally, interventions led by youth will foster capacity-building skills such as leadership, public speaking, and collaboration among the younger generation. In the long run, this increased awareness of the root causes of diseases will help delay the early onset of chronic illnesses, thereby mitigating the burden of diseases. Addressing NCD risk factors at a young age will promote positive behavioral change and contribute to sustained improvements in public health outcomes over time.

Our study does have some limitations. Firstly, we will employ a pre- and post-intervention study design for pragmatic reasons, and the absence of a control group is an important limitation. However, this is an appropriate design to assess the feasibility outcomes and provide meaningful insights to help design future larger trials. Secondly, we will rely on self-reported data on the risk factors of NCDs through structured diaries, which may introduce social desirability bias, where students may provide favorable responses that are socially acceptable, especially for tobacco use, which may lead to under-reporting the risk factors. However, we assure students that we will keep their responses confidential and will not reveal their identity at any stage of the study and during publication to provide more accurate and honest responses. Thirdly, we will assess short-term behavior change regarding the risk factors due to time and resource constraints. However, short-term assessments are valuable for understanding preliminary change, feasibility, and immediate effects of the intervention. Since this study is based on schools from slums, it may limit the generalizability of our study findings to broader school populations, particularly those in higher socioeconomic settings. However, insights from this study can inform a foundation for scaling up similar interventions in other low-resource settings.

Acknowledgements
Not applicable.

Authors' contributions
Conceptualization, SN; funding acquisition, SN; writing—original draft, SN and MI; and writing—review and editing, SN and RI.

Funding
The study is supported by an early career grant award from the Royal Society of Tropical Medicine and Hygiene (RSTMH), funded by the National Institute for Health and Care Research (NIHR) 2023.

Data availability
Not applicable

Declarations

Ethics approval and consent to participate
The study was approved by the Ethical Review Committee of the Aga Khan University (Ref.: 2024-9763-29256). We will obtain written informed consent from parents and other stakeholders for the qualitative work, assent from youth and younger peers, and parental consent from their guardians through schools.

Consent for publication
Not applicable.

Competing interests
The authors declare that they have no competing interests.

Author details
[1]Community Health Sciences Department, The Aga Khan University, Karachi 74800, Pakistan.

Received: 26 June 2024 Accepted: 22 May 2025
Published online: 02 June 2025

References

1. Non-communicable Diseases: World Health Organization; 2023 [updated 16-September-2023. Available from: https://www.who.int/news-room/fact-sheets/detail/noncommunicable-diseases#:~:text=Noncommunicable%20diseases%20(NCDs)%20kill%2041,74%25%20of%20all%20deaths%20globally.
2. Armocida B, Monasta L, Sawyer S, Bustreo F, Segafredo G, Castelpietra G, et al. Burden of non-communicable diseases among adolescents aged 10–24 years in the EU, 1990–2019: a systematic analysis of the Global Burden of Diseases Study 2019. The Lancet Child & Adolescent Health. 2022;6(6):367–83.
3. Gyasi RM, Phillips DR. Aging and the rising burden of non-communicable diseases in sub-Saharan Africa and other low- and middle-income countries: a call for holistic action. Gerontologist. 2019;60(5):806–11.
4. Kazmi T, Nagi M, Razzaq S, Hussnain S, Shahid N, Athar U. Burden of non-communicable diseases in Pakistan. East Mediterr Health J. 2022;28(11):798–804.
5. Sawyer SM, Afifi RA, Bearinger LH, Blakemore S-J, Dick B, Ezeh AC, Patton GC. Adolescence: a foundation for future health. The lancet. 2012;379(9826):1630–40.
6. Organization WH. Global health risks: mortality and burden of disease attributable to selected major risks: World Health Organization; 2009.
7. Sharif H, Sheikh SS, Seemi T, Naeem H, Khan U, Jan SS. Metabolic syndrome and obesity among marginalised school-going adolescents in Karachi, Pakistan: a cross-sectional study. Lancet Reg Health Southeast Asia. 2024;21: 100354.
8. Adeela S, Muhammad Shahid S, Kanwal A, Sophia S. Frequency of overweight and obesity and its associated factors amongst school children in Lahore Pakistan. Proceedings. 2022;36(4):31–6.
9. Pervez S. NMA. Conditions of slums in Pakistan are getting increasingly worse: policy analysis. Term Project, Suleman Dawood School of Business. Published under the Student Research Series 2020 by the Centre for Businessand Society, LUMS2018.
10. Pervez S. NMA. Conditions of slums in Pakistan are getting increasingly worse: Policy Analysis Centre for Business and Society: term project, Suleman Dawood School of Business; 2018 [Available from: https://cbs.lums.edu.pk/student-research-series/conditions-slums-pakistan-are-getting-increasingly-worse-policy-analysis.
11. UN General Assembly. Transforming our world: the 2030 Agenda for Sustainable Development. New York: Division for Sustainable Development Goals.: United Nations; 2015 [Available from: https://sdgs.un.org/publications/transforming-our-world-2030-agenda-sustainable-development-17981.
12. Tarro L, Llauradó E, Aceves-Martins M, Moriña D, Papell-Garcia I, Arola L, et al. Impact of a youth-led social marketing intervention run by adolescents to encourage healthy lifestyles among younger school peers (EYTO-kids project): a parallel-cluster randomised controlled pilot study. J Epidemiol Community Health. 2019;73(4):324–33.
13. Anselma M, Altenburg TM, Emke H, van Nassau F, Jurg M, Ruiter RAC, et al. Co-designing obesity prevention interventions together with children: intervention mapping meets youth-led participatory action research. Int J Behav Nutr Phys Act. 2019;16(1):130.
14. Viner RM, Allen NB, Patton GC. Puberty, developmental processes, and health interventions. In: Bundy DAP, Silva ND, Horton S, Jamison DT, Patton GC, editors. Child and Adolescent Health and Development. Washington (DC): The International Bank for Reconstruction and Development / The World Bank © 2017 International Bank for Reconstruction and Development / The World Bank.; 2017.
15. Moore Heslin A, McNulty B. Adolescent nutrition and health: characteristics, risk factors and opportunities of an overlooked life stage. Proc Nutr Soc. 2023;82(2):142–56.
16. Caraballo L, Lozenski BD, Lyiscott JJ, Morrell E. YPAR and critical epistemologies: rethinking education research. Rev Res Educ. 2017;41(1):311–36.
17. Fernandez ME, Ruiter RAC, Markham CM, Kok G. Intervention mapping: theory- and evidence-based health promotion program planning: perspective and examples. Front Public Health. 2019;7:209.
18. Anselma M, Altenburg T, Chinapaw M. Kids in Action: the protocol of a Youth Participatory Action Research project to promote physical activity and dietary behaviour. BMJ Open. 2019;9(3): e025584.
19. Vandendriessche A, Deforche B, Dhondt K, Altenburg TM, Verloigne M. Combining participatory action research with intervention mapping to develop and plan the implementation and evaluation of a healthy sleep intervention for adolescents. Health Promot Perspect. 2023;13(4):316–29.
20. Braun V, Clarke V. Using thematic analysis in psychology. Qual Res Psychol. 2006;3(2):77–101.
21. Sim J, Lewis M. The size of a pilot study for a clinical trial should be calculated in relation to considerations of precision and efficiency. J Clin Epidemiol. 2012;65(3):301–8.
22. Avery KN, Williamson PR, Gamble C, O'Connell Francischetto E, Metcalfe C, Davidson P, et al. Informing efficient randomised controlled trials: exploration of challenges in developing progression criteria for internal pilot studies. BMJ Open. 2017;7(2): e013537.
23. Siddiqi K, Huque R, Kanaan M, Ahmed F, Ferdous T, Shah S, et al. Children Learning About Secondhand Smoke (CLASS II): a pilot cluster randomized controlled trial. Nicotine Tob Res. 2019;21(5):670–7.

Publisher's Note
Springer Nature remains neutral with regard to jurisdictional claims in published maps and institutional affiliations.
